# British Dietetic Association consensus guidelines on the nutritional assessment and dietary management of patients with inflammatory bowel disease

**DOI:** 10.1111/jhn.13054

**Published:** 2022-07-21

**Authors:** Miranda C. E. Lomer, Bridgette Wilson, Catherine L. Wall

**Affiliations:** ^1^ Department of Nutrition and Dietetics Guy's and St Thomas' NHS Foundation Trust London UK; ^2^ Department of Nutritional Sciences King's College London London UK; ^3^ Department of Medicine University of Otago Christchurch New Zealand

**Keywords:** diet, Crohn's disease, inflammatory bowel disease, nutritional assessment, ulcerative colitis

## Abstract

**Background:**

Despite increased awareness of diet and nutrition being integral to the management of patients with inflammatory bowel disease (IBD), there are gaps in the knowledge of IBD healthcare providers regarding nutrition. Furthermore, high quality evidence on nutritional assessment and dietary management of IBD is limited. A Delphi consensus from a panel of experts allows for best‐practice guidelines to be developed, especially where high quality evidence is limited. The aim was to develop guidelines for the nutritional assessment and dietary management of IBD using an eDelphi online consensus agreement platform.

**Methods:**

Seventeen research topics related to IBD and nutrition were systematically reviewed. Searches in Cochrane, Embase®, Medline® and Scopus® electronic databases were performed. GRADE was used to develop recommendations. Experts from the IBD community (healthcare professionals and patients with IBD) were invited to vote anonymously on the recommendations in a custom‐built online platform. Three rounds of voting were carried out with updated iterations of the recommendations and evaluative text based on feedback from the previous round.

**Results:**

From 23,824 non‐duplicated papers, 167 were critically appraised. Fifty‐five participants completed three rounds of voting and 14 GRADE statements and 42 practice statements achieved 80% consensus. Comprehensive guidance related to nutrition assessment, nutrition screening and dietary management is provided.

**Conclusions:**

Guidelines on the nutritional assessment and dietary management of IBD have been developed using evidence‐based consensus to improve equality of care. The statements and practice statements developed demonstrate the level of agreement and the quality and strength of the guidelines.

## Key points

- Comprehensive guidelines related to nutrition assessment, nutrition screening and dietary management of inflammatory bowel disease have been developed.
- Fourteen GRADE statements and 42 practice statements achieved 80% consensus agreement
- Fifty‐five participants comprising experts from the inflammatory bowel disease (IBD) community (healthcare professionals and patients with IBD) took part in the online consensus voting

## Graphical abstract

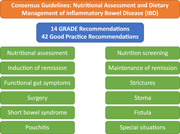

## INTRODUCTION

Despite increased awareness of diet and nutrition being integral to the management of patients with inflammatory bowel disease (IBD), there are still gaps in the knowledge of IBD healthcare providers regarding nutrition.^1^, ^2^ Furthermore, high quality evidence on the nutritional assessment and dietary management of patients with IBD is limited. Consensus from a panel of experts known as a Delphi process allows for best‐practice guidelines to be developed, especially where high quality evidence is limited. The aim of this research was to develop guidelines for the nutritional assessment and dietary management of IBD using an eDelphi online consensus agreement platform (https://www.edelphi.org).

## METHODS

An expert IBD panel including two patients, clinicians and researchers was created to discuss pertinent topics related to diet and IBD to include in the guideline. Sixteen research topics were identified from the research literature, clinical practice and gaps in the evidence base (see Supporting information, Table S1).

### Search strategy

Systematic reviews for each topic were undertaken in line with recommendations of the Cochrane Handbook for Systematic Reviews of Interventions^3^ and reported in line with the guidelines of Preferred Reporting Items for Systematic reviews and Meta‐Analyses.^4^ A Population, Intervention, Comparison and Outcomes framework and literature search strategy criteria were developed (see Supporting information, Tables S2 and S3) to search Cochrane, Embase®, Medline® and Scopus® electronic databases for topics on nutritional assessment and dietary management of IBD up until March 2022. Identification, screening, eligibility and inclusion of eligible papers were agreed between the researchers in advance and published prior to the literature searches being conducted (PROSPERO 2018 CRD42018096818; PROSPERO 2019 CRD42019138650).

References were imported into a bibliographic database and duplicates were removed automatically (EndNote X9; Thomson Reuters). Titles and abstracts were screened against the PICO eligibility criteria and potentially eligible full text articles were then obtained and screened against the eligibility criteria by two researchers independently. Reference lists of potential studies were cross‐referenced for other studies of potential relevance. The percentage agreement in study eligibility and a kappa statistic were calculated to check concordance between reviewers.^3^ Disparities in study eligibility were resolved through discussion with a third researcher.

Reasons for excluding studies are provided (see Supporting information, Table S4).

### Data extraction and risk of bias

Data were extracted from each eligible study relating to the patient or group, the intervention, the comparator, outcomes measured and the study design. A standardised data extraction sheet was developed, and two reviewers extracted the data from eligible papers. Discrepancies were reviewed and resolved.

Risk of bias tools appropriate to the study design were used. Two reviewers independently assessed risk of bias using seven domains: adequacy of randomisation, allocation concealment, blinding methods, complete outcome data, selective reporting and other sources of bias.^3^ Any disparities were resolved through discussion with a third reviewer.

Where data were available from randomised controlled trials (RCTs), this was used to answer the research question and lower quality studies were not considered when developing evidence statements. Where no RCT data were available lower quality studies were considered when developing evidence statements.

### GRADE approach

Research papers were critically appraised using the GRADE tool.^5^ GRADE analysis of the evidence was performed where two or more studies reported data for the same outcome. For dichotomous outcomes (e.g., remission), frequencies were entered to obtain an odds ratio (OR). For continuous outcomes (e.g., disease activity score), standardised mean differences were calculated.

To address the research questions where data were expected to be available for a range of interventions, dietary interventions were categorised and each of these were separately assessed for available outcomes using the GRADE approach. The categories of nutrition outcome were; complementary alternative medicine, elimination diets, enteral nutrition, fibre (including prebiotics), nutrients, probiotics and whole diets.

Where a recently published systematic review was available, data from this were combined with data from studies published subsequent to the last search date of the systematic review.

Furthermore, where evidence was of low quality and GRADE Statements were not possible, recommendations to inform clinical practice were developed as Practice Statements.

### Consensus

GRADE Statements and Practice Statements underwent consensus voting using an eDelphi online platform. IBD experts from British Dietetic Association Gastroenterology Specialist Group, British Society of Gastroenterology, Crohn's and Colitis UK and patients with IBD were invited to vote anonymously on the statements.

Three rounds of voting took place. Participants used a five‐point Likert scale to vote (strongly disagree, disagree, neutral, agree, strongly agree). After each voting round, statements that did not reach 80% consensus were reformulated and taken to the next round of voting or removed. In rounds 2 and 3, updated iterations of the statements were voted on and evaluative text based on feedback from the previous round was available.

## RESULTS

From 23,824 non‐duplicated papers, 167 were critically appraised (see Supporting information, Table S5). Overall, the evidence was of generally low quality and risk of bias assessment is provided for all studies linked to GRADE statements but not practice statements (Table 1).

**Table 1:** Risk of bias

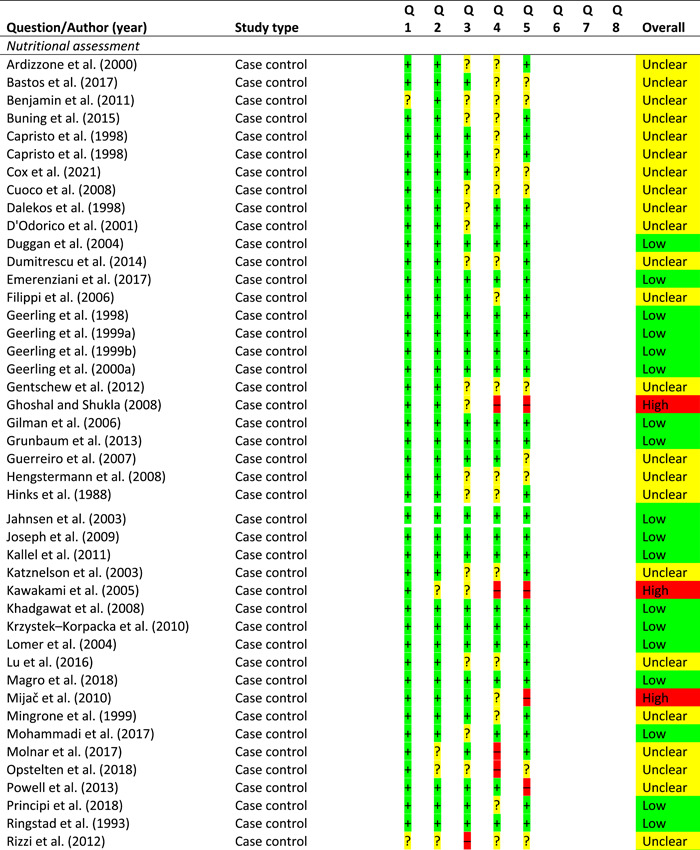
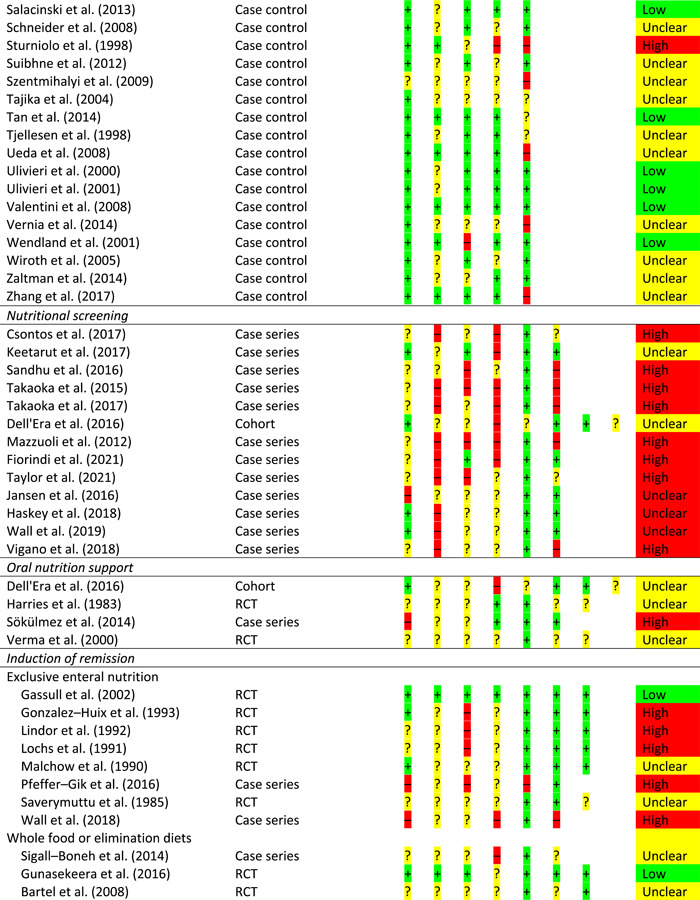
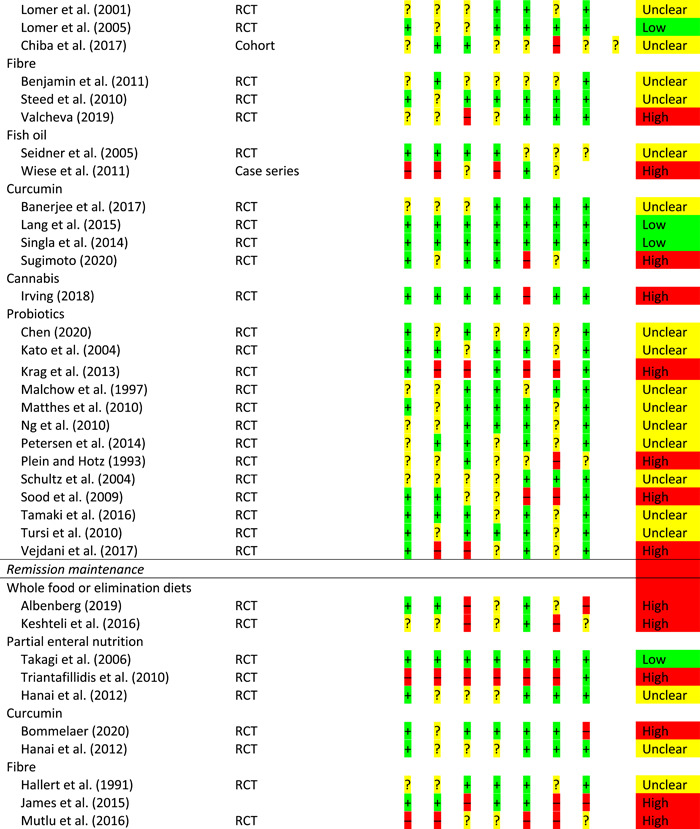
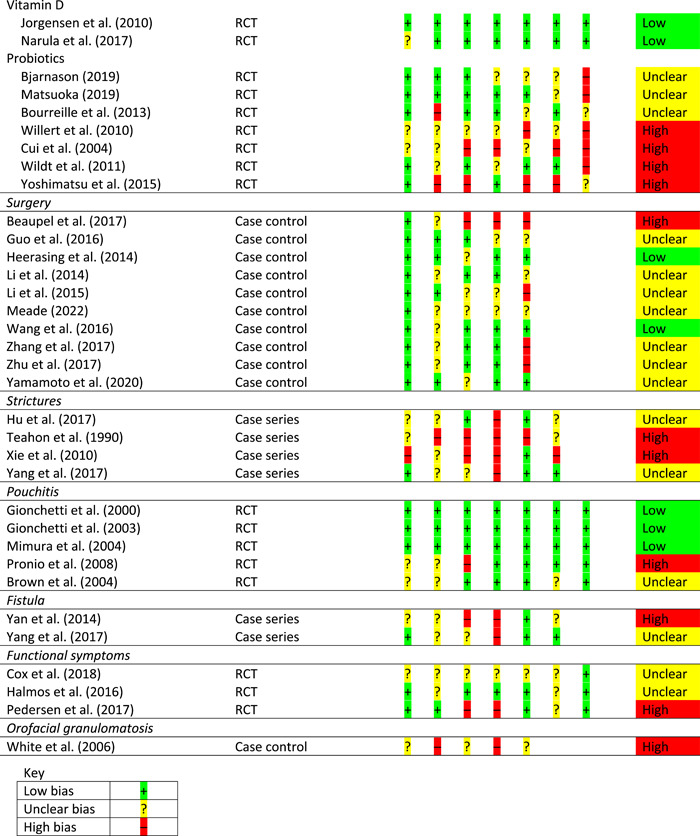

*Note*: For RCTs, there are seven risk of bias questions; for cohort studies, there are eight risk of bias questions; for case–control studies, there are five risk of bias questions; for case series, there are six risk of bias questions.

Abbreviation: RCT, randomised controlled trial.

Fifty‐five out of an initial 114 participants completed three rounds of voting and 14 GRADE statements and 42 practice statements achieved 80% consensus (Table 2). Comprehensive guidance related to nutrition assessment, nutrition screening and dietary management [induction of disease remission, maintenance of disease remission, functional gut symptoms, strictures, surgery, stoma, short bowel syndrome, fistula, pouchitis and special situations; e.g., orofacial granulomatosis (OFG)] are provided below. Four GRADE statements and nine practice statements were removed as a result of not reaching the consensus cut off.

**Table 2:** Grade recommendations and % consensus

GRADE recommendations		% consensus
Nutrition assessment		
	Fibre intake is likely to be low in inflammatory bowel disease and should be included as part of a nutritional assessment. (GRADE very low quality)	88.1
	Calcium intake should be assessed in Crohn's disease and ulcerative colitis patients because patients may not meet their recommended intake. (GRADE very low quality)	96.3
	Iron intake should be assessed in Crohn's disease and ulcerative colitis patients as patients may not meet their recommended intake. (GRADE very low quality)	96.2
Nutrition screening		
	MUST may be used to screen patients with inflammatory bowel disease for risk of malnutrition. (GRADE very low quality)	85.5
Induction of remission		
	To induce remission in active Crohn's disease, exclusive enteral nutrition (EEN) is less effective than corticosteroids. EEN may be used in mild to moderate disease where avoidance of corticosteroids is intended, and dietetic expertise is available. (GRADE very low quality)	84.6
	In some patients with mildly active ulcerative colitis, taking specific probiotics alongside usual medication may support induction of disease remission. (GRADE moderate quality)	88.7
Remission maintenance		
	There is insufficient evidence to support the use of high dose vitamin D for maintaining remission in Crohn's disease. (GRADE very low quality)	81.6
	Partial enteral nutrition alongside routine medication may support Crohn's disease remission maintenance. (GRADE very low quality)	88.0
	A probiotic mixture of 8 bacterial strains may maintain remission in chronic relapsing pouchitis. Very limited evidence may support prophylactic use after pouch formation surgery to prevent initial pouchitis onset. There is no evidence to support the use of other probiotics to maintain remission in pouchitis. (GRADE low quality)	85.4
Surgery		
	There is limited evidence that pre‐surgical exclusive enteral nutrition may reduce the length of post‐surgical hospitalisation in patients with Crohn's disease. (GRADE very low quality)	84.3
	Pre‐surgical exclusive enteral nutrition may lower the risk of post‐surgical infectious complications in patients with Crohn's disease. (GRADE very low quality)	92.0
Stricturing disease		
	Exclusive enteral nutrition for 4–12 weeks may induce remission in Crohn's disease patients with inflammatory strictures. (GRADE very low quality)	84.3
Fistulating disease		
	There is very limited evidence that fistulating Crohns disease may respond to exclusive enteral nutrition. (GRADE very low quality)	82.0
Functional symptoms		
	A low FODMAP diet may improve global functional bowel symptoms in quiescent or mildly active inflammatory bowel disease. (GRADE very low quality)	90.2

Abbreviations: CD, Crohn's disease; IBD, inflammatory bowel disease; UC, ulcerative colitis.

## NUTRITION ASSESSMENT: ANTHROPOMETRY AND BODY COMPOSITION

**Practice Statement**

Disease activity affects body composition in inflammtory bowel disease; therefore, it is desirable to assess nutritional status longitudinally using body mass index in combination with assessments of body composition and/or muscle function. Agreement 96.2%.

Anthropometric measurement of nutrition assessment in patients with IBD in clinical practice is challenging and most often relies solely on weight and body mass index (BMI). Systemic inflammation affects body composition, often with a reduction in muscle mass (i.e., fat free mass) and an increase in fat mass. Sarcopenia is common in IBD, even in overweight patients,^6^ and relying on weight and BMI may mask changes in body composition; thus, other anthropometric measures available in clinical practice are important in IBD and were systematically reviewed.

### BMI

Thirty studies reported BMI, 11 studies matched healthy controls and IBD patients for BMI so were excluded from the GRADE analysis.^7^, ^8^, ^9^, ^10^, ^11^, ^12^, ^13^, ^14^, ^15^, ^16^, ^17^ The remaining 19 studies included 1077 patients with Crohn's disease (CD), 426 patients with UC and 4255 healthy controls.^18^, ^19^, ^20^, ^21^, ^22^, ^23^, ^24^, ^25^, ^26^, ^27^, ^28^, ^29^, ^30^, ^31^, ^32^, ^33^, ^34^, ^35^, ^36^

#### Crohn's disease

Eight studies reported BMI in remission in CD.^18^, ^19^, ^20^, ^21^, ^22^, ^26^, ^33^, ^35^

Four studies found BMI was similar between patients with CD in remission (*n* = 151) and healthy controls (*n* = 193)^21^, ^22^, ^26^, ^35^ and four studies found BMI was lower in patients with CD in remission (*n* = 266) compared to healthy controls (*n* = 225)^18^, ^19^, ^20^, ^33^

One study reported BMI in active CD^18^ and a further six studies in active CD and CD in remission.^23^, ^24^, ^27^, ^30^, ^34^, ^36^

Four studies found BMI was similar between patients with active CD or CD in remission (*n* = 163) and healthy controls (*n* = 174)^23^, ^24^, ^27^, ^34^ and two studies found BMI was lower in patients with active CD or CD in remission (*n* = 286) compared to healthy controls (*n* = 2006).^30^, ^36^

#### Ulcerative colitis

Two studies assessed BMI in ulcerative colitis (UC) patients in remission.^20^, ^26^ Both studies found BMI was similar between patients with UC in remission (*n* = 37) and healthy controls (*n* = 68). One study in active UC found BMI was lower in patients (*n* = 53) compared to healthy controls (*n* = 30).^29^

Four studies assessed BMI in active UC and UC in remission.^24^, ^27^, ^28^, ^34^ Two studies found similar BMI between patients (*n* = 41) and healthy controls (*n* = 43)^28^, ^34^ and two studies found BMI was lower in active UC and UC in remission (*n* = 106) compared to healthy controls (*n* = 106).^24^, ^27^

#### IBD

Four studies assessed BMI in IBD patients.^25^, ^29^, ^31^, ^32^ Three studies found BMI was significantly lower in active IBD and IBD in remission (*n* = 303) compared to healthy controls (*n* = 1540)^25^, ^29^, ^31^ and one found BMI was similar between patients with IBD in remission (*n* = 150) and healthy controls (*n* = 100).^32^

### Fat mass

Seventeen studies assessed fat mass in 920 CD patients, 195 UC patients and 983 healthy controls.^8^, ^14^, ^15^, ^16^, ^17^, ^18^, ^19^, ^20^, ^21^, ^22^, ^23^, ^24^, ^27^, ^33^, ^35^, ^36^, ^37^

#### Crohn's disease

Two studies assessed fat mass in active CD.^8^, ^18^ There were 56 CD patients and 120 healthy controls in the analysis. One study showed that fat mass was significant lower in active CD compared to healthy controls,^18^ whereas the other study did not.^8^

Nine studies assessed fat mass in CD in remission.^16^, ^17^, ^18^, ^19^, ^20^, ^21^, ^22^, ^33^, ^35^ Fat mass was significantly lower in CD in remission (*n* = 115) compared to healthy controls (*n* = 100) in three studies ^19^, ^20^, ^21^ and the remaining six studies found no differences between groups: patients (*n* = 403) and healthy controls (*n* = 356)^16^, ^17^, ^18^, ^22^, ^33^, ^35^

Six studies assessed fat mass in active CD and CD in remission.^14^, ^15^, ^23^, ^24^, ^27^, ^36^ There were no differences in fat mass between the patients (*n* = 143) and healthy controls (*n* = 143) in three studies^23^, ^24^, ^27^ and fat mass was lower in patients (*n* = 246) compared to healthy controls (*n* = 341) in the other three studies.^14^, ^15^, ^36^

#### Ulcerative colitis

Two studies assessed fat mass in UC in remission.^16^, ^20^ There were no differences in fat mass between the patients (*n* = 16) and healthy controls (*n* = 20) in one study^20^ and fat mass was higher in patients (*n* = 50) compared to healthy controls (*n* = 61) in the other.^16^

Three studies assessed fat mass in active UC and UC in remission.^24^, ^27^, ^37^ All studies found no differences in fat mass between the patients (*n* = 129) and healthy controls (*n* = 106).

### Percentage fat mass

Fifteen studies assessed percentage fat mass in IBD with 664 CD patients, 190 UC patients and 675 healthy controls.^7^, ^12^, ^13^, ^17^, ^18^, ^19^, ^20^, ^22^, ^23^, ^24^, ^27^, ^29^, ^33^, ^35^, ^38^

#### Crohn's disease

Two studies assessed percentage fat mass in active CD (*n* = 152) and showed that percentage fat mass was significantly lower compared to healthy controls (*n* = 130).^18^, ^29^

Eight studies assessed percentage fat mass in CD in remission. Two studies showed that percentage fat mass was significantly lower in patients (*n* = 61) compared to healthy controls (*n* = 75),^19^, ^20^ one study showed that percentage fat mass was significantly higher in patients (*n* = 31) compared to healthy controls (*n* = 98)^35^ and the remaining five studies showed similar percentage fat mass between groups: patients (*n* = 299) and healthy controls (*n* = 272).^17^, ^18^, ^22^, ^33^, ^38^

Six studies assessed percentage fat mass in active CD and CD in remission. Two studies showed that percentage fat mass was significantly higher in patients (*n* = 51) compared to healthy controls (*n* = 39)^7^, ^12^

And the remaining four studies showed similar percentage fat mass between groups: patients (*n* = 243) and healthy controls (*n* = 199)^13^, ^23^, ^24^, ^27^

#### Ulcerative colitis

One study assessed percentage fat mass in active UC and showed it was lower in patients (*n* = 53) compared to healthy controls (*n* = 30).^29^

Two studies assessed percentage fat mass in UC in remission and showed no differences between patients (*n* = 31) and healthy controls (*n* = 85).^20^, ^38^

Two studies assessed percentage fat mass in active UC and UC in remission. One study showed that percentage fat mass was significantly higher in patients (*n* = 60) compared to healthy controls (*n* = 60)^27^ and the other study showed similar percentage fat mass between groups: patients (*n* = 46) and healthy controls (*n* = 46).^24^

### Fat free mass

Fat free mass was measured in 18 studies ^8^, ^9^, ^14^, ^15^, ^16^, ^17^, ^18^, ^19^, ^20^, ^21^, ^22^, ^23^, ^24^, ^27^, ^33^, ^35^, ^37^, ^38^ in 836 CD patients, 210 UC patients and 814 healthy controls and analysis demonstrated inconsistent findings.

#### Crohn's disease

Two studies assessed fat free mass in active CD and both found that fat free mass was significantly lower in patients (*n* = 1366) compared to healthy controls (*n* = 120).^8^, ^18^

Ten studies assessed fat free mass in CD in remission. Two studies found fat free mass was significantly lower in patients (*n* = 154) compared to healthy controls (*n* = 188),^18^, ^35^ and one study reported lower fat free mass for males (*n* = 33) but not females (*n* = 61) when patients were compared with healthy controls (males *n* = 20, females *n* = 41).^16^ The remaining seven studies found fat free mass was similar between patients (*n* = 291) and healthy controls (*n* = 272).^17^, ^19^, ^20^, ^21^, ^22^, ^33^, ^38^

Six studies assessed fat free mass in a mixed population of active CD and CD in remission. Four studies found that fat free mass differed between patients (*n* = 179) and healthy controls (*n* = 170).^9^, ^24^, ^27^

The most recent study separated patients into those on conventional medication (*n* = 22) and those on a biologic (*n* = 23) and, when compared with healthy controls (*n* = 20), found lower fat free mass for patients on conventional medication but not on a biologic.^9^

One study found that patients with newly diagnosed CD (*n* = 20) had higher fat free mass compared to healthy controls (*n* = 20) but patients who had CD for more than 5 years (*n* = 30) had lower fat free mass compared to healthy controls (*n* = 40).^23^

One study analysed patients as one group and found higher fat free mass in the patients (*n* = 23) compared to healthy controls (*n* = 23)^24^ and another found that fat free mass was lower in the patients (*n* = 60) compared to healthy controls (*n* = 60).^27^ The remaining two studies found no differences in fat free mass between groups (patients *n* = 96, healthy controls *n* = 87).^14^, ^15^

#### Ulcerative colitis

No studies assessed fat free mass in active UC.

Three studies assessed fat free mass in UC in remission. In two studies, there was no difference between patients (*n* = 31) and healthy controls (*n* = 85).^20^, ^38^ In the third study, male patients (*n* = 17) had lower fat free mass compared to male healthy controls (*n* = 20) but female patients (*n* = 33) had similar fat free mass compared to healthy controls (*n* = 41).^16^

Three studies assessed fat free mass in a mixed population of active UC and UC in remission and found no differences in fat free mass between patients (*n* = 129) and healthy controls (*n* = 106).^24^, ^27^, ^37^

### Percentage fat free mass

Two studies assessed percentage fat free mass in 61 CD patients in remission, 16 UC patients in remission and 75 healthy controls.^19^, ^20^ One study showed that percentage fat free mass was lower in patients (*n* = 43) compared to healthy controls (*n* = 55),^19^ whereas the other study showed similar percentage fat free mass between groups: patients (*n* = 18) and health controls (*n* = 20).^20^

No studies in UC were identified.

### Visceral adipose tissue

Three studies assessed visceral adipose tissue in a mixed population of active CD and CD in remission with 101 CD patients and 67 healthy controls.^7^, ^12^, ^13^ All studies found visceral adipose tissue was higher in patients compared to healthy controls.

No studies in UC were identified.

### Waist to hip ratio

Two studies assessed waist to hip ratio, one in CD in remission^22^ and the other in a mixed population of active CD and CD in remission.^12^ Fifty patients with CD and 52 healthy controls were included in the analysis. Both studies found no difference in waist to hip ratio between patients and healthy controls.^12^, ^22^

### Muscle strength

In health, muscle mass is a strong predictor of muscle strength. In IBD, muscle strength is likely to be affected by disease activity. Longitudinal assessment of muscle function can be useful for assessing long‐term effects of disease activity on muscle strength and endurance.

Handgrip and lower limb strength are reliable measures of muscle function; however, in clinical practice, handgrip strength is more practical, quicker and cheap assessment tool compared to assessment of lower limb strength.

Muscle strength was assessed in seven studies.^15^, ^16^, ^17^, ^22^, ^24^, ^36^, ^39^ There were 437 patients with CD, 96 patients with UC and 491 healthy controls with inconsistent results.

#### Crohn's disease

One study found hamstring and quadricep muscle strength was lower in male patients with CD in remission (*n* = 14) compared to healthy controls (*n* = 14) but there was no difference in female patients (*n* = 18) and healthy controls (*n* = 18).^22^

One study assessed hamstring and quadricep muscle strength in a mixed population of active CD and CD in remission and found similar strength between patients (*n* = 23) and healthy controls (*n* = 23).^24^

One study found knee extension and flexion peak torque were lower in patients with CD in remission (*n* = 19) compared to healthy controls (*n* = 19) and found fatigues rates were higher in patients compared to healthy controls.^39^

Two studies assessed handgrip strength in patients with CD in remission. One study found handgrip strength to be lower in patients (*n* = 94) compared to healthy controls (*n* = 61),^16^ whereas the other found handgrip strength was lower in male patients (*n* = 17) compared to male healthy controls (*n* = 10) but not female patients (*n* = 24) compared to female healthy controls (*n* = 15).^17^ The latter study found that handgrip endurance was lower in female patients but not male patients compared to healthy controls.

Two studies assessed handgrip strength in a mixed population of active CD and CD in remission and found handgrip strength was lower in patients (*n* = 78) compared to healthy controls (*n* = 75).^15^, ^36^

#### Ulcerative colitis

One study assessed hamstring and quadricep muscle strength in a mixed population of active UC and UC in remission and found similar strength between patients (*n* = 46) and healthy controls (*n* = 46).^24^

One study in UC in remission assessed handgrip strength and found that it was lower in patients (*n* = 50) compared to healthy controls (*n* = 61).^16^

#### Practice tips

Table 3 provides information on available nutritional assessment anthropometric tools. A practical guide on how to use different anthropometric tools in nutritional assessment is presented elsewhere.^41^ Suboptimal mean age and sex specific handgrip strength values are provided in Tables 3, 4, 5.

**Table 3:** Tools for nutritional assessment in inflammatory bowel disease

Body mass index (BMI)	BMI uses weight and height to calculate if weight is healthy. When used in isolation, BMI provides limited data for nutritional assessment. Relying on weight and BMI may mask IBD related changes in fat mass and fat–free mass
Handgrip strength	A reliable measure of muscle strength and muscle reserve
Mid–upper arm circumference (MUAC)	A measure of nutritional depletion that is less affected by fluid status
Tricep skinfold thickness (TSF)	A measure of fat mass that is more reliable than BMI
Mid‐arm muscle circumference	A measure of fat–free mass derived from MUAC and TSF
Bioelectrical impedance	A measure of fat mass and fat–free mass
Waist to hip ratio	A measure of abdominal fat
Dual‐energy X‐ray absorptiometry	A measure of bone mineral density
Novel radiological scans	Computerised tomography^40^ and magnetic resonance imaging can be used to measure abdominal fat mass

*Note*: Further practical advice is available in Sandall et al.^41^

**Table 4:** Evaluation of hand grip strength (HGS)

Males			Females		
Age (years)^42^	Mean HGS (kg)	Suboptimal	Age^42^	Mean HGS (kg)	Suboptimal
15–19	29.6	<25.2	15–19	23.9	<20.3
20–24	41.5	<35.3	20–24	28.4	<24.1
25–29	48.8	<41.5	25–29	30.6	<26.0
30–34	51.6	<43.9	30–34	31.4	<26.7
35–39	51.6	<43.9	35–39	31.3	<26.6
40–44	50.3	<42.8	40–44	30.7	<26.1
45–49	48.8	<41.5	45–49	29.9	<25.4
50–54	47.6	<40.5	50–54	28.7	<24.4
55–59	46.2	<39.3	55–59	27.5	<23.4
60–64	44.6	<37.9	60–64	26.5	<22.5
65–69	42.3	<36.0	65–69	25.3	<21.5
70–74	39.1	<33.2	70–74	23.5	<20.0
75–79	35.6	<30.3	75–79	21.4	<18.2
80–84	32.2	<27.4	80–84	19.1	<16.2
85–89	28.5	<24.2	85–89	16.6	<14.1
90–94	24.7	<21.0	90–94	14.2	<12.1
95+	No data		95+	No data	

Age and sex specific mean values for the maximum value of available attempts taken with permission from Dodds et al.^43^ Suboptimal HGS is mean HGS (three attempts) that is less than 85% of the mean for age and sex.^44^

**Table 5:** Recommendations for monitoring of micronutrient status in IBD

Nutrient	Remission	Active disease (outpatients)	Reference
Iron	Full blood count, serum ferritin, C‐reactive protein every 6–12 months	Every 3 months	Dignass et al.^45^
Folic acid	Annually (if at risk – small bowel disease or resection).	Annually (if at risk – small bowel disease or resection)	Dignass et al.^45^
	Sulfasalazine and methotrexate impair folate absorption		
Vitamin B_12_	Annually (if at risk – ileocaecal resection, vegan or avoiding meat and dairy)	Annually (if at risk – small bowel disease or resection)	Sandall et al.,^41^ Dignass et al.^45^
Folic acid and vitamin B_12_	Every 3–6 months (if at risk – small bowel disease or resection)	Every 3–6 months (if at risk – small bowel disease or small bowel resection)	Maaser et al.^46^
Vitamin D	Seasonal variation will influence interpretation	Measure in those with active disease – supplement and then re‐measure to check they have responded	Sandall et al.,^41^ Maaser et al.^46^
Vitamin K, selenium, vitamin A, vitamin C, zinc, vitamin B_6_, vitamin B_1_	Malabsorption parameters should be assessed at regular intervals in all patients with IBD		Maaser et al.^46^
	Only in clinical scenario that could be exacerbated by micronutrient deficiency (e.g., poor wound healing) or in patients at greater risk (e.g., small bowel disease or resection)		

Abbreviation: IBD, inflammatory bowel disease.

Multidisciplinary team meetings are a vital part of the patient care pathway. From a nutritional assessment perspective, body composition, muscle strength and bone mineral density may be altered, particularly when patients are taking corticosteroids, thus nutritional concerns and management should be discussed as part of the IBD MD team.^47^

## NUTRITION ASSESSMENT: MICRONUTRIENTS

**Practice Statement**

In inflammatory bowel disease, clinical interpretation of some serum micronutrients may be unreliable during inflammation. Agreement 96.2%.

One study has provided what C‐reactive protein (CRP) levels are useful for clinical interpretation of certain micronutrients^48^:

CRP < 20 mg L^–1^ plasma zinc;

CRP < 10 mg L^–1^ plasma selenium, vitamins A and D;

CRP < 5 mg L^–1^ vitamin B6 and vitamin C

This means that where CRP is above the cut‐off provided, the micronutrient level may not be accurate. Further information is available.^49^, ^50^

**Practice Statement**

A comprehensive nutritional assessment in inflammatory bowel disease patients includes the following serum micronutrients: folic acid, vitamin B_12_, vitamin D, iron, zinc, magnesium and selenium. Review recent tests and determine the frequency of monitoring depending on disease activity, dietary intake and micronutrient supplementation. Agreement 90.2%.

In clinical practice, only patients with micronutrient deficiencies are treated with supplementation, however, most studies only report serum concentrations of micronutrients and not deficiency rates which is a major limitation of the current research literature. Only two studies report on micronutrient deficiency rates for patients and healthy controls.^16^, ^51^ The nutrients selected are based on the available literature.

### Folic acid

Five studies assessed serum folic acid and/or folic acid deficiency rates.^16^, ^22^, ^24^, ^51^, ^52^

The studies included 283 patients with CD, 189 patients with UC and 272 healthy controls. One study reported that they excluded patients who were receiving folic acid supplementation within the previous 6 months,^52^ two studies included patients who took folic acid supplements ^16^, ^22^ and two studies did not describe whether patients received folic acid supplementation or not.^24^, ^51^

#### Crohn's disease

All four studies demonstrated there were no differences in serum folic acid levels between healthy controls and patients with CD in remission ^22^ or mixed populations of patients with active CD or CD in remission.^24^, ^51^, ^52^

One study found similar folic acid deficiency rates for CD patients in remission in one of 94 (1.0%) and no healthy controls (0%) (*p* > 0.05).^16^ Another study reported folic acid deficiency rates were significantly higher at 30 of 105 (28.8%) in CD patients compared to two of 60 (3.7%) in healthy controls (*p* < 0.001).^51^

#### Ulcerative colitis

Two studies assessed serum folic acid levels between healthy controls and mixed populations of patients with active UC or UC in remission and found no differences between groups.^24^, ^51^

One study found similar folic acid deficiency rates for UC patients in remission (1/54 [(2.0%]) and no healthy controls (0%) (*p* > 0.05).^16^ One study reported folic acid deficiency rates were eight of 99 (8.6%) in UC patients compared to two of 60 (3.7%) in healthy controls, these rates were not significantly different (*p* = 0.278).^51^

### Vitamin B_12_

Five studies assessed serum vitamin B_12_ and/or vitamin B_12_ deficiency rates.^16^, ^22^, ^24^, ^51^, ^52^

Micronutrient deficiency rates were reported in CD patients and healthy controls in two studies.^16^, ^51^

The studies included 283 patients with CD, 189 patients with UC and 272 healthy controls. One study reported that they excluded patients who had received vitamin B_12_ supplementation within the previous 6 months,^52^ two studies included patients who had received intramuscular vitamin B_12_ ^16^, ^22^ and two studies did not report whether patients did or did not receive vitamin B_12_ supplementation.^24^, ^51^

#### Crohn's disease

One study reported similar serum vitamin B_12_ levels between CD patients (*n* = 32) in remission and healthy controls (*n* = 32).^22^ Another study also reported similar serum vitamin B_12_ levels but included a mixed population (*n* = 45) of active CD and CD patients in remission compared to healthy controls (*n* = 53).^51^ Two studies reported significantly lower serum vitamin B_12_ levels for mixed populations of patients (*n* = 112) with active CD or CD in remission compared to healthy controls (*n* = 126).^24^, ^52^

One study found similar vitamin B_12_ deficiency rates for CD patients in remission (9/94 [9.6%]) and no healthy controls (0%) (*p* > 0.05).^16^ One study reported vitamin B_12_ deficiency rates were significantly higher at 10 of 45 (22.2%) in a mixed population of CD patients compared to four of 53 (7.5%) in healthy controls (*p* = 0.039).^51^ rates.

#### Ulcerative colitis

Two studies assessed serum vitamin B_12_ levels between mixed populations of patients with active UC or UC in remission (*n* = 139) and healthy controls (*n* = 76) and found no differences between groups.^24^, ^51^

One study found similar vitamin B_12_ deficiency rates for UC patients in remission (4/50 [8.0%]) and no healthy controls (0%) (*p* > 0.05).^16^ One study reported similar vitamin B_12_ deficiency rates of seven of 93 (7.5%) for mixed populations of patients with active UC or UC in remission and healthy controls (4/53 [7.5%]) (*p* = 0.971).^51^

### Vitamin D

There are 11 studies (five in remission and six in mixed IBD) measuring vitamin D status.

#### Crohn's disease

Eleven studies, five in remission^10^, ^26^, ^38^, ^39^, ^53^ and six in mixed CD, were included.^54^, ^55^, ^56^, ^57^, ^58^, ^59^

Five studies showed that CD with active disease or CD in remission (*n* = 267) did have significantly lower vitamin D compared to healthy controls (*n* = 394)^10^, ^53^, ^55^, ^56^, ^59^ and six studies showed similar levels (CD, *n* = 239; HC, *n* = 247).^26^, ^38^, ^39^, ^54^, ^57^, ^58^

Nine studies assessed vitamin D deficiency using different cut offs for suboptimal vitamin D status:

Five studies used <50 nmol L^–1^ and one study found significantly more CD patients (27/34 [79%]) had vitamin D deficiency compared to healthy controls (17/34 [50%]) (*p* < 0.05)^56^ and the others did not report a difference.^26^, ^39^, ^55^, ^57^

Two studies in CD in remission used <40 nmol L^–1^. One found more patients [9/47 [19.1%])^10^ had a higher vitamin D deficiency rate compared to healthy controls (2/47 [4.3%]) (*p* < 0.05) and the other found similar rates.^53^

One study used <25 nmol L^–1^ and found similar rates of vitamin D deficiency between patients (*n* = 33) and healthy controls (*n* = 15).^58^

One study used <25 nmol L^–1^ in winter and <70 nmol L^–1^ in summer/autumn and found significantly more patients with CD in remission (18/32 [56%]) had vitamin D deficiency compared to healthy controls (9/32 [28%]) (*p* < 0.01).^22^

#### Ulcerative colitis

Five studies, three in remission UC^10^, ^26^, ^38^ and two in mixed UC^55^, ^59^ were included.

Three studies showed that vitamin D in active UC or UC in remission (*n* = 189) was lower than healthy controls (*n* = 316)^10^, ^55^, ^59^ and two studies showed similar levels (UC, *n* = 36; HC, *n* = 113).^26^, ^38^

Three studies assessed vitamin D deficiency using different cut offs for suboptimal vitamin D status.

Two studies used <50 nmol L^–1^ and both found similar vitamin D deficiency rates between patients with UC and healthy controls.^10^, ^26^, ^38^, ^39^, ^53^, ^54^, ^55^, ^56^, ^57^, ^58^, ^59^

One study in UC in remission used <40 nmol L^–1^ and found similar vitamin D deficiency rates between patients with UC and healthy controls.^10^, ^53^, ^55^, ^56^, ^59^

### Zinc

Ten studies assessed serum zinc or zinc deficiency rates.^11^, ^16^, ^22^, ^23^, ^24^, ^60^, ^61^, ^62^, ^63^, ^64^ There were 375 CD patients, 398 UC patients and 451 healthy controls.

Zinc is mostly intracellular and disease activity and CRP > 20 mg L^–1^ will limit interpretation in IBD.

#### Crohn's disease

One study in active CD showed no difference in serum zinc levels between patients (*n* = 22) and healthy controls (*n* = 11).^63^ Two studies showed lower serum zinc levels for patients in remission (*n* = 32)^22^ and patients with long standing CD in remission (*n* = 32) compared to healthy controls (*n* = 64).^23^ However newly diagnosed patients with CD (*n* = 20) in remission had similar zinc levels compared to healthy controls (*n* = 20).^23^ Two studies with mixed populations of active CD or CD in remission (*n* = 70) showed similar serum zinc levels compared to healthy controls (*n* = 146).^24^, ^61^

One study showed rates of zinc deficiency were similar between patients with CD in remission (4/94 [4.2%]) and no healthy controls (0%).^16^

#### Ulcerative colitis

Four studies in mixed populations of active UC or UC in remission assessed serum zinc levels. Two studies showed higher zinc in patients (*n* = 198) compared to healthy controls (*n* = 150),^61^, ^62^ one study found similar levels between groups (UC, *n* = 24; HC, *n* = 10)^64^ and one study found serum zinc levels lower in UC patients (*n* = 46) compared to healthy controls (*n* = 23).^24^

One study showed rates of zinc deficiency were similar between patients with UC in remission (1/50 [2.0%]) and no healthy controls (0%).^16^

#### IBD

Two studies did not separate patients with CD and patients with UC. Both showed serum zinc levels were significantly higher in patients compared to healthy controls, one was carried out in active IBD^60^ and the other was in a mixed population of patients with active IBD or IBD in remission.^11^

### Copper

Four studies assessed serum copper levels.^22^, ^24^, ^61^, ^63^ There were 113 CD patients, 163 UC patients and 200 healthy controls.

#### Crohn's disease

One study in active CD^63^ and one study in CD in remission^22^ showed no difference in serum copper levels between patients (*n* = 43) and healthy controls (*n* = 54). Two studies with mixed populations of active CD or CD in remission showed differing results with similar serum copper levels between patients (*n* = 23) and healthy controls (*n* = 23) in one study^24^ and higher serum copper levels in patients (*n* = 47) compared to healthy controls (*n* = 123) in the other study.^61^

No copper deficiency rates were reported.

#### Ulcerative colitis

Two studies with mixed populations of active UC or UC in remission showed differing results with similar serum copper levels between patients (*n* = 46) and healthy controls (*n* = 23) in one study^24^ and higher serum copper levels in patients (*n* = 117) compared to healthy controls (*n* = 123) in the other study.^61^

No copper deficiency rates were reported.

### Magnesium

Three studies assessed serum magnesium levels or magnesium deficiency rates.^16^, ^22^, ^24^ There were 149 CD patients, 96 UC patients and 126 healthy controls included in the analysis.

#### Crohn's disease

One study assessed serum magnesium levels in CD patients in remission and showed significantly lower levels of magnesium in all patients (*n* = 32) compared to healthy controls (*n* =  *n* = 32).^22^

One study in a mixed population of active CD and CD in remission showed similar magnesium levels between patients and healthy controls.^24^

Magnesium deficiency was 27 of 94 (28.7%) in patients with CD in remission and four of 61 (6.6%) in healthy controls (*p* > 0.05).^16^

#### Ulcerative colitis

One study assessed serum magnesium levels in a mixed population of active UC and UC in remission and showed significantly lower levels of magnesium in patients compared to healthy controls.^24^

Magnesium deficiency was 11 of 50 (22.0%) in patients with UC in remission and 6.6% in healthy controls. This was significantly different to healthy controls for female patients (*p* = 0.033) but not male patients (*p* > 0.05).^16^

### Selenium

Eight studies assessed serum selenium levels or selenium deficiency rates.^11^, ^16^, ^22^, ^24^, ^61^, ^64^, ^65^, ^66^ There were 684 CD patients, 328 UC patients and 1214 healthy controls.

#### Crohn's disease

One study in CD in remission showed lower selenium levels for patients (*n* = 32) compared to healthy controls (*n* = 32).^22^ Four studies included mixed populations of patients with active CD or CD in remission, three of these showed lower selenium levels for patients (*n* = 435) compared to healthy controls (*n* = 1013)^61^, ^65^, ^66^ and one study showed no differences between groups (patients, *n* = 23; HC, *n* = 23).^24^

One study showed high rates of selenium deficiency in patients with CD in remission (58/94 [61.7%]) and healthy controls (19/61 [31.1%]) with a significant difference between groups for males (*p* < 0.001) but not females (*p* > 0.05).^16^

#### Ulcerative colitis

Three studies assessed serum selenium levels in mixed populations of patients with active UC or UC in remission. Two studies showed significantly lower selenium levels in patients (*n* = 46) compared to healthy controls (*n* = 23)^24^, ^64^ and one study showed similar levels between groups (patients, *n* = 117; HC, *n* = 123).^61^

One study showed no difference in rates of selenium deficiency in patients with CD in remission (20/50 [40.0%]) and healthy controls (19/61 [31.1%]) (*p* > 0.05).^16^

#### IBD

One study in patients with IBD failed to show any difference in serum selenium levels for comparisons between active IBD or IBD in remission (*n* = 167) and healthy controls (*n* = 45).^11^

### Iron

**Practice Statement**

Iron is an acute phase reactant therefore interpretation of blood test results should consider inflammatory status. Anaemia of chronic disease often co‐exists with iron deficiency anaemia in inflammatory bowel disease. Agreement 94.4%.

Use serum ferritin, CRP, transferrin saturation and serum iron to assess presence of iron deficiency and haemoglobin, blood count and other micronutrients when diagnosing iron deficiency anaemia.

Three studies assessed serum iron levels.^29^, ^64^, ^67^ The analysis included 191 patients with CD, 287 patients with UC and 280 healthy controls. No studies assessed iron deficiency rates in IBD compared to healthy controls.

#### Crohn's disease

Two studies in patients with CD showed significantly lower iron levels for active disease (*n* = 110)^29^, ^67^ and remission^67^ compared to healthy controls (*n* = 135).

#### Ulcerative colitis

Three studies in active UC show serum iron levels are significantly lower in patients (*n* = 162) compared to healthy controls (*n* = 145).^29^, ^64^, ^67^ In UC in remission results are inconsistent with one study showing iron levels are significantly lower in UC (*n* = 8) than in healthy controls (*n* = 10)^64^ and the other showing similar levels between groups (UC, *n* = 93; HC, *n* = 105).^67^

## NUTRITIONAL ASSESSMENT: ENERGY AND MACRONUTRIENTS

**Practice Statement**

Dietary intake in inflammatory bowel disease (IBD) may be affected by differing food choices during periods of disease activity and remission and is an essential part of nutritional assessment in inflammatory bowel disease. Agreement 100%.

Suboptimal dietary intake is one of the contributing factors to reduced nutritional status in IBD. Estimates of dietary intake using a food diary, diet history or 24‐h recall are achievable in clinical practice. Dietary restriction is common in patients with active disease and in remission and is likely to affect diet quality.^68^ Probing questions regarding dietary avoidances enable an appreciation of dietary restrictions that may be associated with deficits in nutritional intake. Dietary intakes of energy, macronutrients and calcium and iron intake were assessed.

**Practice Statement**

Energy and macronutrient intake may be altered in inflammatory bowel disease and should be included as part of a nutritional assessment. Agreement 92.2%.

There were 15 studies that assessed energy intake^12^, ^18^, ^19^, ^21^, ^22^, ^23^, ^24^, ^25^, ^31^, ^32^, ^34^, ^69^, ^70^, ^71^, ^72^ with 856 patients with CD, 443 patients with UC and 2554 healthy controls. All studies included patients with CD and seven studies included patients with UC. Active IBD and IBD in remission were included in eight studies and seven studies included patients only in remission.

### Energy

Similar energy intakes compared to healthy controls (*n* = 1940) were observed in eight of 15 studies in CD patients (*n* = 572)^12^, ^21^, ^22^, ^23^, ^24^, ^31^, ^69^, ^72^ and four of seven studies in UC patients (*n* = 258) compared to 1607 healthy controls.^24^, ^31^, ^34^, ^72^ Lower energy intakes compared to healthy controls (*n* = 767) were found in six of 14 studies in CD patients (*n* = 381)^18^, ^19^, ^25^, ^32^, ^70^, ^71^ and three of six studies in UC patients (*n* = 167) compared to healthy controls (*n* = 527).^25^, ^32^, ^70^ One study found that CD patients (*n* = 20) had significantly higher energy intake compared to healthy controls (*n* = 31) but this study had a small sample size and these patients also had significantly higher protein and carbohydrate intakes.^34^

### Protein

Fourteen studies assessed protein intake^12^, ^18^, ^19^, ^21^, ^22^, ^23^, ^24^, ^25^, ^31^, ^34^, ^69^, ^70^, ^71^, ^72^ with 725 patients with CD, 359 patients with UC and 2474 healthy controls.

Eight of studies showed there was no difference in protein intakes between patients with CD (*n* = 539) and healthy controls (*n* = 487),^19^, ^21^, ^22^, ^23^, ^24^, ^69^, ^71^ and patients with UC (*n* = 161)^24^ and healthy controls (*n* = 107). Four studies showed significantly lower intakes for patients with CD (*n* = 187) compared to healthy controls (*n* = 547),^12^, ^18^, ^25^, ^70^ two studies showed significantly lower intakes for UC patients (*n* = 101) compared to healthy controls (*n* = 427)^25^, ^70^ and two studies showed a higher percentage of total energy from protein for CD (*n* = 111) and UC (*n* = 97) patients compared to healthy controls (*n* = 1500).^31^, ^34^

### Fat

There were 15 studies that assessed fat intake^12^, ^18^, ^19^, ^21^, ^22^, ^23^, ^24^, ^28^, ^31^, ^32^, ^34^, ^69^, ^70^, ^71^, ^72^ with 849 patients with CD, 400 patients with UC and 2525 healthy controls. Fourteen studies included patients with CD and seven studies included patients with UC. Active IBD and IBD in remission were included in eight studies and seven studies included patients only in remission.

Fat intake was significantly lower in CD patients (*n* = 154) compared to healthy controls (*n* = 1560) in three of 13 studies^19^, ^31^, ^34^ and in three of six studies in UC patients (*n* = 109) compared to healthy controls (*n* = 1512).^28^, ^31^, ^34^

Fat intake was significantly higher in CD patients (*n* = 121) compared to healthy controls (*n* = 486) in two of 13 studies^32^, ^70^ and in one of six studies in UC patients (*n* = 66) compared to healthy controls (*n* = 100).^32^

Fat intake was similar between CD patients (*n* = 607) and healthy controls (*n* = 515) in nine of 14 studies^12^, ^18^, ^21^, ^22^, ^23^, ^24^, ^69^, ^71^, ^72^ and three of seven studies in UC patients (*n* = 175) compared to healthy controls (*n* = 493).^24^, ^70^, ^72^

### Carbohydrate

There were 15 studies that assessed carbohydrate intake^12^, ^18^, ^19^, ^21^, ^22^, ^23^, ^24^, ^28^, ^31^, ^32^, ^34^, ^69^, ^70^, ^71^, ^72^ with 849 patients with CD, 400 patients with UC and 2525 healthy controls. Fourteen studies included patients with CD and seven studies included patients with UC. Active IBD and IBD in remission were included in eight studies and seven studies included patients only in remission.

There were no differences in carbohydrate intake between CD (*n* = 483) and healthy controls (*n* = 806) in nine of 14 studies^12^, ^18^, ^19^, ^21^, ^22^, ^32^, ^69^, ^70^, ^72^ and UC (*n* = 227) and healthy controls (*n* = 207) in three of seven studies.^24^, ^32^, ^72^ Compared with healthy controls (*n* = 1643), higher carbohydrate intake was found in five of 13 studies for CD patients (*n* = 261)^23^, ^24^, ^31^, ^34^, ^71^ and four of six studies for UC patients (*n* = 155) compared to healthy controls (*n* = 1898).^28^, ^31^, ^34^, ^70^

### Fibre

**Statement**

Fibre intake is likely to be low in inflammatory bowel disease and should be included as part of a nutritional assessment. (GRADE very low quality) agreement 88.1%.

Seven studies assessed fibre intake, three had a low risk of bias^22^, ^24^, ^32^ and four had unclear risk of bias ^31^, ^70^, ^71^, ^72^ in 471 CD patients, 226 UC patients and 2142 healthy controls.

Fibre intake was significantly lower in patients with CD (*n* = 439) compared to healthy controls (*n* = 2142) in six of seven studies^22^, ^31^, ^32^, ^70^, ^71^, ^72^ and patients with UC (*n* = 341) compared to healthy controls (*n* = 2062) in four of five studies.^31^, ^32^, ^70^, ^72^

### Calcium

**Statement**

Calcium intake should be assessed in Crohn's disease and ulcerative colitis because patients may not meet their recommended intake. (GRADE very low quality) agreement 96.3%.

Ten studies assessed calcium intake, three had a low risk of bias,^22^, ^24^, ^53^ four had an unclear risk of bias^71^, ^72^, ^73^, ^74^ and three had high risk of bias^25^, ^28^, ^75^ in 906 IBD patients (CD, *n* = 425; UC, *n* = 329; unknown, *n* = 152) and 837 healthy controls.

Four studies included patients in remission^22^, ^53^, ^71^, ^73^ and six studies included patients with active disease or disease in remission.^24^, ^25^, ^28^, ^72^, ^74^, ^75^

Six of nine studies found no significant difference in calcium intake between CD (*n* = 381) and healthy controls (*n* = 708)^22^, ^24^, ^25^, ^72^, ^73^, ^74^ and four of six studies between UC (*n* = 278) and healthy controls (*n* = 557).^25^, ^28^, ^72^, ^74^ Two studies found lower intakes compared to healthy controls, one in UC (*n* = 46) compared to healthy controls (*n* = 23)^24^ and one in mixed IBD (*n* = 152) compared to healthy controls (*n* = 73).^75^

## Iron

**Statement**

Iron intake should be assessed in Crohn's disease and ulcerative colitis as patients may not meet their recommended intake. (GRADE very low quality) agreement 96.2%.

Eight studies assessed dietary iron intake with three showing low risk of bias,^22^, ^24^, ^69^ three showing unclear risk of bias^21^, ^72^, ^73^ and two showing high risk of bias^25^, ^28^ in 348 patients with CD, 233 patients with UC and 336 healthy controls.

Three out of seven studies showed that iron intake was lower in CD (*n* = 215) compared to healthy controls (*n* = 216)^25^, ^69^, ^72^ and two of five studies showed that iron intake was lower in UC (*n* = 170) compared to healthy controls (*n* = 125).^25^, ^72^ All other studies (4/7) showed no differences in iron intake.^21^, ^22^, ^24^, ^28^, ^73^ One study reported that only 32% of patients with CD met the recommended intake for iron.^69^

## NUTRITIONAL SCREENING

**Practice Statement**

Screening for nutritional risk may be considered an integral part of inflammatory bowel disease (IBD) care in the IBD multidisciplinary care setting. Agreement 98.2%.

BMI is not an accurate marker of malnutrition in IBD patients and reduced lean body mass may be missed in patients with a normal or high BMI. In addition, dietary exclusion to manage symptoms is common in IBD and avoidance of specific foods and/or food groups increases the risk of micronutrient deficiencies. Thus, nutrition screening tools that rely heavily on BMI may not be capturing some patients at nutritional risk.

**Statement**

MUST may be used to screen patients with inflammatory bowel disease for risk of malnutrition GRADE very low quality. Agreement 85.5%.

Ten studies assessed malnutrition risk using the Malnutrition Universal Screening Tool (MUST), four were unclear risk of bias^76^, ^77^, ^78^, ^79^ and six were high risk of bias.^80^, ^81^, ^82^, ^83^, ^84^, ^85^ These studies included 80 inpatients (CD, *n* = 62; UC, *n* = 18)^76^, ^82^, ^83^, ^84^ and 936 outpatients (CD, *n* = 582; UC, *n* = 347; unclassified IBD, *n* = 7).^77^, ^80^, ^81^, ^84^

Combining the results from all ten studies, MUST scores for all IBD patients were 751/1080 (70%) for low risk, 128/1080 (12%) for moderate risk and 198/1080 (18%) for high risk. For outpatients, MUST scores were 734/998 (74%) for low risk, 112/998 (11%) for moderate risk and 151/998 (15%) for high risk. For inpatients, MUST scores were 17/80 (21%) for low risk, 16/80 (20%) for moderate risk and 47/80 (59%) for high risk. The MUST tool assesses risk of malnutrition and the other tools assess nutrition risk.^78^, ^79^, ^86^

The Saskatchewan IBD nutrition risk tool (SaskIBD‐NR) uses a score based on symptoms, weight loss and food restriction. In 110 IBD outpatients (CD, *n* = 75; UC, *n* = 35), it showed significant agreement with a registered dietitian or gastroenterologist assessment (*k* = 0.83, *p* < 0.001), whereas MUST showed a lack of agreement (*k* = 0.15, *p* = 0.12).^79^ The SaskIBD‐NR had better sensitivity (82.6% vs. 26.1%), specificity (97.7% vs. 87.4%), positive predictive value (90.5% vs. 35.3%) and negative predictive value (95.5% vs. 81.7%) than MUST. In patients with a BMI > 25.0 kg m^–2^, the SaskIBD‐NR had better sensitivity (63.6% vs. 27.3%) but not specificity (73.2% vs. 90.2%) than MUST to identify patients malnourished using Subjective Global Assessment (SGA).^87^ In patients waiting for surgery,^85^ SaskIBD‐NR and MUST performed similarly and did not identify all patients malnourished according to the Global Leadership Initiative on Malnutrition (GLIM) criteria but did identify a similar percentage of patients malnourished according to ESPEN criteria.

Malnutrition inflammatory risk tool (MIRT) includes BMI, unintentional weight loss and CRP. In a study of 55 patients with CD, seven (12.7%) were mild to severely malnourished (SGA B or C) and nine (16.4%) had a MIRT score ≥ 3 which was associated with worse clinical outcomes at 6 months.^86^ The number of malnourished patients according to SGA reduced from seven to three over the 6‐month period and it is not reported whether MIRT identified all malnourished patients.^86^ In a surgical IBD cohort, MIRT successfully identified malnourished patients using GLIM criteria.^85^

The Inflammatory Bowel Disease‐Nutrition Screening tool (IBD‐NST) uses a score based on BMI, weight loss, active disease and nutrition concerns.^78^ In 101 IBD outpatients (CD, *n* = 61; UC, *n* = 33; IBDU, *n* = 7), 12 patients who were low risk for MUST were high risk for IBD‐NST as a result of having a flare of IBD symptoms and concerns about their nutrition. IBD‐NST was compared with SGA, MUST, hand grip strength, mid‐arm muscle circumference and BMI. Unlike SGA and MUST, IBD‐NST nutrition risk was not predicted by BMI (area under the curve = 0.262 [SE = 0.06]; 95% confidence interval [CI] = 0.17–0.40).

There are now multiple screening tools available. The best screening tool for clinical practice will depend on the purpose of nutrition screening and the capacity of the health service to deliver appropriate nutrition interventions.^86^

### Self‐screening

Four recent studies from Canada^81^, ^87^ and the UK^77^, ^78^ have found that patients can accurately self‐screen for risk of malnutrition using MUST^77^, ^78^, ^81^, ^87^ IBD‐NST,^78^ SaskIBD‐NR^87^ or abridged patient‐generated SGA.^87^ The use of self‐screening may be a more cost‐effective method of identifying patients who are likely to benefit from further nutritional assessment and dietetic input.

## ORAL NUTRITIONAL SUPPORT

**Practice Statement**

Oral nutritional supplementation may improve nutritional status in patients with inflammatory bowel disease. Agreement 88.9%.

Four studies met the inclusion criteria.^76^, ^88^, ^89^, ^90^ Three studies only included 91 CD patients,^76^, ^88^, ^89^ and one study included 13 CD and 25 UC patients.^90^

All studies used different oral nutritional supplementation products for different durations.

One study was a crossover study in outpatients with active CD or CD in remission. Patients received normal diet for 2 months or normal diet supplemented with oral nutritional supplement (ONS) (Ensure Plus; Abbott) for 2 months or vice versa. Both groups had an increase in energy intakes, weight and mid‐arm muscle circumference (*p* < 0.05). Comparisons between groups were not presented.^88^

One study allocated patients with CD in remission to oral nutritional supplements (Elemental 028; Scientific Hospital Supplies) (*n* = 21) or a normal diet (*n* = 18) for 12 months.^89^ Weight and BMI increased and more patients remained in remission at 12 months in the ONS group compared to the normal diet group.

One study in 25 inpatients with active CD gave ONS (Modulen IBD; Nestle) for 3 months to eight patients and dietary counselling and normal hospital diet to 17 patients. In the ONS group, weight, BMI, MUST, SGA and handgrip strength all improved after 3 months, whereas, in the dietary counselling and normal hospital diet group, only handgrip strength improved.^76^

One study in active IBD (UC, *n* = 25 UC; CD, *n* = 13) randomised patients to receive ONS (Novasource; Nestle) in addition to hospital diet or hospital diet for 3 weeks. Patients who refused ONS were transferred to the hospital diet group. SGA, protein and energy intake and disease activity scores improved in both groups. No comparisons between groups were presented.^90^

Despite limited evidence in IBD, that ONS improves nutritional status, dietary advice with or without ONS may improve nutritional status in adults with malnutrition.^91^

## INDUCTION OF REMISSION

### Enteral nutrition

**Statement**

To induce remission in active Crohn's disease, exclusive enteral nutrition (EEN) is less effective than corticosteroids. EEN may be used in mild to moderate disease where avoidance of corticosteroids is intended, and dietetic expertise is available. (GRADE very low quality) agreement 84.6%.

A recent systematic literature review of exclusive enteral nutrition (EEN) to induce disease remission in adults with Crohn's disease reviewed literature published from inception to July 2017.^92^ No further studies in adults were identified after this date. Six RCT published from 1985 to 2002 were included in an intention to treat and per protocol meta‐analysis of EEN versus corticosteroids to induce remission.^93^, ^94^, ^95^, ^96^, ^97^, ^98^ The intention to treat meta‐analysis found 87 of 194 (45%) patients on EEN and 116 of 158 (73%) patients on corticosteroids achieved disease remission with risk ratio of 0.65 (95% CI = 0.52–0.82) favouring corticosteroids. The per protocol meta‐analysis induction of remission results also favoured corticosteroids with 87 of 149 (58%) patients on EEN and 116 of 158 (73%) on corticosteroids with a risk ratio of 0.82 (95% CI = 0.70–0.95). These RCTs were reported between 1985 and 2002. The palatability of and methods used to administer current enteral nutrition formula have significantly improved since then. In addition, evidence from recent paediatric RCTs suggest that EEN is equivalent to corticosteroids at inducing disease remission.^92^, ^99^, ^100^ Recent guidelines suggest that EEN may be considered as an alternative to corticosteroids in patients with mildly active CD.^47^, ^101^

**Practice Statement**

In steroid‐refractory or steroid‐intolerant disease, exclusive enteral nutrition may be used as an adjuvant to induce disease remission in patients with active Crohn's disease starting immunosuppressive and/or biologic therapy. Agreement 84.3%.

Two case series reported improvement in CRP and disease activity in patients with active Crohn's disease treated with exclusive enteral nutrition as an adjuvant to aminosalicylates, immunosuppressive and/or biologic therapy.^102^, ^103^ One case series in 16 adults with active Crohn's disease treated with exclusive enteral nutrition as an adjuvant to existing Crohn's disease medications (details not provided)^102^ showed significant reductions in Harvey Bradshaw index (6.5 ± 5.8 to 2.4 ± 3.3) (*p* = 0.001) and CRP (3.5–0.88 mg dl^–1^) (*p* = 0.023). Another case series in 25 patients with active Crohn's disease treated with EEN included 13 patients on stable doses of mesalazine (*n* = 9), immunosuppressants (*n* = 3) or biologic (*n* = 1) medications. Only 14 of 25 (56%) patients completed EEN and achieved disease remission (Harvey Bradshaw Index < 5).^103^ The outcomes of patients on concurrent medications were not reported separately. The two case series suggest that remission may be induced in patients with active Crohn's disease treated with EEN in addition to concurrent medications but this needs to be confirmed in controlled studies. The use of exclusive enteral nutrition to induce disease remission in patients with active disease on an optimised regimen of immunosuppressant and/or biologic medication has not been described in the literature.

A recent open label RCT of 7 days of EEN in acute severe UC has investigated whether EEN can improve the response to intravenous corticosteroids.^104^ On an intention‐to‐treat analysis, corticosteroid treatment failed in eight of 32 (25%) patients who received EEN versus 13 of 30 (43%) who ate a normal diet (*p* = 0.051). Larger high‐quality studies are warranted to confirm whether EEN can augment the effectiveness of corticosteroids in acute severe UC.

**Practice Statement**

Exclusive enteral nutrition, either used as a primary or adjuvant therapy, may be used for a minimum of 6 weeks to induce disease remission and achieve mucosal healing in patients with mild to moderate Crohn's disease in which corticosteroids are contradicted or the patient chooses to avoid corticosteroids. Agreement 84.3%.

Access to dietetic expertise and use of a defined pathway are essential for successful use of exclusive enteral nutrition.^47^, ^105^ Practical guidance is provided in Table 6.

**Table 6:** Practical considerations for using exclusive enteral nutrition (EEN) in Crohn's disease

For successful EEN, ensure the whole multi–disciplinary team support its use Some patients may choose EEN rather than pharmaceutical management Corticosteroids may be contra–indicated in some patients and EEN is a suitable alternative to induce disease remission EEN can be used as an adjunct to other pharmaceutical management options Always provide patients with detailed information on how to implement EEN in a structured manner Calculate nutritional requirements based on resting energy expenditure (25–30 kcal kg^–1^ day^–1^)^106^ and protein (1 g kg day^–1^)^107^ Be prepared to modify nutritional requirements depending on physical activity level especially if patients report being hungry once EEN is established Provide different flavours and product samples of EEN, some will have different flavour options Use a starter regimen to commence EEN and where applicable gradually increase EEN over 3 days as food is reduced and stopped EEN can often be commenced in the out–patient setting and most patients can continue with their usual day‐to‐day activities Where hospital admission is necessary, consider refeeding risk and monitor urea and electrolytes, phosphate and magnesium daily until energy requirements are established Ensure EEN prescription is approved by the primary care team at the recommended dose and duration Once the target regimen is met, EEN can induce clinical remission within 10 days, however mucosal healing takes 6 weeks. Discuss the appropriate duration with the patient and multi–disciplinary team The majority of patients can tolerate EEN orally, however if patients are struggling to meet target volumes consider nasogastric feeding Regular support from a dietitian is vital to achieve and maintain patient motivation with EEN for the whole duration Ensure dietetic support is available to troubleshoot issues with tolerance, adherence, dental care, stool colour and symptoms. Consider adjusting EEN volume and the concentration of a powder feed Once EEN is established, consider issues around motivation to continue for the whole EEN duration and discuss other food intake, social interaction, peer pressure and hunger EEN protocols will normally allow some other fluids (e.g., water, weak black tea and coffee or diluted squash) Provide information on how to reintroduce food after a period of EEN and where appropriate gradually reducing the volume of enteral nutrition as more food is introduced

### Whole food or elimination diets

**Practice Statement**

There is insufficient evidence to support the use of a whole food diet to induce disease remission in adults with active inflammatory bowel disease. Agreement 87.5%

Practical dietary advice for IBD is provided in Table 7. Ten whole food dietary exclusion interventions to induce remission in adults with active IBD were reviewed. These are anti‐inflammatory IBD diet, low FODMAP (i.e., fermentable oligosaccharides, disaccharides, monosaccharides and polyols) diet, Crohn's disease exclusion diet (CDED), IgG4, Western diet, low microparticle diet, semi‐vegetarian plant‐based diet, Mediterranean diet, specific carbohydrate diet (SCD) and Crohn's disease TReatment with EATing diet (CD‐TREAT).^109^, ^110^, ^111^, ^112^ The results of a feasibility study of a low emulsifier diet in adults with active IBD are also promising.^113^ However, high quality evidence is needed for most of these whole food interventions before they may be recommended as alternative treatments to current management strategies recommended as primary treatment for active disease.

**Table 7:** Practical dietary advice for inflammatory bowel disease (IBD)

Discourage self‐directed non‐evidence‐based exclusion diets as they can promote nutritional deficiency and low diet quality Many dietary components have been associated with IBD, however, there is no evidence to support restriction of any one dietary component
For stable inflammatory bowel disease
Include a wide range of fruit, vegetables, nuts and seeds and wholegrains (with exception of stricturing disease) to meet energy and nutritional requirements. There is no need to restrict fibre intake, except in stricturing disease (see below) Include a variety of protein–rich foods, with a reduction in animal fat and processed meat. Limit red and processed meat to no more than twice weekly (approximately 150 g week^–1^) to reduce risk of colon cancer^108^ Limit intake of high fat, high sugar and high salt foods – guidance on label reading may be useful Consider vitamin D supplementation throughout the year especially if vitamin D deficient
Stricturing inflammatory bowel disease
Stricturing disease may need an individualised approach with (i) avoidance of fibrous foods (i.e. tough outer skins and stalks of fruit and vegetables, tough/grisly meat) and (ii) inclusion of foods rich in soluble fibres accompanied by fluids consumed at the same time
Steroids
Steroids reduce calcium absorption and can lead to bone resorption, supplement patients on steroids with vitamin D and calcium, do not continue long term unless calcium intake is below 800 mg day^–1^. Calcium without vitamin D may increase cardiovascular risk.^47^

A 2019 Cochrane review concluded that there is insufficient evidence to determine whether exclusion diets induce disease remission in Crohn's disease or ulcerative colitis.^114^ Some of their reviewed studies did not meet our inclusion criteria.^115^, ^116^, ^117^ Subsequent to this review, new evidence from RCTs in adults with IBD has been published. Below is a summary of the evidence for each whole food dietary exclusion intervention.

#### CDED

The CDED is phased dietary approach comprised of an initial 6 weeks of partial enteral nutrition (50% of energy requirements) and an exclusion diet (50% of energy requirements) followed by a second 6 weeks of partial enteral nutrition (25% of energy requirements) with a slightly less restricted diet.^118^ It was first described in 2014 with a case series of 13 adults (six males, seven females) aged greater than 18 years. Subsequently, a RCT in paediatric Crohn's disease compared CDED with EEN and found that the CDED was as effective as EEN to induce disease remission^111^ and a pilot RCT in adults with mild‐to‐moderate Crohn's disease was published in 2022.^112^ The RCT in adults compared the CDED without partial enteral nutrition to the CDED with partial enteral nutrition. The pilot study found that, after 6 weeks of treatment, 57% of the CDED alone versus 68% (*p* = 0.462) of the CDED with partial enteral nutrition group were in clinical remission.^112^

#### CD‐TREAT

The CD‐TREAT diet is a personalised diet designed to replicate the gut microbiome changes that have been observed in patients treated with EEN.^109^ It contains a moderate amount of complex carbohydrate, higher amounts of protein, is low in fibre and includes an oral multivitamin tablet. A non‐randomised study in five children showed that, after 8 weeks of the dietary treatment, disease activity and faecal calprotectin reduced significantly.^109^ An open‐label trial in 32 adults with mild‐to‐moderately active Crohn's disease has been conducted. The trial showed that 23 of 32 adults completed the 8‐week treatment and patients who strictly adhered to the treatment had a significant reduction in faecal calprotectin and 80% achieved clinical disease remission.^110^

#### Low FODMAP diet^119^

The low FODMAP diet is a phased dietary approach that initially restricts the intake of short‐chain fermentable carbohydrates. One RCT compared the low FODMAP diet to a normal diet in patients with mild‐moderately active IBD (*n* = 13) or IBD in remission (*n* = 65) with co‐existing functional bowel symptoms.^120^ The low FODMAP diet had no significant effect on disease activity compared with the normal diet group for simple clinical colitis activity index or Harvey Bradshaw index. Research supports a low FODMAP diet to manage functional bowel symptoms rather than induce disease remission for active IBD.^120^, ^121^

#### Anti‐inflammatory diet

The anti‐inflammatory IBD diet is a phased dietary approach that includes modification of dietary carbohydrates, increased foods with pre‐ or probiotic properties, intake of anti‐inflammatory fats and modification of eating pattern or dietary texture. The use of the anti‐inflammatory IBD diet, in addition to usual IBD medications, has been reported in an uncontrolled, retrospective case series of 40 IBD patients.^119^ The case series reported that 4 weeks of the strict dietary regimen improved disease activity in 10 of 11 patients who had a complete data set and 24 of 40 (60%) self‐reported a good or very good response to the diet.

#### SCD and Mediterranean diet

The SCD is a phased dietary treatment that includes carbohydrate rich foods that contain only monosaccharides, excludes foods rich in disaccharides (e.g., sucrose and lactose) and polysaccharides (e.g., grains, starchy vegetables and specific legumes). The diet includes unprocessed protein‐rich foods and excludes most processed foods. Use of the SCD as a treatment for active IBD has been described in various paediatric case series. There has been one randomised superiority trial comparing 12 weeks of the SCD to a Mediterranean diet in adults with mild‐to‐moderately active Crohn's disease (*n* = 194).^122^ Participants received all their meals and snacks for the first 6 weeks. At baseline, faecal calprotectin >250 µg g^–1^ and high‐sensitivity CRP > 5  mg L^–1^ were reported in 38% and 67% of participants, respectively. At week 6, less than half the participants (46.5% on SCD and 43.5% on Mediterranean diet) were in remission using the short Crohn's disease activity index, less than 35% of participants had a faecal calprotectin <250 µg g^–1^ and less than 5% had high‐sensitivity CRP < 5 mg L^–1^. The SCD was not found to be superior to the Mediterranean diet and neither diet resulted in normalised serum CRP concentration.^122^

#### IgG4‐guided exclusion diet^111^, ^118^

The IgG4‐guided exclusion diet is based on the premise that improvement in functional gut symptoms have been shown after the individualised exclusion of foods based on IgG4 reactivity. A multi‐centre, double‐blind, randomised, controlled trial of the IgG4‐guided exclusion diet was conducted in 98 adults with active Crohn's disease.^123^ Patients either received dietary advice on the guided exclusion diet or a sham diet for 4 weeks, and 70 patients were taking immunomodulatory medication. In the intention to treat analysis, significant improvements in Crohn's disease activity index (CDAI) were observed in patients taking the exclusion diet (mean change 55.7; interquartile range [IQR] = −113 to 216) compared with patients taking the sham diet (mean change 16.8; IQR = −196 to 196). However the results do not report how many patients achieved disease remission (CDAI <150). Faecal calprotectin and CRP were not included in the intention to treat analysis and the per protocol analysis in 76 patients who competed the study showed there were no significant differences in change of faecal calprotectin (0) (*p* = 0.19) or CRP (47) (*p* = 0.13) between groups. The use of IgG4 exclusion diet in patients with active Crohn's disease reduced disease activity symptoms but did not result in significantly greater improvements in markers of inflammation than a sham diet.

#### Organic pre‐Western diet

The Organic pre‐Western diet hypothesis is that dietary intake in Western industrialised countries has changed substantially since the start of the 20th century and that exclusion of foods and practices associated with industrialisation will promote intestinal healing in mild to moderately active Crohn's disease. This theory was tested in a RCT of 18 patients with active Crohn's disease (CDAI = 150–220) and ulceration of the small or large bowel visible on magnetic resonance imaging (MRI). Concomitant use of a stable dose of medical therapy was permitted.^124^ The dietary intervention involved following an organically produced diet of red meat, spelt sourdough, and small amounts of fresh butter, rape oil, tea, tap water and rock salt. Fruit and vegetables were excluded as a result of the inability to ensure that they were always organically produced. Patients were also instructed to use baking soda toothpaste, avoid using a dishwasher and rinse plates in water after washing by hand. The control group were advised to eat a low‐fat, high carbohydrate diet, with avoidance of fibre‐rich fruit and vegetables and red meat. Both groups received vitamin B complex and vitamin C intramuscular injections every 3 weeks. Eight patients were randomised to the intervention (three withdrew at baseline after receiving dietary education) and 10 were randomised to the control diet (one withdrew after receiving dietary education). After 6 weeks of dietary treatment, MRI improvements were observed in three of eight (38%) of the intervention group and one of 10 (10%) of the control group and remission, defined as CDAI < 150, was achieved by four of eight (50%) patients in intervention group and seven of 10 (70%) of patients in control group. An RCT in 213 CD patients in remission (CDAI < 150) showed no difference in the rate of disease flare ups between a diet low in red meat with less than one serving of red or processed meat per month compared to a diet with more than two servings of red or processed meat per week.^125^ The use of this restrictive diet, which required intramuscular vitamin supplementation to meet nutritional requirements, requires further scientific evidence to determine efficacy.

#### Low microparticle diet

A low microparticle diet to induce CD remission was investigated in a RCT.^126^ Microparticles are not only present in the environment, but also are increasing in the food supply as food additives. A pilot RCT of a low microparticle diet in patients taking corticosteroids showed that the diet, in conjunction with a low calcium intake, may result in disease remission occurring more quickly compared with a normal diet.^127^ A subsequent multicentre RCT was conducted where 83 patients were randomised to one of four groups for 16 weeks: calcium controlled low mircoparticle diet with a placebo supplement or a 400 mg day^–1^ calcium supplement or a calcium controlled normal microparticle sham diet with a placebo supplement or a 400 mg day^–1^ calcium supplement. All patients started on 30 mg day^–1^ reducing dose of prednisolone and could take aminosalicylate (Pentasa) but no other CD medication. At week 16, there were no statistically significant differences in disease activity, CRP or faecal calprotectin between the low and normal microparticle diets or the normal and low calcium groups. The adjuvant use of low microparticle diet and/or a low calcium intake, in addition to a reducing dose of prednisolone, did not provide additional benefit over and above a normal microparticle diet.

#### Semi‐vegetarian plant‐based diet

The use of semi‐vegetarian plant‐based diet in conjunction with anti‐tumour necrosis factor α medication Infliximab to induce remission of active CD was investigated in a multi‐centre cohort study.^128^ Patients (35 adults [26 biologic naïve] and 11 children) received enteral nutrition for 3–7 days prior to the first infliximab infusion at week 0 and started on a lacto‐ovo‐semi‐vegetarian diet for 6 weeks, which included fish once a week and red meat once a fortnight. Infliximab infusions were also given at weeks 2 and 6. At baseline, seven of 35 (20%) were in remission (CDAI < 150) and, at week 6, remission was observed in 33 of 35 (94%) adults and CRP normalised in 28 of 35 (80%) adults. The major limitation of this study is the lack of a control group who received infliximab induction therapy without dietary intervention. RCT evidence of efficacy of the semi‐vegetarian plant‐based diet in conjunction with biological therapy is required before a clinical recommendation can be made.

### Fibre

**Practice Statement**

There is insufficient evidence to use prebiotic fibre to treat active ulcerative colitis. Agreement 87.0%.

Two systematic reviews of fibre (supplement or whole diet intervention) as a treatment to induce remission in IBD were performed.^129^, ^130^ Five RCTs were identified that included 114 patients with active UC.^131^, ^132^, ^133^, ^134^, ^135^ All studies assessed the effects of a prebiotic, two of which were combined with a probiotic compared with a placebo. Study duration varied from 2 weeks to 12 months and there was no homogeneity of outcomes to which GRADE criteria could be applied. Compared with placebo the findings from these studies are summarised below.

Germinated barley fibre (20–30 g day^–1^) led to lower overall disease score at 4 weeks (*p* = 0.045), mainly driven by reduced frequency of diarrhoea.^134^

Fructo‐oligosaccharide (12 g day^–1^) plus probiotic led to greater reduction in sigmoidoscopy score at 4 weeks but failed to reach significance (*p* = 0.06) and there was no difference in clinical disease activity score.^131^

Oligofructose inulin (12 g day^–1^) alongside mesalamine (3 g day^–1^) for 2 weeks had no effect on clinical outcomes compared to placebo alongside mesalamine (3 g day^–1^), although faecal calprotectin was lower than baseline in the prebiotic group only (*p* < 0.05).^132^

Galacto‐oligosaccharide (5.5 g day^–1^) with a probiotic led to similar endoscopic score at 1 year compared with placebo. However, there was a significant reduction in faecal myeloperoxidase for the intervention arm compared to placebo (*p* < 0.05).^133^

Oligosaccharide inulin (7 g day^–1^) with a probiotic and micronutrients for 2 months led to lower interleukin (IL)‐6 (*p* < 0.05), IL‐8 (*p* < 0.01) and lymphocyte expression (*p* < 0.05) but clinical outcomes were not reported.^135^

No further RCTs in UC patients have been identified subsequent to the 2014 systematic review of fibre by Wedlake et al.^129^

**Practice Statement**

There is insufficient evidence to use prebiotic fibre to treat active Crohn's disease. Prebiotic fibre may increase abdominal symptoms. Agreement 96.3%.

Three studies that measured the effect of prebiotic fibre on clinical outcomes in 156 patients with active CD ^124^, ^136^, ^137^ have been systematically reviewed.^124^, ^129^, ^136^, ^137^

An organic high‐fibre diet (*n* = 8; mean fibre content 46 g day^–1^) was compared with a control low‐fibre diet (*n* = 10; mean fibre content 16 g day^–1^).^124^ There was no difference between groups for remission rates and on an intention to treat basis, with three of eight patients achieving mucosal healing in the high fibre group compared to one of 10 in the control group.

Oligofructose inulin (12 g day^–1^) alongside a probiotic showed no difference in remission rates at 6 months in patients with active CD (*n* = 19) compared to placebo (*n* = 16); however, the treatment group had a significant improvement in histological scores from baseline but the control did not. There were no changes in either group in bowel habit, biochemical markers or IBD questionnaire scores.^137^

Oligofructose inulin (15 g day^–1^, *n* = 54) compared to placebo (*n* = 49) for 4 weeks in active CD patients demonstrated no improvement in clinical outcomes and worse flatulence and abdominal pain and rumbling after prebiotic, although lower numbers of IL‐6 and higher numbers of IL‐10 expressing dendritic cells in the prebiotic group were reported.^136^

No further randomised controlled trial were identified after the publication of the systematic review by Wedlake et al.^129^

### Vitamin D

Low serum vitamin D is common in IBD. Adjuvant high dose vitamin D supplementation improves serum vitamin D concentrations but there is conflicting evidence on the effect of vitamin D supplementation on disease activity.^138^, ^139^

Public Health England recommend that all adults should consider taking a daily vitamin D supplement of 10 μg from October to March to improve their vitamin D status.

### Fish oil

**Practice Statement**

There is insufficient evidence that a fish oil supplement reduces disease activity in active inflammatory bowel disease. Agreement 88.9%.

An RCT in patients with mild to moderately active UC (*n* = 121) found that partial enteral nutrition (33% of energy) supplemented with fish oil (2.5 g day^–1^ eicosapentaenoic acid and 1 g day^–1^ docosahexaenoic acid) and fructo‐oligosaccharide (6.5 g day^–1^) had no effect on clinical outcomes or histology after 6 months compared TO a carbohydrate‐based placebo.^140^ At baseline, 50% of patients were taking prednisone and/or sulfasalazine and medication doses could be adjusted based on patient symptoms. The effect of the fish oil could not be extrapolated from the mixed enteral nutrition and drug therapy; however, there were no differences between the intervention and placebo in reduction in disease activity.

An open label study in mild to moderately active CD (*n* = 20) using partial enteral nutrition (33% of energy) supplemented with fish oil (2.5 g day^–1^ eicosapentaenoic acid and 1 g day^–1^ docosahexaenoic acid) and fructo‐oligosaccharide (6.5 g day^–1^) was conducted.^141^ At baseline, 50% of patients were taking corticosteroids. A number of adverse effects were attributed to the intervention in active CD including nausea, excessive flatulence, faecal incontinence, hot flushes and abdominal pain, although it would be difficult to determine the ingredient that induced the effects. Half of the patients achieved 85% compliance with the nutrition intervention associated with a >2% increase in plasma phospholipid eicosapentaenoic acid (*n* = 10). This group showed a significant improvement in CDAI change (−47.8 [−65 to −37]) (*p* = 0.049), whereas patients with 54% compliance (*n* = 10) had no change in CDAI (−8.1 [−54.6 to 40.1]) (*p* = 0.99). At 4 months, CDAI was significantly less for the 85% compliance patients (116 ± 95) compared with the 54% compliance patients (262 ± 87) (*p* < 0.005). The number of patients who achieved disease remission was not reported. These results need to be interpreted with caution as a result of the study being open label with small numbers, the use of corticosteroids and the inability to extrapolate the effect of the fish oil from the enteral nutrition.

### Complementary and alternative medicine

**Practice Statement**

There is insufficient evidence to recommend the routine use of complementary or alternative medicine in inflammatory bowel disease. Agreement 81.5%.

A systematic review of complementary and alternative medicines to improve disease activity and markers of inflammation in IBD was published in 2015.^142^ The systematic review concluded that there was a lack of homogeneity in the literature and that complementary and alternatives therapies may be effective, although further research is required to confirm their efficacy. Subsequent to this systematic review, nine further RCTs to improve disease activity in active IBD have been published: seven using a curcumin supplement^143^, ^144^, ^145^, ^146^, ^147^, ^148^, ^149^ and two using cannabidiol‐rich botanical extract.^150^, ^151^ The evidence and recommendation for curcumin supplementation in active UC is described below. Two RCTs showed cannabidiol‐rich botanical extract versus placebo improved disease activity^150^, ^151^ but not endoscopic healing.^151^ Further details are available in recent reviews and guidelines.^47^, ^152^

#### Curcumin

**Practice Statement**

In patients with active ulcerative colitis, curcumin supplementation in addition to mesalamine may improve clinical response, however optimal dose, formulation and duration are unknown. Agreement 81.1%.

Six RCTs in 352 patients with mild to moderately active UC were identified. Five studies reported a benefit of curcumin supplementation compared to placebo on (i) disease activity (reported in five studies)^144^, ^145^, ^146^, ^147^, ^148^ and (ii) endoscopic score (reported in three studies).^146^, ^147^, ^148^, ^153^ One study did not show benefit.^143^ Curcumin was provided as an adjuvant therapy to mesalamine. Interventions were between 1 and 3 months and studies used different formulations (capsules, enema) and dose (50 mg to 3 g) of curcumin. Clinical response (fall of greater or equal to 3 points of the simple clinical colitis activity index) was reported in five studies and was observed in 85 of 175 (49%) of patients taking curcumin compared with 52 of 177 (29%) of patients on placebo. Endoscopic remission (Partial Mayo score of less than or equal to 1) was observed in 36 of 83 (43%) patients taking curcumin compared to 10 of 81 (12%) on placebo.

In one small RCT, 30 patients (2:1 ratio) with mild to moderately active CD were randomised to curcumin or placebo for 12 weeks; eight of 20 patients in the curcumin group achieved remission compared with zero of 10 patients in the placebo group.^149^

### Probiotics

Eight systematic reviews of probiotics in active IBD were reviewed for RCTs that met the inclusion criteria.^154^, ^155^, ^156^, ^157^, ^158^, ^159^, ^160^, ^161^ One further study also met the inclusion criteria.^162^

**Statement**

In some patients with mildly active ulcerative colitis, taking specific probiotics alongside usual medication may support induction of disease remission. (GRADE moderate quality) agreement 88.7%.

Ten RCTs assessed the effect of probiotics in active UC alongside usual medication, mostly 5‐aminosalicylic acid. One study had low risk of bias,^163^ five studies had unclear risk of bias ^162^, ^164^, ^165^, ^166^, ^167^ and four studies had high risk of bias.^168^, ^169^, ^170^, ^171^ The analysis included 356 patients randomised to a probiotic and 311 randomised to placebo. Different probiotics were used across studies. Seven studies showed more patients achieved remission with probiotics (124/272 [46%]) compared to placebo (75/277 [27%]). The probiotics were: De Simone Formulation in three studies^166^, ^167^, ^169^; Yakult *Bifidobacterium breve* strain Yakult, *Bifidobacterium bifidum* strain Yakult and a *Lactobacillus acidophillus* strain in one study^164^; *Bifidobacterium longum* 536 (BB‐536) in one study^170^; Profermin *Lactobacillus plantarum* 299v in one study ^168^; and *Lactobacillus casei* Zhang, *Lactobacillus. plantarum* P‐8 and *Bifidobacterium animalis* subsp. *lactis* V9 in one study.^162^ Three studies showed similar remission rates between probiotics (53/112 [47%]) and placebo (40/62 [65%]) with these probiotics: *Escherichia coli* nissle in two studies,^163^, ^165^ and *Lactobacillus. casei* strain ATCC PTA‑3945 in one study.^171^

In practice, if patients want to try a probiotic, they should be given the evidence highlighting which ones have been shown to be beneficial in ulcerative colitis (for practical guidance, see Table 8).

**Practice Statement**

There is no evidence for probiotics to induce disease remission in Crohn's disease. Agreement 81.1%.

**Table 8:** Practical guidance for probiotics

Disease and activity	Patients given probiotic (*n*)	Probiotic	References
Ulcerative colitis			
Induce remission	77	De Simone Formulation (vivomix)	Ng et al.,^166^ Tursi et al.,^167^ Sood et al.^169^
	14		
	65		
	10	Yakult *Bifidobacterium breve* strain Yakult, *Bifidobacterium bifidum* strain Yakult and a *Lactobacillus acidophillus*	Kato et al.^164^
	28	*Bifidobacterium longum* 536 (BB‐536)	Tamaki et al.^170^
	32	Profermin *Lactobacillus plantarum* 299v	Krag et al.^168^
	12	*Lactobacillus casei* Zhang, *Lactobacillus plantarum* P‐8 and *Bifidobacterium animalis* subsp. *lactis* V9	Chen et al.^162^
Remission maintenance	15	Bifid triple viable capsule (BIFICO)	Cui et al.^172^
Pouchitis			
Relapsing	76	De Simone Formulation (vivomix)	Gionchetti et al.,^173^ Gionchetti et al.,^174^ Mimura et al.^175^ Pronio et al.^176^
Remission	7	*Bifidobacterium longum* BB‐536	Brown et al.^177^
Crohn's disease			
Induce remission	10	*Saccharomyces boulardii*	Plein and Hotz^178^
Remission maintenance	–	No studies	

Three studies have assessed probiotics versus placebo to induce remission in 54 patients with CD.^178^, ^179^, ^180^ One study demonstrated improvement in Crohn's disease activity index for the probiotic *Saccharomyces boulardii* (*n* = 10)^178^ compared to placebo (*n* = 7) and two showed no difference between probiotics *Lactobacillus rhamnosus* GG and *E. coli* Nissle 1917 (6/19 [32%]) compared to placebo (6/18 [33%]).^179^, ^180^

## REMISSION MAINTENANCE

### Whole food or elimination diets

**Practice Statement**

There is a lack of evidence to recommend any specific elimination diet for maintaining remission in patients with inflammatory bowel disease. Agreement 85.2%.

There is much interest in aspects of the modern Western lifestyle that may contribute to intestinal inflammation in IBD. From a dietary perspective, processed foods and the use of preservatives and emulsifiers may be important and studies are underway to evaluate these dietary components.

In a retrospective case–control study, a diet that excluded immunoreactive foods was compared to a control diet to maintain Crohn's disease remission for 3 months following EEN to induce disease remission.^181^ Disease relapse was similar between groups (*p* = 0.337) with relapse in four of 32 in the exclusion diet group and eight of 32 in the control diet group after 3 months.

In a prospective pilot RCT, CDED with partial enteral nutrition was compared to CDED without partial enteral nutrition.^112^ The CDED is described as a progressive high protein, low animal fat, low haem, low gluten, and low additive diet with exposure to fibre. Remission maintenance was compared at 24 weeks in the intention to treat group (*n* = 40). In the CDED with partial enteral nutrition group, from week 13, patients did not have to include any enteral nutrition and were only allowed to eat foods from CDED, which is the same intervention as CDED without partial enteral nutrition. At 6 weeks, 25 patients were in remission and, at 24 weeks, 20 remained in remission, 12 of 19 patients from the CDED with partial enteral nutrition group and eight of 21 patients from the CDED without partial enteral nutrition group (*p* = 0.113).^112^

Further trials are awaited; however, no recommendations can be made on the use of such diets at this time.^114^, ^125^, ^182^

#### Anti‐inflammatory diet

**Practice Statement**

There is insufficient evidence to recommend the anti‐inflammatory diet to maintain inflammatory bowel disease remission. Agreement 94.1%.

An anti‐inflammatory diet comprising foods rich in dietary fibre, prebiotics (fructans and galacto‐oligosaccharides), antioxidants (fruits and vegetables), probiotics (fermented foods) and omega‐3 polyunsaturated fatty acids (oily fish), as well as low in red meat, sugar and alcohol, was assessed in an RCT of 28 UC patients in remission with a high risk of bias.^183^ It showed similar relapse rates and faecal calprotectin at 6 months for the anti‐inflammatory diet (5/14) compared to healthy eating advice based on Canada's Food Guide (4/14). The group following Canada's Food Guide had a significant increase in faecal calprotectin over 6 months but the group on the anti‐inflammatory diet did not. There is a high chance of a type 2 error in this study and large multi‐centre RCTs demonstrating a positive effect on relapse rates and mechanistic understanding are needed before this diet can be recommended.

### Enteral nutrition

**Statement**

Partial enteral nutrition alongside routine medication may support Crohn's disease remission maintenance. (GRADE very low quality) agreement 88.0%.

Three RCTs addressed the efficacy of partial enteral nutrition (oral nutritional supplementation) (420–1200 kcal day^–1^) for maintaining remission in CD.^184^, ^185^, ^186^ One had low risk of bias,^185^ one had unclear risk of bias^184^ and one had high risk of bias.^186^ Combining the data from these three studies was possible for the outcome of remission maintenance. More patients who received partial enteral nutrition 56 of 94 (60%) maintained remission compared to patients who did not (34/88 [39%]) (OR = 2.34, 95% CI = 1.29–4.24).

Low quality observation studies have investigated the use of partial enteral nutrition (300–900 kcal day^–1^) to prevent loss of response to biologic anti‐tumour necrosis factor α therapy.^187^, ^188^, ^189^, ^190^, ^191^, ^192^, ^193^ However, it is unclear whether there is a beneficial effect compared with no enteral nutrition.

There is no evidence to recommend partial or exclusive enteral nutrition for maintaining remission in patients with UC.

### Complementary and alternative medicine

**Practice Statement**

There is insufficient evidence to recommend the use of complementary or alternative medicinal products to maintain remission in inflammatory bowel disease. Agreement 84.6%.

There is a lack of evidence to recommend complementary and alternative medicines to maintain disease remission in IBD.^142^, ^152^ One RCT investigated 2 g day^–1^ curcumin compared to placebo for 6 months in patients with quiescent UC and reported significantly fewer patients relapsed following supplementation with curcumin (2/43 [5%]) compared to placebo (8/39 [21%]) (*p* = 0.04). Curcumin was given in addition to standard medication (sulfasalazine or mesalamine).^194^ In another RCT in 62 post‐operative patients with Crohn's disease receiving azathioprine, curcumin was no more effective than placebo in preventing endoscopic or clinical recurrence in Crohn's disease at 6 months.^195^

### Fibre

**Practice Statement**

There is insufficient evidence for the use of a high fibre diet or fibre supplementation for maintaining remission in inflammatory bowel disease. Agreement 82.4%.

The recommended intake of fibre is 30 g day^–1^; however, most patients with IBD do not meet this level.^22^, ^72^ Patients perceive that fibre exacerbates symptoms^196^ and therefore often avoid it during a disease flare. However, in stable disease without strictures, there is no research evidence to support restriction of fibre.^197^

In patients with UC in remission, psyllium appears safe to use and could be used alongside standard medication should patients wish to try it.^198^, ^199^

Two systematic reviews of fibre (supplement or whole diet intervention) as a treatment to maintain remission in IBD were performed^129^, ^130^ and, despite identifying five studies including 232 patients with quiescent UC^198^, ^199^, ^200^, ^201^, ^202^ and four studies including 465 patients with quiescent CD,^203^, ^204^, ^205^, ^206^ there was high heterogeneity among studies with varied criteria for assessing outcome and neither meta‐analysis, nor GRADE summary of the evidence was possible.^198^, ^199^, ^200^, ^201^, ^203^, ^204^, ^205^, ^206^ The largest trial of 105 patients compared remission maintenance over 12 months for 1.5 g day^–1^ mesalamine (24/37 [65%]) with 20 g day^–1^ psyllium fibre (21/35 [60%]) or a combination of both treatments (21/30 [70%]) (*p* = 0.67) and concluded that the treatments were equivalent for remission maintenance.^198^ For the other four studies,^199^, ^200^, ^201^, ^202^ either clinical outcomes were not reported or the results were reported as percentages without the data to ascertain the number of patients in each group; therefore, the outcome of remission maintenance in UC could not be analysed using GRADE. One further RCT conducted subsequent to these systematic reviews compared prebiotics (15 g day^–1^ oligofructose and inulin) with placebo for 6 months and showed no difference in relapse rates for prebiotics (11/35) and control (10/41).^207^

Four studies of CD in remission including 465 patients were identified, and all four studies reported disease outcomes.^129^ However, the interventions were heterogenous and the evidence could not be analysed using GRADE. One study of 20 patients showed that a low fibre exclusion diet led to increased remission maintenance rates compared to a ‘fibre‐rich’ refined carbohydrate diet^205^; however, the other three studies of 445 patients showed no difference between placebo and fibre intervention in clinical outcomes.^203^, ^204^, ^205^, ^206^

One study compared the effect of an anti‐IBD diet (a diet low in animal fat, grains, additives, and high in monounsaturated and ω‐3 fatty acids) with fructooligosaccharides or placebo and reported that relapse occurred more frequently in the fructooligosaccharides group (6/19 [32%]) than the anti‐IBD diet group (0/16 [0%]) (*p* = 0.035), although neither group was significantly different to the placebo group with respect to the rate of relapse (4/19 [21%]).^208^

An RCT in 32 quiescent IBD patients with functional gut symptoms demonstrated that 12 g day^–1^ fructans exacerbated abdominal pain, bloating, flatulence and urgency compared to a placebo (12 g day^–1^ glucose) after 3 days of supplementation.^209^

### Nutrients

#### Vitamin D

**Statement**

There is insufficient evidence to support the use of high dose vitamin D for maintaining remission in Crohn's disease. GRADE very low quality agreement 81.6%.

Two RCTs in 128 patients with quiescent CD and low risk of bias reported 12‐month supplementation with vitamin D.^210^, ^211^ One study reported six of 46 (13%) patients taking 1200 IU day^–1^ vitamin D relapsed compared to 14 of 48 (29%) taking placebo (*p* = 0.056),^210^ whereas the second study reported six of 18 (33%) patients taking 10,000 IU day^–1^ relapsed compared to 11 of 16 (69%) taking 1000 IU day^–1^.^211^ GRADE assessment showed very low quality evidence that vitamin D supplementation had no significant effect on relapse rates at 12 months. As a general health measure vitamin D should be monitored and replaced in deficient individuals.

### Probiotics

**Practice Statement**

There is no evidence for probiotics to provide benefit to maintain disease remission in Crohn's disease. Agreement 90.2%.

Six systematic reviews of probiotics to maintain remission were assessed for RCTs that met the inclusion criteria.^154^, ^155^, ^158^, ^159^, ^161^, ^212^ Three studies have assessed probiotics versus placebo to maintain disease remission for 4 weeks to 12 months in CD.^213^, ^214^, ^215^ None of the studies demonstrated lower relapse rates for probiotic (52/133 [39%]) compared to placebo (50/125 [40%]).

**Practice Statement**

There is insufficient evidence to recommend probiotics to maintain disease remission in ulcerative colitis. Agreement 81.8%.

Four studies have assessed probiotics versus placebo to maintain disease remission in UC.^172^, ^214^, ^216^, ^217^ One study showed that probiotics (3/15 [20%]) had a lower relapse rate compared to placebo (14/15 [93%]) following 8 weeks,^172^ whereas the other studies showed similar rates between probiotics (29/83 [35%]) and placebo (32/61 [52%]) at 4 weeks^214^ and 12 months.^216^, ^217^

## SURGERY

Four systematic reviews have assessed the effect of exclusive enteral nutrition on outcomes in pre‐surgical CD.^218^, ^219^, ^220^, ^221^ Ten retrospective case–control studies met the inclusion criteria, and one of these was a study reported subsequent to the systematic reviews. Two studies had low risk of bias,^222^, ^223^ seven had unclear risk of bias^224^, ^225^, ^226^, ^227^, ^228^, ^229^, ^230^ and one had high risk of bias.^231^

These studies reported on at least one of length of post‐surgical hospital stay, post‐surgical infectious complications or intra‐operative stoma rate in patients with CD undergoing surgery for bowel resection, fistula repair or abscess not suitable for drainage. All of studies compared those patients who received pre‐surgical exclusive enteral nutrition to patients who did not receive any nutrition intervention prior to surgery. Most information was from studies at low or unclear risk of bias using the risk of bias tool specifically for assessing case–control studies.

**Statement**

There is limited evidence that pre‐surgical exclusive enteral nutrition may reduce the length of post‐surgical hospitalisation in patients with Crohn's disease. (GRADE very low quality). Agreement 84.3%.

Three studies reported mean length of post‐surgical hospitalisation was 7.9 days in 288 patients who received pre‐operative exclusive enteral nutrition compared to 9.9 days in 200 patients who received no nutrition intervention. Between 2 and 8 weeks (mean 6 weeks) of pre‐surgical exclusive enteral nutrition was associated with a mean of 2.0 days of reduction in the length of hospital stay.^222^, ^228^, ^232^

One study reported that mean length of post‐surgical hospitalisation for high risk surgical patients (*n* = 35) was 7.5 (2.2) days after 3 weeks of pre‐surgical exclusive enteral nutrition, which is similar to the length of stay (8.3 [6.2] days) (*p* = 0.222) of low‐risk surgical patients who did not receive pre‐surgical nutrition (*n* = 21).^231^

**Statement**

Pre‐surgical exclusive enteral nutrition may lower the risk of post‐surgical infectious complications in patients with Crohn's disease. (GRADE very low quality). Agreement 92.0%.

Nine studies reported the rate of post‐surgical infectious complications in patients receiving pre‐surgical exclusive enteral nutrition for 2–12 weeks (mean 6 weeks) was 93 of 720 (13%) compared to a rate of 158 of 551 (29%) in patients with no nutrition intervention.^222^, ^223^, ^224^, ^225^, ^226^, ^228^, ^230^, ^231^, ^232^ Eight studies reported a significantly lower rate of post‐surgical infectious complication in patients provided with exclusive enteral nutrition.^222^, ^223^, ^224^, ^225^, ^226^, ^228^, ^230^, ^232^ One study did not report a significant difference because it compared high risk patients with exclusive enteral nutrition to low‐risk patients without exclusive enteral nutrition.^231^

Low quality evidence suggests the rate of intra‐operative stoma formation in patients receiving pre‐surgical exclusive enteral nutrition may be lower than those receiving standard care,^224^, ^226^, ^228^, ^231^ although this requires validation in high quality studies.

Similarly, two of the studies reported avoidance of surgery in more patients after starting pre‐surgical exclusive enteral nutrition compared to standard care.^222^, ^228^

## STRICTURES

**Statement**

Exclusive enteral nutrition for 4–12 weeks may induce remission in Crohn's disease patients with inflammatory strictures. (GRADE very low quality). Agreement 84.3%.

Four case series studies^233^, ^234^, ^235^, ^236^ met the inclusion criteria and reported on at least one of remission or bowel wall thickness in patients with inflammatory or fibrous strictures following treatment with EEN. Information was from two studies at unclear risk of bias^233^, ^236^ and two studies at high risk of bias.^234^, ^235^

Combined results from four studies showed that 98 of 114 (85%) patients with stricturing CD achieved remission following exclusive enteral nutrition between 4 and 12 weeks.^233^, ^234^, ^235^, ^236^ Three studies reported validated measures of clinical response (>70 point reduction in CDAI) and remission (CDAI < 150)^233^, ^235^, ^236^ and one study measured remission as a return to pre‐relapse well‐being.^234^ Despite EEN, 30 of 114 (26%) patients with stricturing CD required surgery after 4–12 weeks of EEN. One study had some overlap between patients that entered remission (reporting pre‐relapse level of well‐being) and those that went on to require surgery.^234^

Two case series reported a reduction in bowel wall thickness following EEN in stricturing IBD.^233^, ^236^ Controlled studies are needed to confirm what impact EEN has on bowel wall thickness and the duration needed to achieve clinical remission and mucosal healing.

## POUCHITIS

**Statement**

A probiotic mixture of eight bacterial strains (De Simone Formulation) may maintain remission in chronic relapsing pouchitis. Very limited evidence may support prophylactic use after pouch formation surgery to prevent initial pouchitis onset. There is no evidence to support the use of other probiotics to maintain remission in pouchitis. (GRADE low quality). Agreement 85.4%.

Five RCTs met the inclusion criteria and reported the effect of probiotics on at least one of maintenance of remission in quiescent pouchitis, reduction in pouch disease activity score in active pouchitis or quality of life. All studies compared probiotic treatment to placebo. Information was from three studies at low risk of bias,^173^, ^174^, ^175^ one study at unclear risk of bias^176^ and one study at high risk of bias.^177^

Five RCTs reported rates of remission maintenance in patients with chronic or naïve pouchitis. Four studies used a probiotic mixture of 8 bacterial strains (De Simone Formulation) and showed that 68 of 76 (89%) patients maintained remission compared to 24 of 68 (35%) in the placebo group.^173^, ^174^, ^175^, ^176^ Two of these studies measured IBD quality of life scores and reported an improvement with the probiotic.^174^, ^175^ One further study showed *Bifidobacterium longum* BB‐536 maintained remission in six of seven (86%) patients compared to three of five (60%) patients in the placebo group, although the study was underpowered to detect a difference.^177^

Two other studies showed no benefit of probiotic over placebo in reducing pouch disease activity score, however the sample sizes were relatively small and duration of studies was short.^237^, ^238^

## STOMA

**Practice Statement**

Appetite may be reduced following ileostomy or colostomy formation so encourage small frequent energy dense meals to meet nutritional requirements. Agreement 81.6%.

There are no studies on dietary intake immediately following ileostomy or colostomy formation. The appetite may be reduced, so encourage small frequent energy dense meals to meet nutritional requirements and use oral nutritional supplements where appropriate.

**Practice Statement**

Encourage a wide variety of foods for people with an ileostomy or colostomy to ensure a healthy varied diet. No foods are specifically contraindicated, however some foods have been associated with problems. Agreement 94.0%.

There is limited evidence on the dietary management of an ileostomy or colostomy. A wide variety of foods is encouraged. Various foods have been associated with odour, flatus, increased residue, irritation and increased water content of stoma output (Table 9).^239^, ^240^, ^241^, ^242^, ^243^, ^244^, ^245^, ^246^ Not all of these foods are likely to cause symptoms in isolation and larger portions, or multiple foods from one group within a meal, are more likely to cause symptoms whereas small portions may be well tolerated.

**Table 9:** Foods potentially leading to symptoms associated with stoma

Symptom	Foods
Stool odour	asparagus, beans, broccoli, Brussels sprouts, cabbage, cauliflower, egg, fish, garlic, onions
Increased flatus	beans and pulses, beer and lager, broccoli, Brussels sprouts, cabbage, carbonated drinks, cauliflower, cucumber, turnip
Increased stoma residue	beans and pulses, cabbage (raw only), carrot (raw only), celery, citrus fruit, coconut, dried fruit, fruit and vegetable skins, lettuce, mushrooms, nuts and seeds, pineapple, popcorn, sweetcorn, tomatoes
Irritation	carrot (raw only), chilli, citrus fruit, nuts and seeds
Increased liquid stoma volume	alcohol, strawberries, grapes, peaches, raisins, bananas, prune juice, baked beans, whole wheat cereals, sweetcorn, apples, potatoes, bread, pineapple, pears, rhubarb spicy food, Chinese food, fried food

*Note*: Not all of these foods are likely to cause symptoms in isolation and larger portions, or multiple foods from one group within a meal, are more likely to cause symptoms whereas small portions may be well tolerated.

Many of the foods associated with increased flatus and increased liquid stoma volume are also high in FODMAPs. FODMAPs are short‐chain fermentable carbohydrates that have been associated with increased delivery of endogenous fluid into the gastrointestinal lumen. An RCT of a high versus a low FODMAP diet in 10 patients with an ileostomy and no active disease demonstrated that the low FODMAP diet reduced the wet and dry stoma content; however, the reduction may not be clinically significant.^247^ Whether a low FODMAP diet is beneficial in patients with an ileostomy to maintain hydration status alongside a reduction in stoma volume has not been assessed.

**Practice Statement**

Encourage patients with an ileostomy or colostomy to have a dietary fibre intake in line with population recommendations. Dietary fibre may reduce the volume of stoma output but it depends on the physiological properties of the dietary fibre. Agreement 80.4%.

The evidence does not support recommending a different fibre intake for people with an ileostomy or colostomy compared with the general population. Dietary fibre may reduce the volume of stoma output but it depends on the physiological properties of the dietary fibre. A study in 10 patients (CD, *n* = 5; UC, *n* = 5) with an ileostomy compared a diet high in sucrose and refined cereals with a diet low in sucrose and unrefined cereals. They showed that effluent weight was lower for the low sucrose and unrefined cereal diet compared with the high sucrose and refined cereal diet.^248^

A study in 38 patients with an ileostomy investigated the effect of adding 7 g day^–1^ pysllium husk to a low fibre diet on stoma bag use, as a proxy for stoma output volume.^249^ The addition of pysllium husk resulted in decreased output volume and patients used one ostomy bag less per day than patients who did not use pysllium husk.

## FISTULA

Two uncontrolled case series studies met the inclusion criteria (Table 1) and reported on rates of fistula closure in patients with CD after 12 weeks treatment with EEN. Because the only data available is from uncontrolled studies these were assessed using the QATQS risk of bias tool as recommended in the Cochrane handbook, both studies were considered weak evidence or at high risk of bias.^236^, ^250^

**Statement**

There is very limited evidence that fistulating Crohn's disease may respond to exclusive enteral nutrition. (GRADE very low quality). Agreement 82.0%.

Combined results from two studies, one at unclear risk of bias^236^ and one at high risk of bias,^250^ showed that 57 of 81 (70%) patients that received 12 weeks of EEN had fistula closure by the end of treatment.^236^, ^250^ These findings may be exaggerated because patients unable to tolerate EEN were excluded from analysis; therefore, the data are per protocol only. There is no evidence that EEN is superior to standard care because there was no comparator group in either study.

## SHORT BOWEL SYNDROME

**Practice Statement**

Parenteral nutrition is required where patients cannot meet their nutritional needs via the enteral route. Agreement 91.8%.

Parenteral nutrition is beyond the scope of these guidelines and recommendations are available in the ESPEN 2017 guidelines on chronic intestinal failure in adults.^251^ Use of the enteral nutrition and/or oral diet is to be encouraged where it is not contraindicated to stimulate intestinal adaptation. Avoid long periods of patients being left nil by mouth.

**Practice Statement**

Site and extent of resection and integrity of the remaining bowel are important considerations in the clinical assessment of short bowel syndrome. Agreement 97.9%.

The normal small intestine varies hugely in length, however following resection a residual length of <200 cm is an indication of a short bowel and can lead to nutritional deficiencies if not appropriately managed. For patients without a colon (jejunostomy), the ability to reabsorb fluid and electrolytes is lost. Where the residual jejunal length is <100 cm, parenteral replacement of fluids and electrolytes will be required. Where the jejunal length is <50 cm, long‐term parenteral nutrition will additionally be required.^252^ Understanding the integrity and which parts of the residual ileum and colon are present will determine what absorptive capacity remains; thus, where disease is still present, longer lengths of bowel may lead to short bowel syndrome.

**Practice Statement**

Encourage patients with short bowel syndrome to eat a wide variety of normal foods to compensate for hyperphagia caused by malabsorption. Agreement 84.8%.

Energy and macronutrient absorption are limited in patients with short bowel syndrome. Three studies in patients with ileum <200 cm (12 patients with a jejunostomy and 46 patients with a jejuno‐colic anastomosis) have demonstrated that absorption of energy and macronutrients is approximately two‐thirds of dietary intake indicating a need for hyperphagic diets in short bowel syndrome.^253^, ^254^, ^255^ Energy and protein requirements may be increased from 30 to 60 kcal kg^–1^ day^–1^ and 1.25–1.5 g kg^–1^ day^–1^, respectively.^253^, ^254^, ^255^

**Practice Statement**

The bowel adapts over time to improve nutrient absorptive capacity agreement 91.7%.

Following extensive ileal resection, parenteral nutrition is often needed in the short term. It is important that the enteral route is used where possible because hormonal growth factors, glucagon like growth factor‐2, nutrients, bile and pancreatic secretions will enable morphological and functional adaptation of the gut, particularly distal to the anastomosis.^256^ The ileum dilates and increased villi crypt height and depth improve tolerance of enteral nutrition and oral diet.^256^ The process can take months or even years and the exact mechanisms are not fully understood.

**Practice Statement**

In patients with short bowel syndrome where the colon is preserved (jejuno‐colic anastomosis), a diet high in complex carbohydrates and low in fat is advised. Consider using medium‐chain triglycerides to replace some long‐chain triglycerides and ensure the diet is not deficient in essential fatty acids and fat‐soluble vitamins. Agreement 89.8%.

In patients with a jejuno‐colic anastomosis where small bowel length <200 cm, a diet containing 50%–60% energy from carbohydrate enables salvage of up to 1000 kcal day^–1^ from fermentation of unabsorbed carbohydrates in the colon.^257^, ^258^

A low fat diet (20%–30% energy from fat) can reduce diarrhoea; however, it may be unpalatable. Substituting long‐chain triglycerides with medium‐chain triglycerides increases fat absorption from 23% to 58% and energy absorption from 46% to 58% without increasing stool output.^259^

Ensure the diet contains adequate amounts of essential fatty acids and fat‐soluble vitamins and, if not, use supplements to prevent deficiency.

**Practice Statement**

In patients with short bowel syndrome and a jejunostomy, a diet with normal carbohydrate and fat intakes are advised. Agreement 89.1%

In patients with short bowel syndrome and a jejunostomy, advise a normal amount of energy from carbohydrate (40%–50%) and fat (30%–40%) in the diet. Evidence indicates that patients with a jejunostomy benefit from the concentrated source of fat in the diet rather than a low fat diet.^253^

**Practice Statement**

In patients with short bowel syndrome where the colon is preserved (jejuno‐colic anastomosis), a diet low in oxalates will prevent renal calculi. Agreement 80.4%.

Preferential binding of calcium and magnesium to unabsorbed long‐chain fatty acids in the colon releases oxalate which may be absorbed and predispose patients to developing renal calculi. A diet low in oxalates and moderate in fat is advised (Table 10).^252^

**Practice Statement**

Lactose intolerance in short bowel syndrome should be assessed on a case by case basis. Agreement 88.0%.

**Table 10:** Dietary sources of oxalates that should be avoided in patients with short bowel syndrome and a colon

Spinach Beetroot Rhubarb Chocolate Cocoa and carob Peanuts and almonds Bran flakes Tea (more than 2–3 cups day^–1^) Parsley

*Note*: Oxalate content may vary by country of origin, season and cooking method. Advise avoidance of high oxalate foods alongside an adequate calcium intake (800–1200 mg day^–1^) or calcium supplement if dietary intake is inadequate.^260^

A study in 14 stable patients with short bowel syndrome demonstrated that lactose was well tolerated as a 20‐g load or as part of a normal lactose containing diet; thus, there is no need to routinely restrict lactose.^261^, ^262^

**Practice Statement**

An increase in dietary fibre intake does not improve macronutrient or fluid absorption in short bowel syndrome. Agreement 82.6%.

A study in six patients with a jejuno‐colic anastomosis showed that pectin fibre had little effect on colonic fermentation and starch was more important for macronutrient and fluid absorption.^263^ The bulking effect of dietary fibre is not helpful in short bowel syndrome and dietary fibre can inhibit fat and mineral absorption.^264^ Thus, assess dietary fibre intake and consider a trial of fibre reduction if stool output is high. Anecdotally, some patients with a jejunostomy find taking a fibre supplement improves (thickens) effluent consistency.

**Practice Statement**

In patients with a jejunostomy at risk of dehydration, advise use an oral rehydration solution to optimise sodium and fluid absorption. Agreement 95.7%.

To optimise sodium and electrolyte absorption in jejunostomy patients at risk of dehydration as a result of increased gastrointestinal losses, limit hypotonic and hypertonic oral fluids to 500 ml day^–1^. Give an oral rehydration solution containing 90 mmol L^–1^ sodium (20 g glucose, 3.5 g sodium chloride and 2.5 g sodium bicarbonate dissolved in 1 L of water).^265^, ^266^, ^267^

**Practice Statement**

In all patients with a jejunostomy, encourage sprinkling salt onto food. Add 0.5–1 g of salt per day. Agreement 87.5%.

Adding salt to food (0.5–1 g day^–1^) is useful for maintaining good hydration status in patients with a jejunostomy.

## FUNCTIONAL BOWEL SYMPTOMS

In line with clinical guidelines, patients with IBD and functional gut symptoms should be provided with first line dietary and lifestyle advice before implementing any dietary restrictions.^268^, ^269^, ^270^, ^271^ Three RCTs met the inclusion criteria and reported on at least one of global symptom improvement of functional symptoms or improvement in composite functional symptom score in patients with IBD following a low FODMAP diet compared to either a placebo diet or habitual diet. Information was from two studies at unclear risk of bias ^121^, ^272^ and one study at high risk of bias.^120^

**Statement**

A low FODMAP diet may improve global functional bowel symptoms in quiescent or mildly active inflammatory bowel disease. (GRADE very low quality). Agreement 90.2%.

Two studies reported a combined global symptom response to the low FODMAP diet of 44 of 71 (62%) compared to 23 of 70 (33%) following a control diet (OR = 3.3, 95% CI = 1.7–6.7) (*p* = 0.0007).^120^, ^121^ One study was an RCT that compared the low FODMAP diet with a placebo sham diet ^121^ and the other study was a non‐blinded study comparing the low FODMAP diet with habitual diet^120^; therefore, the evidence was downgraded.

**Practice Statement**

Dietitian‐led low FODMAP education in inflammatory bowel disease may be used for persistent bowel symptoms in the absence of inflammation. This involves short‐term FODMAP restriction, FODMAP reintroduction to tolerance and personalisation for long‐term management. Agreement 93.9%.

Two studies reported symptom scores following the low FODMAP diet compared a control diet.^120^, ^272^ One study was a small cross‐over study in patients with quiescent CD (*n* = 9) and reported that visual analogue scale scores were lower in the last 2 weeks following the low FODMAP diet (13.5 mm, 95% CI = 5.9–21.1) compared to the typical Australian diet (24.8 mm, 95% CI = 12.6–37.0) (*p* < 0.001).^272^ The second study was an open label study in both active and inactive IBD and reported that median (IQR) irritable bowel syndrome severity scoring system (IBS‐SSS) scores at week 6 were lower (115 [33–169]) after the low FODMAP diet compared to the habitual diet (170 [91–288]) (*p* = 0.02).^120^

Two studies have assessed provocation with FODMAPs in IBD patients with functional symptoms who had first been instructed to follow a low FODMAP diet.^209^, ^273^ One RCT in 32 patients demonstrated that 12 g day^–1^ fructans exacerbated abdominal pain, bloating, flatulence and urgency compared to a placebo (12 g day^–1^ glucose) after 3 days of supplementation,^209^ whereas the other in 19 patients showed that provocation with FODMAPs and placebo led to similar pain and bloating levels as at baseline.^273^

## SPECIAL SITUATIONS

### Extra‐intestinal manifestations

**Practice Statement**

There is no evidence for providing dietary advice to manage extraintestinal manifestations of inflammatory bowel disease, however optimisation of nutritional status and induction of disease remission are important considerations. Agreement 91.8%.

Refer to BSG guidelines and ECCO guidelines on how to manage extra‐intestinal manifestations of IBD.^47^, ^274^, ^275^

### OFG

One case series met the inclusion criteria and reported on either clinical response in patients with OFG following a nutrition intervention.

**Practice Statement**

A cinnamon and benzoate free diet may be effective in reducing oral disease activity score in patients with orofacial granulomatosis. Agreement 93.6%.

Patients with OFG (*n* = 32) received dietary advice on a cinnamon and benzoate free diet for 8 weeks. Only 25 patients completed the study and nine patients had gut involvement. A clinical response was reported in 18 of 25 (72%) patients.^276^ A cinnamon and benzoate free diet is a first‐line treatment option for patients with OFG; however, the mechanisms are not understood. Dietary resources on a cinnamon and benzoate free diet are available online (www.kcl.ac.uk/ofg) for healthcare professionals working with patients with OFG.

### Upper gastrointestinal Crohn's disease

**Practice Statement**

There is no evidence for providing dietary advice to manage upper gastrointestinal Crohn's disease; however, optimisation of nutritional status and induction of disease remission are important considerations. Agreement 83.3%.

Proton pump inhibitors are often used to treat upper gastrointestinal Crohn's disease and may affect iron, vitamin B_12_, vitamin C and vitamin D, and possibly magnesium status, as well as bone mineral density. Refer to BSG guidelines and ECCO guidelines on how to manage upper gastrointestinal Crohn's disease.^47^, ^101^, ^274^

### Perianal IBD

**Practice Statement**

There is no evidence for providing dietary advice to manage perianal inflammatory bowel disease; however, optimisation of nutritional status and induction of disease remission are important considerations. Agreement 96.0%.

Refer to BSG guidelines and ECCO guidelines on how to manage perianal IBD.^47^, ^274^, ^277^

## CONCLUSIONS

These British Dietetic Association consensus guidelines on the nutritional assessment and dietary management of IBD have been developed with input from all the major UK stakeholders involved in the management of IBD including patient representation through collaboration with Crohn's and Colitis UK. Involving the IBD multi‐disciplinary team has enabled these guidelines to consider that patient choice is central to the development of treatment plans.

The guidelines have been developed to improve access to the current research evidence and ultimately aim to increase equality of care in nutritional assessment and dietary management of IBD. They comprise the most up‐to‐date recommendations with statements and practice statements agreed by consensus on nutritional assessment and nutritional screening tools, as well as dietary management strategies, for treatment of malnutrition; induction and maintenance of disease remission; surgery; stricturing disease; pouchitis; stoma; fistula; short bowel syndrome; functional bowel symptoms; and special situations including OFG.

## CONFLICT OF INTERESTS

The authors have no conflicts of interest. All members of the eDelphi Consensus Group signed conflicts of interest forms during the development of these guidelines.

## AUTHOR CONTRIBUTIONS

Miranda C. E. Lomer was the grant holder. Miranda C. E. Lomer is the IBD lead of the Gastroenterology Specialist Group of the BDA. Miranda C. E. Lomer, Bridgette Wilson and Catherine L. Wall were involved in the conception, development and analysis of the guidelines. Miranda C. E. Lomer and Catherine L. Wall recruited the expert IBD panel. Miranda C. E. Lomer recruited members of the IBD community to take part in the consensus voting. Miranda C. E. Lomer, Bridgette Wilson and Catherine L. Wall wrote, edited and approved the final version of the manuscript submitted for publication.

## Supporting information

Supporting information.

## References

[1] Sigall‐Boneh R , Levine A , Lomer M , Wierdsma N , Allan P , Fiorino G , et al. Research gaps in diet and nutrition in inflammatory bowel disease. A topical review by D‐ECCO Working Group [Dietitians of ECCO]. J Crohns Colitis. 2017;11(12):1407–19. 28961811 10.1093/ecco-jcc/jjx109

[2] Tinsley A , Ehrlich OG , Hwang C , Issokson K , Zapala S , Weaver A , et al. Knowledge, attitudes, and beliefs regarding the role of nutrition in IBD among patients and providers. Inflamm Bowel Dis. 2016;22(10):2474–81. 27598738 10.1097/MIB.0000000000000901

[3] Higgins J , Thomas J . New Cochrane handbook for systematic reviews of interventions. Wiley‐Blackwell; 2019.

[4] Liberati A , Altman DG , Tetzlaff J , Mulrow C , Gøtzsche PC , Ioannidis JP , et al. The PRISMA statement for reporting systematic reviews and meta‐analyses of studies that evaluate health care interventions: explanation and elaboration. Ann Intern Med. 2009;151(4):W65–94. 19622512 10.7326/0003-4819-151-4-200908180-00136

[5] GRADE Working Group . *GRADE handbook for grading quality of evidence and strength of recommendations*; 2013. Available from: https://www.gradeworkinggroup.org/

[6] Adams DW , Gurwara S , Silver HJ , Horst SN , Beaulieu DB , Schwartz DA , et al. Sarcopenia is common in overweight patients with inflammatory bowel disease and may predict need for surgery. Inflamm Bowel Dis. 2017;23(7):1182–6. 28410342 10.1097/MIB.0000000000001128

[7] Büning C , Von Kraft C , Hermsdorf M , Gentz E , Wirth EK , Valentini L , et al. Visceral adipose tissue in patients with Crohn's disease correlates with disease activity, inflammatory markers, and outcome. Inflamm Bowel Dis. 2015;21(11):2590–7. 26222339 10.1097/MIB.0000000000000527

[8] Cuoco L , Vescovo G , Castaman R , Ravara B , Cammarota G , Angelini A , et al. Skeletal muscle wastage in Crohn's disease: a pathway shared with heart failure? Int J Cardiol. 2008;127(2):219–27. 17692969 10.1016/j.ijcard.2007.06.006

[9] Emerenziani S , Biancone L , Guarino MPL , Balestrieri P , Stasi E , Ribolsi M , et al. Nutritional status and bioelectrical phase angle assessment in adult Crohn disease patients receiving anti‐TNFalpha therapy. Dig Liver Dis. 2017;49(5):495–9. 28096060 10.1016/j.dld.2016.12.026

[10] Gilman J , Shanahan F , Cashman KD . Altered levels of biochemical indices of bone turnover and bone‐related vitamins in patients with Crohn's disease and ulcerative colitis. Aliment Pharmacol Ther. 2006;23(7):1007–16. 16573803 10.1111/j.1365-2036.2006.02835.x

[11] Hengstermann S , Valentini L , Schaper L , Buning C , Koernicke T , Maritschnegg M , et al. Altered status of antioxidant vitamins and fatty acids in patients with inactive inflammatory bowel disease. Clin Nutr. 2008;27(4):571–8. 18316141 10.1016/j.clnu.2008.01.007

[12] Katznelson L , Fairfield WP , Zeizafoun N , Sands BE , Peppercorn MA , Rosenthal DI , et al. Effects of growth hormone secretion on body composition in patients with Crohn's disease. J Clin Endocrinol Metab. 2003;88(11):5468–72. 14602791 10.1210/jc.2003-030608

[13] Magro DO , Barreto MRL , Cazzo E , Camargo MG , Kotze PG , Coy CSR . Visceral fat is increased in individuals with Crohn's disease: a comparative analysis with healthy controls. Arq Gastroenterol. 2018;55(2):142–7. 30043863 10.1590/S0004-2803.201800000-25

[14] Mingrone G , Capristo E , Greco AV , Benedetti G , De Gaetano A , Tataranni PA , et al. Elevated diet‐induced thermogenesis and lipid oxidation rate in Crohn disease. Am J Clin Nutr. 1999;69(2):325–30. 9989699 10.1093/ajcn/69.2.325

[15] Rizzi M , Mazzulo S , Fregnan S , Leogrande G , Addante I , Regano N , et al. Energy balance and muscle function in patients with Crohn's disease: relationship with nutritional state and disease activity. Nutr Ther Metab. 2012;30(4):197–207.

[16] Valentini L , Schaper L , Buning C , Hengstermann S , Koernicke T , Tillinger W , et al. Malnutrition and impaired muscle strength in patients with Crohn's disease and ulcerative colitis in remission. Nutrition. 2008;24(7–8):694–702. 18499398 10.1016/j.nut.2008.03.018

[17] Wiroth JB , Filippi J , Schneider SM , Al‐Jaouni R , Horvais N , Gavarry O , et al. Muscle performance in patients with Crohn's disease in clinical remission. Inflamm Bowel Dis. 2005;11(3):296–303. 15735436 10.1097/01.mib.0000160810.76729.9c

[18] Benjamin J , Makharia G , Ahuja V , Joshi YK . Body composition in Indian patients with Crohn's disease during active and remission phase. Trop Gastroenterol. 2011;32(4):285–91. 22696909

[19] Capristo E , Addolorato G , Mingrone G , Greco AV , Gasbarrini G . Effect of disease localization on the anthropometric and metabolic features of Crohn's disease. Am J Gastroenterol. 1998;93(12):2411–9. 9860401 10.1111/j.1572-0241.1998.00696.x

[20] Capristo E , Mingrone G , Addolorato G , Greco AV , Gasbarrini G . Metabolic features of inflammatory bowel disease in a remission phase of the disease activity. J Intern Med. 1998;243(5):339–47. 9651555 10.1046/j.1365-2796.1998.00254.x

[21] Filippi J , Al‐Jaouni R , Wiroth JB , Hebuterne X , Schneider SM . Nutritional deficiencies in patients with Crohn's disease in remission. Inflamm Bowel Dis. 2006;12(3):185–91. 16534419 10.1097/01.MIB.0000206541.15963.c3

[22] Geerling BJ , Badart‐Smook A , Stockbrugger RW , Brummer RJ . Comprehensive nutritional status in patients with long‐standing Crohn disease currently in remission. Am J Clin Nutr. 1998;67(5):919–26. 9583850 10.1093/ajcn/67.5.919

[23] Geerling BJ , v Houwelingen AC , Badart‐Smook A , Stockbrugger RW , Brummer RJ . Fat intake and fatty acid profile in plasma phospholipids and adipose tissue in patients with Crohn's disease, compared with controls. Am J Gastroenterol. 1999;94(2):410–7. 10022638 10.1111/j.1572-0241.1999.869_a.x

[24] Geerling BJ , Badart‐Smook A , Stockbrügger RW , Brummer RJM . Comprehensive nutritional status in recently diagnosed patients with inflammatory bowel disease compared with population controls. Eur J Clin Nutr. 2000;54(6):514–21. 10878655 10.1038/sj.ejcn.1601049

[25] Ghoshal UC , Shukla A . Malnutrition in inflammatory bowel disease patients in northern India: frequency and factors influencing its development. Trop Gastroenterol. 2008;29(2):95–7. 18972769

[26] Grunbaum A , Holcroft C , Heilpern D , Gladman S , Burstein B , Menard M , et al. Dynamics of vitamin D in patients with mild or inactive inflammatory bowel disease and their families. Nutr J. 2013;12(1):145. 24206944 10.1186/1475-2891-12-145 PMC3828424

[27] Jahnsen J , Falch JA , Mowinckel P , Aadland E . Body composition in patients with inflammatory bowel disease: A population‐based study. Am J Gastroenterol. 2003;98(7):1556–62. 12873577 10.1111/j.1572-0241.2003.07520.x

[28] Kawakami Y , Murakami Y , Kawakami T , Muroyama N , Takiue T , Moritani Y , et al. Abnormal fatty acid profile of blood cell phospholipids and dietary fatty acid intake in patients with ulcerative colitis. J Clin Biochem Nutr. 2005;37(3):95–102.

[29] Mijač DD , Janković GLJ , Jorga J , Krstić MN . Nutritional status in patients with active inflammatory bowel disease: prevalence of malnutrition and methods for routine nutritional assessment. Eur J Intern Med. 2010;21(4):315–9. 20603043 10.1016/j.ejim.2010.04.012

[30] Molnar A , Csontos AA , Kovacs I , Anton AD , Palfi E , Miheller P . Body composition assessment of Crohn's outpatients and comparison with gender‐ and age‐specific multiple matched control pairs. Eur J Clin Nutr. 2017;71(10):1246–50. 28656973 10.1038/ejcn.2017.99

[31] Opstelten JL , de Vries JHM , Wools A , Siersema PD , Oldenburg B , Witteman BJM . Dietary intake of patients with inflammatory bowel disease: a comparison with individuals from a general population and associations with relapse. Clin Nutr. 2019;38(4):1892–8. 30049516 10.1016/j.clnu.2018.06.983

[32] Principi M , Losurdo G , Iannone A , Contaldo A , Deflorio V , Ranaldo N , et al. Differences in dietary habits between patients with inflammatory bowel disease in clinical remission and a healthy population. Ann Gastroenterol. 2018;31(4):469–73. 29991892 10.20524/aog.2018.0273 PMC6033751

[33] Schneider SM , Al‐Jaouni R , Filippi J , Wiroth JB , Zeanandin G , Arab K , et al. Sarcopenia is prevalent in patients with Crohn's disease in clinical remission. Inflamm Bowel Dis. 2008;14(11):1562–8. 18478564 10.1002/ibd.20504

[34] Ueda Y , Kawakami Y , Kunii D , Okada H , Azuma M , Le DS , et al. Elevated concentrations of linoleic acid in erythrocyte membrane phospholipids in patients with inflammatory bowel disease. Nutr Res. 2008;28(4):239–44. 19083414 10.1016/j.nutres.2008.02.005

[35] Tjellesen L , Nielsen PK , Staun M . Body composition by dual‐energy X‐ray absorptiometry in patients with Crohn's disease. Scand J Gastroenterol. 1998;33(9):956–60. 9759952 10.1080/003655298750026985

[36] Lu ZL , Wang TR , Qiao YQ , Zheng Q , Sun Y , Lu JT , et al. Handgrip strength index predicts nutritional status as a complement to body mass index in Crohn's disease. J Crohns Colitis. 2016;10(12):1395–400. 27402912 10.1093/ecco-jcc/jjw121

[37] Zaltman C , Braulio VB , Outeiral R , Nunes T , Natividade de Castro CL . Lower extremity mobility limitation and impaired muscle function in women with ulcerative colitis. J Crohns Colitis. 2014;8(6):529–35. 24315794 10.1016/j.crohns.2013.11.006

[38] Bastos CM , Araújo IM , Nogueira‐Barbosa MH , Salmon CEG , de Paula FJA , Troncon LEA . Reduced bone mass and preserved marrow adipose tissue in patients with inflammatory bowel diseases in long‐term remission. Osteoporos Int. 2017;28(7):2167–76. 28405731 10.1007/s00198-017-4014-3

[39] Salacinski AJ , Regueiro MD , Broeder CE , McCrory JL . Decreased neuromuscular function in Crohn's disease patients is not associated with low serum vitamin D levels. Dig Dis Sci. 2013;58(2):526–33. 22949179 10.1007/s10620-012-2372-4

[40] Lopes M , Lyra A , Rocha R , Factum C , Sales L , Mello L , et al. Are ulcerative colitis outpatients at nutritional risk? J Crohns Colitis. 2016;10(Suppl 1):S227.

[41] Sandall AM , Wall CL , Lomer MCE . Nutrition assessment in Crohn's disease using anthropometric, biochemical, and dietary indexes: a narrative review. J Acad Nutr Diet. 2020;120:624–640. 31248791 10.1016/j.jand.2019.04.013

[42] Edwards K , Yearsley K . Unusual presentation of Crohn's disease. BMJ Case Rep. 2021;14(6):64–71. 10.1136/bcr-2021-242703 PMC8191605 34108157

[43] Dodds RM , Syddall HE , Cooper R , Benzeval M , Deary IJ , Dennison EM , et al. Grip strength across the life course: normative data from twelve British studies. PLoS One. 2014;9(12):e113637. 25474696 10.1371/journal.pone.0113637 PMC4256164

[44] Klidjian AM , Foster KJ , Kammerling RM , Cooper A , Karran SJ . Relation of anthropometric and dynamometric variables to serious postoperative complications. Br Med J. 1980;281(6245):899–901. 7427501 10.1136/bmj.281.6245.899 PMC1714169

[45] Dignass AU , Gasche C , Bettenworth D , Birgegård G , Danese S , Gisbert JP , et al. European consensus on the diagnosis and management of iron deficiency and anaemia in inflammatory bowel diseases. J Crohns Colitis. 2015;9(3):211–22. 25518052 10.1093/ecco-jcc/jju009

[46] Maaser C , Sturm A , Vavricka SR , Kucharzik T , Fiorino G , Annese V , et al. ECCO‐ESGAR Guideline for Diagnostic Assessment in IBD Part 1: Initial diagnosis, monitoring of known IBD, detection of complications. J Crohns Colitis. 2019;13(2):144–64. 30137275 10.1093/ecco-jcc/jjy113

[47] Lamb CA , Kennedy NA , Raine T , Hendy PA , Smith PJ , Limdi JK , et al. British Society of Gastroenterology consensus guidelines on the management of inflammatory bowel disease in adults. Gut. 2019;68:1. 10.1136/gutjnl-2019-318484 PMC6872448 31562236

[48] Duncan A , Talwar D , McMillan DC , Stefanowicz F , O'Reilly DS . Quantitative data on the magnitude of the systemic inflammatory response and its effect on micronutrient status based on plasma measurements. Am J Clin Nutr. 2012;95(1):64–71. 22158726 10.3945/ajcn.111.023812

[49] Gerasimidis K , Bronsky J , Catchpole A , Embleton N , Fewtrell M , Hojsak I , et al. Assessment and interpretation of vitamin and trace element status in sick children: a position paper from the European Society for Paediatric Gastroenterology Hepatology, and Nutrition Committee on Nutrition. J Pediatr Gastroenterol Nutr. 2020;70(6):873–81. 32443051 10.1097/MPG.0000000000002688

[50] McMillan DC , Maguire D , Talwar D . Relationship between nutritional status and the systemic inflammatory response: micronutrients. Proc Nutr Soc. 2019;78(1):56–67. 30220267 10.1017/S0029665118002501

[51] Yakut M , Ustun Y , Kabacam G , Soykan I . Serum vitamin B12 and folate status in patients with inflammatory bowel diseases. Eur J Intern Med. 2010;21(4):320–3. 20603044 10.1016/j.ejim.2010.05.007

[52] Kallel L , Feki M , Sekri W , Segheir L , Fekih M , Boubaker J , et al. Prevalence and risk factors of hyperhomocysteinemia in Tunisian patients with Crohn's disease. J Crohns Colitis. 2011;5(2):110–4. 21453879 10.1016/j.crohns.2010.10.010

[53] Duggan P , O'Brien M , Kiely M , McCarthy J , Shanahan F , Cashman KD . Vitamin K status in patients with Crohn's disease and relationship to bone turnover. Am J Gastroenterol. 2004;99(11):2178–85. 15555000 10.1111/j.1572-0241.2004.40071.x

[54] Ardizzone S , Bollani S , Bettica P , Bevilacqua M , Molteni P , Bianchi Porro G . Altered bone metabolism in inflammatory bowel disease: there is a difference between Crohn's disease and ulcerative colitis. J Intern Med. 2000;247(1):63–70. 10672132 10.1046/j.1365-2796.2000.00582.x

[55] Dumitrescu G , Mihai C , Dranga M , Prelipcean CC . Serum 25‐hydroxyvitamin D concentration and inflammatory bowel disease characteristics in Romania. World J Gastroenterol. 2014;20(9):2392–6. 24605037 10.3748/wjg.v20.i9.2392 PMC3942843

[56] Joseph AJ , George B , Pulimood AB , Seshadri MS , Chacko A . 25 (OH) vitamin D level in Crohn's disease: association with sun exposure & disease activity. Indian J Med Res. 2009;130(2):133–7. 19797809

[57] Suibhne TN , Cox G , Healy M , O'Morain C , O'Sullivan M . Vitamin D deficiency in Crohn's disease: prevalence, risk factors and supplement use in an outpatient setting. J Crohns Colitis. 2012;6(2):182–8. 22325172 10.1016/j.crohns.2011.08.002

[58] Tajika M , Matsuura A , Nakamura T , Suzuki T , Sawaki A , Kato T , et al. Risk factors for vitamin D deficiency in patients with Crohn's disease. J Gastroenterol. 2004;39(6):527–33. 15235869 10.1007/s00535-003-1338-x

[59] Tan B , Li P , Lv H , Li Y , Wang O , Xing XP , et al. Vitamin D levels and bone metabolism in Chinese adult patients with inflammatory bowel disease. J Dig Dis. 2014;15(3):116–23. 24354597 10.1111/1751-2980.12118

[60] Mohammadi E , Qujeq D , Taheri H , Hajian‐Tilaki K . Evaluation of serum trace element levels and superoxide dismutase activity in patients with inflammatory bowel disease: translating basic research into clinical application. Biol Trace Elem Res. 2017;177(2):235–40. 27864666 10.1007/s12011-016-0891-0

[61] Ringstad J , Kildebo S , Thomassen Y . Serum selenium, copper, and zinc concentrations in Crohn's disease and ulcerative colitis. Scand J Gastroenterol. 1993;28(7):605–8. 8362213 10.3109/00365529309096096

[62] Dalekos GN , Ringstad J , Savaidis I , Seferiadis KI , Tsianos EV . Zinc, copper and immunological markers in the circulation of well nourished patients with ulcerative colitis. Eur J Gastroenterol Hepatol. 1998;10(4):331–7. 9855050 10.1097/00042737-199804000-00010

[63] Hinks LJ , Inwards KD , Lloyd B , Clayton B . Reduced concentrations of selenium in mild Crohn's disease. J Clin Pathol. 1988;41(2):198–201. 3350980 10.1136/jcp.41.2.198 PMC1141378

[64] Sturniolo GC , Mestriner C , Lecis PE , D'Odorico A , Venturi C , Irato P , et al. Altered plasma and mucosal concentrations of trace elements and antioxidants in active ulcerative colitis. Scand J Gastroenterol. 1998;33(6):644–9. 9669638 10.1080/00365529850171936

[65] Wendland BE , Aghdassi E , Tam C , Carrrier J , Steinhart AH , Wolman SL , et al. Lipid peroxidation and plasma antioxidant micronutrients in Crohn disease. Am J Clin Nutr. 2001;74(2):259–64. 11470730 10.1093/ajcn/74.2.259

[66] Gentschew L , Bishop KS , Han DY , Morgan AR , Fraser AG , Lam WJ , et al. Selenium, selenoprotein genes and Crohn's disease in a case‐control population from Auckland, New Zealand. Nutrients. 2012;4(9):1247–59. 23112913 10.3390/nu4091247 PMC3475235

[67] Krzystek‐Korpacka M , Neubauer K , Berdowska I , Zielinski B , Paradowski L , Gamian A . Impaired erythrocyte antioxidant defense in active inflammatory bowel disease: impact of anemia and treatment. Inflamm Bowel Dis. 2010;16(9):1467–75. 20186929 10.1002/ibd.21234

[68] Day AS , Yao CK , Costello SP , Andrews JM , Bryant RV . Food avoidance, restrictive eating behaviour and association with quality of life in adults with inflammatory bowel disease: a systematic scoping review. Appetite. 2021;167:167. 10.1016/j.appet.2021.105650 34391842

[69] Lomer MCE , Kodjabashia K , Hutchinson C , Greenfield SM , Thompson RPH , Powell JJ . Intake of dietary iron is low in patients with Crohn's disease: a case‐control study. Br J Nutr. 2004;91(1):141–8. 14748947 10.1079/bjn20041022

[70] D'Odorico A , Bortolan S , Cardin R , D'inca' R , Martines D , Ferronato A , et al. Reduced plasma antioxidant concentrations and increased oxidative DNA damage in inflammatory bowel disease. Scand J Gastroenterol. 2001;36(12):1289–94. 11761019 10.1080/003655201317097146

[71] Sousa Guerreiro C , Cravo M , Costa AR , Miranda A , Tavares L , Moura‐Santos P , et al. A comprehensive approach to evaluate nutritional status in Crohn's patients in the era of biologic therapy: a case‐control study. Am J Gastroenterol. 2007;102(11):2551–6. 17680845 10.1111/j.1572-0241.2007.01439.x

[72] Cox SR , Clarke H , O'Keeffe M , Dubois P , Irving PM , Lindsay JO , et al. Nutrient, fibre and FODMAP intakes and food‐related quality of life in patients with inflammatory bowel disease and their relationship with gastrointestinal symptoms of differing aetiologies. J Crohns Colitis. 2021;15:2041–53. 34216206 10.1093/ecco-jcc/jjab116 PMC8684455

[73] Powell JJ , Cook WB , Chatfield M , Hutchinson C , Pereira DIA , Lomer MCE . Iron status is inversely associated with dietary iron intakes in patients with inactive or mildly active inflammatory bowel disease. Nutr Metab. 2013;10(1):18. 10.1186/1743-7075-10-18 PMC3566950 23374396

[74] Vernia P , Loizos P , Di Giuseppantonio I , Amore B , Chiappini A , Cannizzaro S . Dietary calcium intake in patients with inflammatory bowel disease. J Crohns Colitis. 2014;8(4):312–7. 24090907 10.1016/j.crohns.2013.09.008

[75] Silvennoinen J , Lamberg‐Allardt C , Kärkkäinen M , Niemelä S , Lehtola J . Dietary calcium intake and its relation to bone mineral density in patients with inflammatory bowel disease. J Intern Med. 1996;240(5):285–92. 8946811 10.1046/j.1365-2796.1996.25862000.x

[76] Dell'Era A , De Almeida V , Cioni L , Massari A , Muzio F . Dietetic intervention and nutritional status in adult patients with Crohn's disease: preliminary results. J Crohns Colitis. 2016;10(Supp 1):403–4.

[77] Keetarut K , Zacharopoulou‐Otapasidou S , Bloom S , Majumdar A , Patel PS . An evaluation of the feasibility and validity of a patient‐administered malnutrition universal screening tool ('MUST') compared to healthcare professional screening in an inflammatory bowel disease (IBD) outpatient clinic. J Hum Nutr Diet. 2017;30(6):737–45. 28585266 10.1111/jhn.12481

[78] Wall C , Wilson B , Sanderson J , Lomer M . Development of an inflammatory bowel disease‐specific nutrition screening tool (IBD‐NST). J Crohns Colitis. 2019;13:S415–S.

[79] Haskey N , Pena‐Sanchez JN , Jones JL , Fowler SA . Development of a screening tool to detect nutrition risk in patients with inflammatory bowel disease. Asia Pac J Clin Nutr. 2018;27(4):756–62. 30045418 10.6133/apjcn.112017.01

[80] Csontos AA , Molnar A , Piri Z , Palfi E , Miheller P . Malnutrition risk questionnaire combined with body composition measurement in malnutrition screening in inflammatory bowel disease. Rev Esp Enferm Dig. 2017;109(1):26–32. 27931104 10.17235/reed.2016.4557/2016

[81] Sandhu A , Mosli M , Yan B , Wu T , Gregor J , Chande N , et al. Self‐screening for malnutrition risk in outpatient inflammatory bowel disease patients using the Malnutrition Universal Screening Tool (MUST). JPEN J Parenter Enteral Nutr. 2016;40(4):507–10. 25632031 10.1177/0148607114566656

[82] Takaoka A , Sasaki M , Kurihara M , Iwakawa H , Inoue M , Bamba S , et al. Comparison of energy metabolism and nutritional status of hospitalized patients with Crohn's disease and those with ulcerative colitis. J Clin Biochem Nutr. 2015;56(3):208–14. 26060351 10.3164/jcbn.14-95 PMC4454083

[83] Takaoka A , Sasaki M , Nakanishi N , Kurihara M , Ohi A , Bamba S , et al. Nutritional screening and clinical outcome in hospitalized patients with Crohn's disease. Ann Nutr Metab. 2017;71(3–4):266–72. 29241167 10.1159/000485637

[84] Vigano C , Losco A , Bergna I , Meucci G , Amato A . Risk of malnutrition in patients with inflammatory bowel diseases: results from an Italian multi‐centre observational cross‐sectional study. J Crohns Colitis. 2018;12:S454–S.

[85] Fiorindi C , Dragoni G , Scaringi S , Staderini F , Nannoni A , Ficari F , et al. Relationship between nutritional screening tools and glim in complicated IBD requiring surgery. Nutrients. 2021;13(11):1– 111. 10.3390/nu13113899 PMC8623109 34836154

[86] Jansen I , Prager M , Valentini L , Buning C . Inflammation‐driven malnutrition: a new screening tool predicts outcome in Crohn's disease. Br J Nutr. 2016;116(6):1061–7. 27546478 10.1017/S0007114516003044

[87] Taylor LM , Eslamparast T , Farhat K , Kroeker K , Halloran B , Shommu N , et al. Using patient completed screening tools to predict risk of malnutrition in patients with inflammatory bowel disease. Crohn's and Colitis. 2021;360(3):3. 10.1093/crocol/otab043 PMC9802362 36776646

[88] Harries AD , Jones LA , Danis V , Fifield R , Heatley RV , Newcombe RG , et al. Controlled trial of supplemented oral nutrition in Crohn's disease. Lancet. 1983;1(8330):887–90. 6132218 10.1016/s0140-6736(83)91325-9

[89] Verma S , Kirkwood B , Brown S , Giaffer MH . Oral nutritional supplementation is effective in the maintenance of remission in Crohn's disease. Dig Liver Dis. 2000;32(9):769–74. 11215556 10.1016/s1590-8658(00)80353-9

[90] Sökülmez P , Demirbag AE , Arslan P , Disibeyaz S . Effects of enteral nutritional support on malnourished patients with inflammatory bowel disease by subjective global assessment. Turk J Gastroenterol. 2014;25(5):493–507. 25417609 10.5152/tjg.2014.4955

[91] Baldwin C , Weekes CE . Dietary advice with or without oral nutritional supplements for disease‐related malnutrition in adults. Cochrane Database Syst Rev. 2011;9:CD002008. 10.1002/14651858.CD002008.pub4 PMC6465043 21901680

[92] Narula N , Dhillon A , Zhang D , Sherlock ME , Tondeur M , Zachos M . Enteral nutritional therapy for induction of remission in Crohn's disease. Cochrane Database Syst Rev. 2018;4:CD000542. 29607496 10.1002/14651858.CD000542.pub3 PMC6494406

[93] Gassull MA , Fernández‐Bañares F , Cabré E , Papo M , Giaffer MH , Sánchez‐Lombraña JL , et al. Fat composition may be a clue to explain the primary therapeutic effect of enteral nutrition in Crohn's disease: results of a double blind randomised multicentre European trial. Gut. 2002;51(2):164–8. 12117873 10.1136/gut.51.2.164 PMC1773299

[94] González‐Huix F , de León R , Fernández‐Bañares F , Esteve M , Cabré E , Acero D , et al. Polymeric enteral diets as primary treatment of active Crohn's disease: a prospective steroid controlled trial. Gut. 1993;34(6):778–82. 8314510 10.1136/gut.34.6.778 PMC1374261

[95] Lindor KD , Fleming CR , Burnes JU , Nelson JK , Ilstrup DM . A randomized prospective trial comparing a defined formula diet, corticosteroids, and a defined formula diet plus corticosteroids in active Crohn's disease. Mayo Clin Proc. 1992;67(4):328–33. 1548947 10.1016/s0025-6196(12)61547-x

[96] Lochs H , Steinhardt HJ , Klaus‐Wentz B , Zeitz M , Vogelsang H , Sommer H , et al. Comparison of enteral nutrition and drug treatment in active Crohn's disease. Results of the European Cooperative Crohn's Disease Study. IV. Gastroenterology. 1991;101(4):881–8. 1679736 10.1016/0016-5085(91)90711-s

[97] Saverymuttu S , Hodgson HJ , Chadwick VS . Controlled trial comparing prednisolone with an elemental diet plus non‐absorbable antibiotics in active Crohn's disease. Gut. 1985;26(10):994–8. 3902590 10.1136/gut.26.10.994 PMC1432937

[98] Malchow H , Steinhardt HJ , Lorenz‐Meyer H , Strohm WD , Rasmussen S , Sommer H , et al. Feasibility and effectiveness of a defined‐formula diet regimen in treating active Crohn's disease. European Cooperative Crohn's Disease Study III. Scand J Gastroenterol. 1990;25(3):235–44. 1969678

[99] Borrelli O , Cordischi L , Cirulli M , Paganelli M , Labalestra V , Uccini S , et al. Polymeric diet alone versus corticosteroids in the treatment of active pediatric Crohn's disease: a randomized controlled open‐label trial. Clin Gastroenterol Hepatol. 2006;4(6):744–53. 16682258 10.1016/j.cgh.2006.03.010

[100] Terrin G , Canani RB , Ambrosini A , Viola F , De Mesquita MB , Di , et al. A semielemental diet (Pregomin) as primary therapy for inducing remission in children with active Crohn's disease. Ital J Pediatr. 2002;28(5):401–5.

[101] Gomollón F , Dignass A , Annese V , Tilg H , Van Assche G , Lindsay JO , et al. European Evidence‐based Consensus on the Diagnosis and Management of Crohn's Disease 2016: Part 1: Diagnosis and Medical Management. J Crohns Colitis. 2017;11(1):3–25. 27660341 10.1093/ecco-jcc/jjw168

[102] Pfeffer‐Gik T , Yanai H , Godny L , Ron Y , Maharshak N , Dotan I . Exclusive enteral nutrition in adults with active Crohn's disease is associated with decrease in disease activity. United European Gastroenterol J. 2016;4(5 Suppl 1):A270.

[103] Wall CL , Gearry RB , Day AS . Treatment of active Crohn's disease with exclusive and partial enteral nutrition: a pilot study in adults. Inflamm Intest Dis. 2018;2(4):219–27. 30221149 10.1159/000489630 PMC6135224

[104] Sahu P , Kedia S , Vuyyuru SK , Bajaj A , Markandey M , Singh N , et al. Randomised clinical trial: exclusive enteral nutrition versus standard of care for acute severe ulcerative colitis. Aliment Pharmacol Ther. 2021;53(5):568–76. 33440046 10.1111/apt.16249

[105] Ashton JJ , Gavin J , Beattie RM . Exclusive enteral nutrition in Crohn's disease: evidence and practicalities. Clin Nutr. 2019;38(1):80–9. 29398336 10.1016/j.clnu.2018.01.020

[106] Gong J , Zuo L , Guo Z , Zhang L , Li Y , Gu L , et al. Impact of disease activity on resting energy expenditure and body composition in adult Crohn's disease: a prospective longitudinal assessment. J Parenter Enteral Nutr. 2015;39(6):713–8. 10.1177/0148607114528360 24668997

[107] Brotherton CS , Taylor AG , Bourguignon C , Anderson JG . A high‐fiber diet may improve bowel function and health‐related quality of life in patients with Crohn disease. Gastroenterol Nurs. 2014;37(3):206–16. 24871666 10.1097/SGA.0000000000000047 PMC4260718

[108] Bradbury KE , Murphy N , Key TJ . Diet and colorectal cancer in UK Biobank: a prospective study. Int J Epidemiol. 2020;49(1):246–58. 30993317 10.1093/ije/dyz064 PMC7124508

[109] Svolos V , Hansen R , Nichols B , Quince C , Ijaz UZ , Papadopoulou RT , et al. Treatment of active Crohn's disease with an ordinary food‐based diet that replicates exclusive enteral nutrition. Gastroenterology. 2019;156(5):1354–67e6. 30550821 10.1053/j.gastro.2018.12.002

[110] Svolos V , Hansen R , Russell R , Gaya DR , John Paul S , Macdonald J , et al. CD‐TREAT diet induces remission and improves quality of life in an open label trial in children and adults with active Crohn's disease. J Crohns Colitis. 2022;16(Suppl 1):i112.

[111] Levine A , Wine E , Assa A , Sigall Boneh R , Shaoul R , Kori M , et al. Crohn's disease exclusion diet plus partial enteral nutrition induces sustained remission in a randomized controlled trial. Gastroenterology. 2019;157(2):440–50e8. 31170412 10.1053/j.gastro.2019.04.021

[112] Yanai H , Levine A , Hirsch A , Boneh RS , Kopylov U , Eran HB , et al. The Crohn's disease exclusion diet for induction and maintenance of remission in adults with mild‐to‐moderate Crohn's disease (CDED‐AD): an open‐label, pilot, randomised trial. Lancet Gastroenterol Hepatol. 2022;7(1):49–59. 34739863 10.1016/S2468-1253(21)00299-5

[113] Sandall AM , Cox SR , Lindsay JO , Gewirtz AT , Chassaing B , Rossi M , et al. Emulsifiers impact colonic length in mice and emulsifier restriction is feasible in people with Crohn's disease. Nutrients. 2020;12(9):837‐ 852. 32942699 10.3390/nu12092827 PMC7551245

[114] Limketkai BN , Iheozor‐Ejiofor Z , Gjuladin‐Hellon T , Parian A , Matarese LE , Bracewell K , et al. Dietary interventions for induction and maintenance of remission in inflammatory bowel disease. Cochrane Database Syst Rev. 2019;2:CD012839. 30736095 10.1002/14651858.CD012839.pub2 PMC6368443

[115] Dariel I , Levi Z , Fraser A , Hadad B , Niv Y , Fraser G . Elimination diets in the treatment of mildly active Crohn's disease – results of a randomized controlled trial. Gastroenterology. 2007;132(4):A507–8.

[116] Brotherton CS , Taylor AG , Bourguignon C , Anderson JG . A high‐fiber diet may improve bowel function and health‐related quality of life in patients with Crohn disease. Gastroenterol Nurs. 2014;37(3):206–16. 24871666 10.1097/SGA.0000000000000047 PMC4260718

[117] Levenstein S , Prantera C , Luzi C , D'Ubaldi A . Low residue or normal diet in Crohn's disease: a prospective controlled study in Italian patients. Gut. 1985;26(10):989–93. 2996991 10.1136/gut.26.10.989 PMC1432953

[118] Sigall‐Boneh R , Pfeffer‐Gik T , Segal I , Zangen T , Boaz M , Levine A . Partial enteral nutrition with a Crohn's disease exclusion diet is effective for induction of remission in children and young adults with Crohn's disease. Inflamm Bowel Dis. 2014;20(8):1353–60. 24983973 10.1097/MIB.0000000000000110

[119] Olendzki BC , Silverstein TD , Persuitte GM , Ma YS , Baldwin KR , Cave D . An anti‐inflammatory diet as treatment for inflammatory bowel disease: a case series report. Nutr J. 2014;13(5):5. 24428901 10.1186/1475-2891-13-5 PMC3896778

[120] Pedersen N , Ankersen DV , Felding M , Wachmann H , Végh Z , Molzen L , et al. Low‐FODMAP diet reduces irritable bowel symptoms in patients with inflammatory bowel disease. World J Gastroenterol. 2017;23(18):3356–66. 28566897 10.3748/wjg.v23.i18.3356 PMC5434443

[121] Cox SR , Lindsay JO , Fromentin S , Stagg AJ , McCarthy NE , Galleron N , et al. Effects of low‐FODMAP Diet on symptoms, fecal microbiome, and markers of inflammation in patients with quiescent inflammatory bowel disease in a randomized trial. Gastroenterology. 2019;158:176–88. 31586453 10.1053/j.gastro.2019.09.024

[122] Lewis JD , Sandler RS , Brotherton C , Brensinger C , Li H , Kappelman MD , et al. A randomized trial comparing the specific carbohydrate diet to a Mediterranean diet in adults with Crohn's disease. Gastroenterology. 2021;161(3):837–52. 34052278 10.1053/j.gastro.2021.05.047 PMC8396394

[123] Gunasekeera V , Mendall MA , Chan D , Kumar D . Treatment of Crohn's disease with an IgG4‐guided exclusion diet: a randomized controlled trial. Dig Dis Sci. 2016;61(4):1148–57. 26809868 10.1007/s10620-015-3987-z

[124] Bartel G , Weiss I , Turetschek K , Schima W , Püspök A , Waldhoer T , et al. Ingested matter affects intestinal lesions in Crohn's disease. Inflamm Bowel Dis. 2008;14(3):374–82. 17932967 10.1002/ibd.20295

[125] Albenberg L , Brensinger CM , Wu Q , Gilroy E , Kappelman MD , Sandler RS , et al. A diet low in red and processed meat does not reduce rate of Crohn's disease flares. Gastroenterology. 2019;157(1):128–36. 30872105 10.1053/j.gastro.2019.03.015 PMC6726378

[126] Lomer MC , Grainger SL , Ede R , Catterall AP , Greenfield SM , Cowan RE , et al. Lack of efficacy of a reduced microparticle diet in a multi‐centred trial of patients with active Crohn's disease. Eur J Gastroenterol Hepatol. 2005;17(3):377–84. 15716665 10.1097/00042737-200503000-00019

[127] Lomer MC , Harvey RS , Evans SM , Thompson RP , Powell JJ . Efficacy and tolerability of a low microparticle diet in a double blind, randomized, pilot study in Crohn's disease. Eur J Gastroenterol Hepatol. 2001;13(2):101–6. 11246607 10.1097/00042737-200102000-00003

[128] Chiba M , Tsuji T , Nakane K , Tsuda S , Ishii H , Ohno H , et al. Induction with infliximab and a plant‐based diet as first‐line (IPF) therapy for Crohn disease: a single‐group trial. Perm J. 2017;21:17‐009. 10.7812/TPP/17-009 PMC5638637 29035182

[129] Wedlake L , Slack N , Andreyev HJ , Whelan K . Fiber in the treatment and maintenance of inflammatory bowel disease: a systematic review of randomized controlled trials. Inflamm Bowel Dis. 2014;20(3):576–86. 24445775 10.1097/01.MIB.0000437984.92565.31

[130] Peters V , Dijkstra G , Campmans‐Kuijpers MJE . Are all dietary fibers equal for patients with inflammatory bowel disease? A systematic review of randomized controlled trials. Nutr Res. 2021;6:1375‐ 1382. 10.1093/nutrit/nuab062 PMC8990763 34486663

[131] Furrie E , Macfarlane S , Kennedy A , Cummings JH , Walsh SV , O'Neil DA , et al. Synbiotic therapy (*Bifidobacterium longum*/Synergy 1) initiates resolution of inflammation in patients with active ulcerative colitis: a randomised controlled pilot trial. Gut. 2005;54(2):242–9. 15647189 10.1136/gut.2004.044834 PMC1774839

[132] Casellas F , Borruel N , Torrejón A , Varela E , Antolin M , Guarner F , et al. Oral oligofructose‐enriched inulin supplementation in acute ulcerative colitis is well tolerated and associated with lowered faecal calprotectin. Aliment Pharmacol Ther. 2007;25(9):1061–7. 17439507 10.1111/j.1365-2036.2007.03288.x

[133] Ishikawa H , Matsumoto S , Ohashi Y , Imaoka A , Setoyama H , Umesaki Y , et al. Beneficial effects of probiotic bifidobacterium and galacto‐oligosaccharide in patients with ulcerative colitis: a randomized controlled study. Digestion. 2011;84(2):128–33. 21525768 10.1159/000322977

[134] Kanauchi O , Suga T , Tochihara M , Hibi T , Naganuma M , Homma T , et al. Treatment of ulcerative colitis by feeding with germinated barley foodstuff: first report of a multicenter open control trial. J Gastroenterol. 2002;37(Suppl 14):67–72. 12572869 10.1007/BF03326417

[135] Federico A , Tuccillo C , Grossi E , Abbiati R , Garbagna N , Romano M , et al. The effect of a new symbiotic formulation on plasma levels and peripheral blood mononuclear cell expression of some pro‐inflammatory cytokines in patients with ulcerative colitis: a pilot study. Eur Rev Med Pharmacol Sci. 2009;13(4):285–93. 19694343

[136] Benjamin JL , Hedin CR , Koutsoumpas A , Ng SC , McCarthy NE , Hart AL , et al. Randomised, double‐blind, placebo‐controlled trial of fructo‐oligosaccharides in active Crohn's disease. Gut. 2011;60(7):923–9. 21262918 10.1136/gut.2010.232025

[137] Steed H , Macfarlane GT , Blackett KL , Bahrami B , Reynolds N , Walsh SV , et al. Clinical trial: the microbiological and immunological effects of synbiotic consumption – a randomized double‐blind placebo‐controlled study in active Crohn's disease. Aliment Pharmacol Ther. 2010;32(7):872–83. 20735782 10.1111/j.1365-2036.2010.04417.x

[138] Ahamed Z R , Dutta U , Sharma V , Prasad KK , Popli P , Kalsi D , et al. Oral nano vitamin D supplementation reduces disease activity in ulcerative colitis: a double‐blind randomized parallel group placebo‐controlled trial. J Clin Gastroenterol. 2019;53(10):e409–e15. 31356558 10.1097/MCG.0000000000001233

[139] Garg M , Rosella O , Rosella G , Wu Y , Lubel JS , Gibson PR . Evaluation of a 12‐week targeted vitamin D supplementation regimen in patients with active inflammatory bowel disease. Clin Nutr. 2018;37(4):1375–82. 28651829 10.1016/j.clnu.2017.06.011

[140] Seidner DL , Lashner BA , Brzezinski A , Banks PL , Goldblum J , Fiocchi C , et al. An oral supplement enriched with fish oil, soluble fiber, and antioxidants for corticosteroid sparing in ulcerative colitis: a randomized, controlled trial. Clin Gastroenterol Hepatol. 2005;3(4):358–69. 15822041 10.1016/s1542-3565(04)00672-x

[141] Wiese DM , Lashner BA , Lerner E , DeMichele SJ , Seidner DL . The effects of an oral supplement enriched with fish oil, prebiotics, and antioxidants on nutrition status in Crohn's disease patients. Nutr Clin Pract. 2011;26(4):463–73. 21775642 10.1177/0884533611413778 PMC4457393

[142] Langhorst J , Wulfert H , Lauche R , Klose P , Cramer H , Dobos GJ , et al. Systematic review of complementary and alternative medicine treatments in inflammatory bowel diseases. J Crohns Colitis. 2015;9(1):86–106. 25518050 10.1093/ecco-jcc/jju007

[143] Kedia S , Bhatia V , Thareja S , Garg S , Mouli VP , Bopanna S , et al. Low dose oral curcumin is not effective in induction of remission in mild to moderate ulcerative colitis: results from a randomized double blind placebo controlled trial. World J Gastrointest Pharmacol Ther. 2017;8(2):147–54. 28533925 10.4292/wjgpt.v8.i2.147 PMC5421114

[144] Masoodi M , Mahdiabadi MA , Mokhtare M , Agah S , Kashani AHF , Rezadoost AM , et al. The efficacy of curcuminoids in improvement of ulcerative colitis symptoms and patients’ self‐reported well‐being: a randomized double‐blind controlled trial. J Cell Biochem. 2018;119(11):9552–9. 30132960 10.1002/jcb.27273

[145] Sadeghi N , Mansoori A , Shayesteh A , Hashemi SJ . The effect of curcumin supplementation on clinical outcomes and inflammatory markers in patients with ulcerative colitis. Phytother Res. 2020;34(5):1123–33. 31802559 10.1002/ptr.6581

[146] Banerjee R , Pal P , Penmetsa A , Kathi P , Girish G , Goren I , et al. Novel bioenhanced curcumin with mesalamine for induction of clinical and endoscopic remission in mild‐to‐moderate ulcerative colitis: a randomized double‐blind placebo‐controlled pilot study. J Clin Gastroenterol. 2021;55(8):702–8. 32889959 10.1097/MCG.0000000000001416

[147] Lang A , Salomon N , Wu JC , Kopylov U , Lahat A , Har‐Noy O , et al. Curcumin in combination with mesalamine induces remission in patients with mild‐to‐moderate ulcerative colitis in a randomized controlled trial. Clin Gastroenterol Hepatol. 2015;13(8):1444–9. 25724700 10.1016/j.cgh.2015.02.019

[148] Singla V , Pratap Mouli V , Garg SK , Rai T , Choudhury BN , Verma P , et al. Induction with NCB‐02 (curcumin) enema for mild‐to‐moderate distal ulcerative colitis – a randomized, placebo‐controlled, pilot study. J Crohns Colitis. 2014;8(3):208–14. 24011514 10.1016/j.crohns.2013.08.006

[149] Sugimoto K , Ikeya K , Bamba S , Andoh A , Yamasaki H , Mitsuyama K , et al. Highly bioavailable curcumin derivative ameliorates Crohn's disease symptoms: a randomized, double‐blind, multicenter study. J Crohns Colitis. 2020;14:1693–1701. 32412598 10.1093/ecco-jcc/jjaa097

[150] Irving PM , Iqbal T , Nwokolo C , Subramanian S , Bloom S , Prasad N , et al. A randomized, double‐blind, placebo‐controlled, parallel‐group, pilot study of cannabidiol‐rich botanical extract in the symptomatic treatment of ulcerative colitis. Inflamm Bowel Dis. 2018;24(4):714–24. 29538683 10.1093/ibd/izy002

[151] Naftali T , Bar‐Lev Schleider L , Scklerovsky Benjaminov F , Konikoff FM , Matalon ST , Ringel Y . Cannabis is associated with clinical but not endoscopic remission in ulcerative colitis: a randomized controlled trial. PLoS ONE [Electronic Resource]. 2021;16(2):e0246871. 33571293 10.1371/journal.pone.0246871 PMC7877751

[152] Torres J , Ellul P , Langhorst J , Mikocka‐Walus A Barreiro‐de Acosta M , Basnayake C , et al. European Crohn's and colitis organisation topical review on complementary medicine and psychotherapy in inflammatory bowel disease. J Crohns Colitis. 2019;13(6):673–85e. 30820529 10.1093/ecco-jcc/jjz051

[153] Banerjee R , Penmetsa A , Medaboina K , Boramma GG , Amsrala S , Reddy DN . Novel bio‐enhanced curcumin with mesalamine for induction of remission in mild to moderate ulcerative colitis. Gastroenterology. 2017;152(5):S587–S.

[154] Chen M , Feng Y , Liu W . Efficacy and safety of probiotics in the induction and maintenance of inflammatory bowel disease remission: a systematic review and meta‐analysis. Ann Palliat Med. 2021;10(11):11821–9. 34872306 10.21037/apm-21-2996

[155] Zhang XF , Guan XX , Tang YJ , Sun JF , Wang XK , Wang WD , et al. Clinical effects and gut microbiota changes of using probiotics, prebiotics or synbiotics in inflammatory bowel disease: a systematic review and meta‐analysis. Eur J Nutr. 2021;60(5):2855–75. 33555375 10.1007/s00394-021-02503-5

[156] Kaur L , Gordon M , Baines PA , Iheozor‐Ejiofor Z , Sinopoulou V , Akobeng AK . Probiotics for induction of remission in ulcerative colitis. Cochrane Database Syst Rev. 2020;3:CD005573. 32128795 10.1002/14651858.CD005573.pub3 PMC7059959

[157] Limketkai BN , Akobeng AK , Gordon M , Adepoju AA . Probiotics for induction of remission in Crohn's disease. Cochrane Database Syst Rev. 2020;7:CD006634. 32678465 10.1002/14651858.CD006634.pub3 PMC7389339

[158] Pabon‐Carrasco M , Ramirez‐Baena L , Vilar‐Palomo S , Castro‐Mendez A , Martos‐Garcia R , Rodriguez‐Gallego I . Probiotics as a coadjuvant factor in active or quiescent inflammatory bowel disease of adults – a meta‐analytical study. Nutrients. 2020;12(9):2218– 2227. 32872272 10.3390/nu12092628 PMC7551006

[159] Derwa Y , Gracie DJ , Hamlin PJ , Ford AC . Systematic review with meta‐analysis: the efficacy of probiotics in inflammatory bowel disease. Aliment Pharmacol Ther. 2017;46(4):389–400. 28653751 10.1111/apt.14203

[160] Dang X , Xu M , Liu D , Zhou D , Yang W . Assessing the efficacy and safety of fecal microbiota transplantation and probiotic VSL#3 for active ulcerative colitis: a systematic review and meta‐analysis. PLoS One. 2020;15(3):e0228846. 32182248 10.1371/journal.pone.0228846 PMC7077802

[161] Ghouri YA , Richards DM , Rahimi EF , Krill JT , Jelinek KA , DuPont AW . Systematic review of randomized controlled trials of probiotics, prebiotics, and synbiotics in inflammatory bowel disease. Clin Exp Gastroenterol. 2014;7:473–87. 25525379 10.2147/CEG.S27530 PMC4266241

[162] Chen P , Xu H , Tang H , Zhao F , Yang C , Kwok LY , et al. Modulation of gut mucosal microbiota as a mechanism of probiotics‐based adjunctive therapy for ulcerative colitis. Microb Biotechnol. 2020;13(6):2032–43. 32969200 10.1111/1751-7915.13661 PMC7533322

[163] Petersen AM , Mirsepasi H , Halkjaer SI , Mortensen EM , Nordgaard‐Lassen I , Krogfelt KA . Ciprofloxacin and probiotic *Escherichia coli* Nissle add‐on treatment in active ulcerative colitis: a double‐blind randomized placebo controlled clinical trial. J Crohn Colitis. 2014;8(11):1498–505. 10.1016/j.crohns.2014.06.001 24972748

[164] Kato K , Mizuno S , Umesaki Y , Ishii Y , Sugitani M , Imaoka A , et al. Randomized placebo‐controlled trial assessing the effect of bifidobacteria‐fermented milk on active ulcerative colitis. Aliment Pharmacol Ther. 2004;20(10):1133–41. 15569116 10.1111/j.1365-2036.2004.02268.x

[165] Matthes H , Krummenerl T , Giensch M , Wolff C , Schulze J . Clinical trial: probiotic treatment of acute distal ulcerative colitis with rectally administered *Escherichia coli* Nissle 1917 (EcN). BMC Complement Altern Med. 2010;10:13. 20398311 10.1186/1472-6882-10-13 PMC2861635

[166] Ng SC , Plamondon S , Kamm MA , Hart AL , Al‐Hassi HO , Guenther T , et al. Immunosuppressive effects via human intestinal dendritic cells of probiotic bacteria and steroids in the treatment of acute ulcerative colitis. Inflamm Bowel Dis. 2010;16(8):1286–98. 20155842 10.1002/ibd.21222

[167] Tursi A , Brandimarte G , Papa A , Giglio A , Elisei W , Giorgetti GM , et al. Treatment of relapsing mild‐to‐moderate ulcerative colitis with the probiotic VSL3 as adjunctive to a standard pharmaceutical treatment: a double‐blind, randomized, placebo‐controlled study. Am J Gastroenterol. 2010;105(10):2218–27. 20517305 10.1038/ajg.2010.218 PMC3180711

[168] Krag A , Munkholm P , Israelsen H , Von Ryberg B , Andersen KK , Bendtsen F . Profermin is efficacious in patients with active ulcerative colitis – a randomized controlled trial. Inflamm Bowel Dis. 2013;19(12):2584–92. 24108114 10.1097/01.MIB.0000437046.26036.db

[169] Sood A , Midha V , Makharia GK , Ahuja V , Singal D , Goswami P , et al. The probiotic preparation, VSL#3 induces remission in patients with mild‐to‐moderately active ulcerative colitis. Clin Gastroenterol Hepatol. 2009;7(11):1202–9. 19631292 10.1016/j.cgh.2009.07.016

[170] Tamaki H , Nakase H , Inoue S , Kawanami C , Itani T , Ohana M , et al. Efficacy of probiotic treatment with Bifidobacterium longum 536 for induction of remission in active ulcerative colitis: a randomized, double‐blinded, placebo‐controlled multicenter trial. Dig Endosc. 2016;28(1):67–74. 26418574 10.1111/den.12553

[171] Vejdani R , Bahari A , Zadeh AM , Azmi M , Ebrahimi‐Daryani N , Hashtroudi AA , et al. Effects of *Lactobacillus casei* probiotic on mild to moderate ulcerative colitis: a placebo controlled study. Indian J Med Sci. 2017;69(1):24–8.

[172] Cui HH , Chen CL , Wang JD , Yang YJ , Cun Y , Wu JB , et al. Effects of probiotic on intestinal mucosa of patients with ulcerative colitis. World J Gastroenterol. 2004;10(10):1521–5. 15133865 10.3748/wjg.v10.i10.1521 PMC4656296

[173] Gionchetti P , Rizzello F , Venturi A , Brigidi P , Matteuzzi D , Bazzocchi G , et al. Oral bacteriotherapy as maintenance treatment in patients with chronic pouchitis: a double‐blind, placebo‐controlled trial. Gastroenterology. 2000;119(2):305–9. 10930365 10.1053/gast.2000.9370

[174] Gionchetti P , Rizzello F , Helwig U , Venturi A , Lammers KM , Brigidi P , et al. Prophylaxis of pouchitis onset with probiotic therapy: a double‐blind, placebo‐controlled trial. Gastroenterology. 2003;124(5):1202–9. 12730861 10.1016/s0016-5085(03)00171-9

[175] Mimura T , Rizzello F , Helwig U , Poggioli G , Schreiber S , Talbot IC , et al. Once daily high dose probiotic therapy (VSL#3) for maintaining remission in recurrent or refractory pouchitis. Gut. 2004;53(1):108–14. 14684584 10.1136/gut.53.1.108 PMC1773918

[176] Pronio A , Montesani C , Butteroni C , Vecchione S , Mumolo G , Vestri A , et al. Probiotic administration in patients with ileal pouch‐anal anastomosis for ulcerative colitis is associated with expansion of mucosal regulatory cells. Inflamm Bowel Dis. 2008;14(5):662–8. 18240282 10.1002/ibd.20369

[177] Brown SJ , Megan J , Smith S , Matchet D , Elliott R . *Bifidobacterium longum* BB‐536 and prevention of acute pouchitis. Gastroenterology. 2004;126(4 Suppl 2):S265.

[178] Plein K , Hotz J . Therapeutic effects of *Saccharomyces boulardii* on mild residual symptoms in a stable phase of Crohn's disease with special respect to chronic diarrhea – a pilot study. Z Gastroenterol. 1993;31(2):129–34. 8465554

[179] Schultz M , Timmer A , Herfarth HH , Sartor RB , Vanderhoof JA , Rath HC , et al. in inducing and maintaining remission of Crohn's disease. BMC Gastroenterol. 2004;4:5. 15113451 10.1186/1471-230X-4-5 PMC394324

[180] Malchow HA . Crohn's disease and *Escherichia coli*. A new approach in therapy to maintain remission of colonic Crohn's disease? J Clin Gastroenterol. 1997;25(4):653–8. 9451682 10.1097/00004836-199712000-00021

[181] Wang G , Ren J , Li G , Hu Q , Gu G , Ren H , et al. The utility of food antigen test in the diagnosis of Crohn's disease and remission maintenance after exclusive enteral nutrition. Clin Res Hepatol Gastroenterol. 2018;42(2):145–52. 29102418 10.1016/j.clinre.2017.09.002

[182] Damas OM , Garces L , Abreu MT . Diet as adjunctive treatment for inflammatory bowel disease: review and update of the latest literature. Curr Treat Options Gastroenterol. 2019;17(2):313–25. 30968340 10.1007/s11938-019-00231-8 PMC6857843

[183] Keshteli AH , Valcheva R , Nickurak C , Halloran BP , Van Zanten SV , Kroeker K , et al. Adherence to an “anti‐inflammatory diet” for 6 months can decrease fecal calprotectin in ulcerative colitis patients: preliminary findings of a randomized controlled trial. Gastroenterology. 2016;1(4):S807–S8.

[184] Hanai H , Iida T , Takeuchi K , Arai H , Arai O , Abe J , et al. Nutritional therapy versus 6‐mercaptopurine as maintenance therapy in patients with Crohn's disease. Dig Liver Dis. 2012;44(8):649–54. 22542605 10.1016/j.dld.2012.03.007

[185] Takagi S , Utsunomiya K , Kuriyama S , Yokoyama H , Takahashi S , Iwabuchi M , et al. Effectiveness of an 'half elemental diet' as maintenance therapy for Crohn's disease: a randomized‐controlled trial. Aliment Pharmacol Ther. 2006;24(9):1333–40. 17059514 10.1111/j.1365-2036.2006.03120.x

[186] Triantafillidis JK , Stamataki A , Karagianni V , Gikas A , Malgarinos G . Maintenance treatment of Crohn's disease with a polymeric feed rich in TGF‐beta. Ann Gastroenterol. 2010;23(2):113–8.

[187] Yamamoto T , Nakahigashi M , Shimoyama T , Matsumoto K . The long‐term efficacy of concomitant enteral nutritional therapy during maintenance infliximab in patients with Crohn's disease: a prospective observational trial. J Crohns Colitis. 2015;9:S364–S.

[188] Yoshimura N , Kawaguchi T , Sako M , Saniabadi A , Takazoe M . In patients with Crohn's disease, concomitant enteral nutrition reduces the loss of response to adalimumab maintenance therapy. Gastroenterology. 2014;146(5):S382–S.

[189] Sazuka S , Katsuno T , Nakagawa T , Saito M , Saito K , Matsumura T , et al. Concomitant use of enteral nutrition therapy is associated with sustained response to infliximab in patients with Crohn's disease. Eur J Clin Nutr. 2012;66(11):1219–23. 23010687 10.1038/ejcn.2012.120

[190] Hirai F , Ishida T , Takeshima F , Yamamoto S , Yoshikawa I , Ashizuka S , et al. Effect of a concomitant elemental diet with maintenance anti‐tumor necrosis factor‐alpha antibody therapy in patients with Crohn's disease: a multicenter, prospective cohort study. J Gastroenterol Hepatol. 2019;34(1):132–9. 29935082 10.1111/jgh.14361 PMC7379489

[191] Yamamoto T , Nakahigashi M , Umegae S , Matsumoto K . Prospective clinical trial: enteral nutrition during maintenance infliximab in Crohn's disease. J Gastroenterol. 2010;45(1):24–9. 19798465 10.1007/s00535-009-0136-5

[192] Sugita N , Watanabe K , Kamata N , Yukawa T , Otani K , Hosomi S , et al. Efficacy of a concomitant elemental diet to reduce the loss of response to adalimumab in patients with intractable Crohn's disease. J Gastroenterol Hepatol. 2018;33(3):631–7. 28857255 10.1111/jgh.13969

[193] Hirai F , Ishihara H , Yada S , Esaki M , Ohwan T , Nozaki R , et al. Effectiveness of concomitant enteral nutrition therapy and infliximab for maintenance treatment of Crohn's disease in adults. Dig Dis Sci. 2013;58(5):1329–34. 22926500 10.1007/s10620-012-2374-2 PMC3661072

[194] Hanai H , Iida T , Takeuchi K , Watanabe F , Maruyama Y , Andoh A , et al. Curcumin maintenance therapy for ulcerative colitis: randomized, multicenter, double‐blind, placebo‐controlled trial. Clin Gastroenterol Hepatol. 2006;4(12):1502–6. 17101300 10.1016/j.cgh.2006.08.008

[195] Bommelaer G , Laharie D , Nancey S , Hebuterne X , Roblin X , Nachury M , et al. Oral curcumin no more effective than placebo in preventing recurrence of Crohn's disease after surgery in a randomized controlled trial. Clin Gastroenterol Hepatol. 2020;18(7):1553–60. 31470175 10.1016/j.cgh.2019.08.041

[196] Prince A , Whelan K , Moosa A , Lomer MC , Reidlinger DP . Nutritional problems in inflammatory bowel disease: the patient perspective. J Crohns Colitis. 2011;5(5):443–50. 21939918 10.1016/j.crohns.2011.04.016

[197] Bischoff SC , Escher J , Hébuterne X , Kłęk S , Krznaric Z , Schneider S , et al. ESPEN practical guideline: clinical nutrition in inflammatory bowel disease. Clin Nutr. 2020;39(3):632–53. 32029281 10.1016/j.clnu.2019.11.002

[198] Fernández‐Bañares F , Hinojosa J , Sánchez‐Lombraña JL , Navarro E , Martínez‐Salmerón JF , García‐Pugés A , et al. Randomized clinical trial of Plantago ovata seeds (dietary fiber) as compared with mesalamine in maintaining remission in ulcerative colitis. Spanish Group for the Study of Crohn's Disease and Ulcerative Colitis (GETECCU). Am J Gastroenterol. 1999;94(2):427–33. 10022641 10.1111/j.1572-0241.1999.872_a.x

[199] Hallert C , Kaldma M , Petersson BG . Ispaghula husk may relieve gastrointestinal symptoms in ulcerative colitis in remission. Scand J Gastroenterol. 1991;26(7):747–50. 1654592 10.3109/00365529108998594

[200] Copaci I , Chiriac G . Maintenance of remission of ulcerative colitis: prebiotics and dietary fiber. United European Gastroenterol J. 2014;1:A375.

[201] Faghfoori Z , Navai L , Shakerhosseini R , Somi MH , Nikniaz Z , Norouzi MF . Effects of an oral supplementation of germinated barley foodstuff on serum tumour necrosis factor‐alpha, interleukin‐6 and ‐8 in patients with ulcerative colitis. Ann Clin Biochem. 2011;48(Pt 3):233–7. 21367884 10.1258/acb.2010.010093

[202] James SL , Christophersen CT , Bird AR , Conlon MA , Rosella O , Gibson PR , et al. Abnormal fibre usage in UC in remission. Gut. 2015;64(4):562–70. 25037189 10.1136/gutjnl-2014-307198

[203] Chermesh I , Tamir A , Reshef R , Chowers Y , Suissa A , Katz D , et al. Failure of synbiotic 2000 to prevent postoperative recurrence of Crohn's disease. Dig Dis Sci. 2007;52(2):385–9. 17211699 10.1007/s10620-006-9549-7

[204] Rutgeerts P , Feagan BG , Lichtenstein GR , Mayer LF , Schreiber S , Colombel JF , et al. Randomized placebo controlled trial of pro‐ and prebiotics (synbiotics cocktail) for maintenance of infliximab induced remission of luminal Crohn's disease (CD). Gastroenterology. 2004;126:A467–13.

[205] Jones VA , Dickinson RJ , Workman E . Crohn's disease: maintenance of remission by diet. Lancet. 1985;2(8448):177–14. 2862371 10.1016/s0140-6736(85)91497-7

[206] Ritchie JK , Wadsworth J , Lennard‐Jones JE , Rogers E . Controlled multicentre therapeutic trial of an unrefined carbohydrate, fibre rich diet in Crohn's disease. Br Med J. 1987;295(6597):517–20. 2822203 10.1136/bmj.295.6597.517 PMC1247426

[207] Valcheva R , Kovic O , Veniamin S , Perez‐Munoz ME , Silva M , Peerani F , et al. Prebiotic beta‐fructans prevent subclinical intestinal inflammation in ulcerative colitis patients who are in clinical remission. Gastroenterology. 2021;160(6 Suppl):S–120.

[208] Mutlu E , Mikolaitis S , Sedghi S , Chakradeo PS , Engen P , Chlipala G , et al. Dietary treatment of Crohn's disease: a randomized, placebo‐controlled, double‐blinded clinical trial. Gastroenterology. 2016;1(4):S778.

[209] Cox SR , Prince AC , Myers CE , Irving PM , Lindsay JO , Lomer MC , et al. Fermentable carbohydrates [FODMAPs] exacerbate functional gastrointestinal symptoms in patients with inflammatory bowel disease: a randomised, double‐blind, placebo‐controlled, cross‐over, re‐challenge trial. J Crohns Colitis. 2017;11(12):1420–9. 28525543 10.1093/ecco-jcc/jjx073

[210] Jørgensen SP , Agnholt J , Glerup H , Lyhne S , Villadsen GE , Hvas CL , et al. Clinical trial: vitamin D3 treatment in Crohn's disease – a randomized double‐blind placebo‐controlled study. Aliment Pharmacol Ther. 2010;32(3):377–83. 20491740 10.1111/j.1365-2036.2010.04355.x

[211] Narula N , Cooray M , Anglin R , Muqtadir Z , Narula A , Marshall JK . Impact of high‐dose vitamin D_3_ supplementation in patients with Crohn's disease in remission: a pilot randomized double‐blind controlled study. Dig Dis Sci. 2017;62(2):448–55. 27975236 10.1007/s10620-016-4396-7

[212] Iheozor‐Ejiofor Z , Kaur L , Gordon M , Baines PA , Sinopoulou V , Akobeng AK . Probiotics for maintenance of remission in ulcerative colitis. Cochrane Database Syst Rev. 2020;3:CD007443. 32128794 10.1002/14651858.CD007443.pub3 PMC7059960

[213] Bourreille A , Cadiot G , Le Dreau G , Laharie D , Beaugerie L , Dupas JL , et al. *Saccharomyces boulardii* does not prevent relapse of Crohn's disease. Clin Gastroenterol Hepatol. 2013;11(8):982–7. 23466709 10.1016/j.cgh.2013.02.021

[214] Bjarnason I , Sission G , Hayee B . A randomised, double‐blind, placebo‐controlled trial of a multi‐strain probiotic in patients with asymptomatic ulcerative colitis and Crohn's disease. Inflammopharmacology. 2019;27(3):465–73. 31054010 10.1007/s10787-019-00595-4 PMC6554453

[215] Willert RP , Peddi KK , Ombiga J , Bampton PA , Lawence IC . Randomised, double‐blinded, placebo‐controlled study of VSL#3 versus placebo in the maintenance of remission in Crohn's disease. Gastroenterology. 2010;138:S–517.

[216] Wildt S , Nordgaard I , Hansen U , Brockmann E , Rumessen JJ . A randomised double‐blind placebo‐controlled trial with *Lactobacillus acidophilus* La‐5 and *Bifidobacterium animalis* subsp. *lactis* BB‐12 for maintenance of remission in ulcerative colitis. J Crohns Colitis. 2011;5(2):115–21. 21453880 10.1016/j.crohns.2010.11.004

[217] Yoshimatsu Y , Yamada A , Furukawa R , Sono K , Osamura A , Nakamura K , et al. Effectiveness of probiotic therapy for the prevention of relapse in patients with inactive ulcerative colitis. World J Gastroenterol. 2015;21(19):5985–94. 26019464 10.3748/wjg.v21.i19.5985 PMC4438034

[218] Brennan GT , Ha I , Hogan C , Nguyen E , Jamal MM , Bechtold ML , et al. Does preoperative enteral or parenteral nutrition reduce postoperative complications in Crohn's disease patients: a meta‐analysis. Eur J Gastroenterol Hepatol. 2018;30(9):997–1002. 29738326 10.1097/MEG.0000000000001162

[219] Grass F , Pache B , Martin D , Hahnloser D , Demartines N , Hubner M . Preoperative nutritional conditioning of Crohn's patients – systematic review of current evidence and practice. Nutrients. 2017;9(6):790‐ 795. 28587182 10.3390/nu9060562 PMC5490541

[220] Rocha A , Bessa I , Lago P , Santos MD , Leite J , Castro‐Pocas F . Preoperative enteral nutrition and surgical outcomes in adults with Crohn's disease: a systematic review. GE Port J Gastroenterol. 2019;26(3):184–95. 31192287 10.1159/000494674 PMC6528105

[221] Gordon‐Dixon A , Gore‐Rodney J , Hampal R , Ross R , Miah A , Amorim Adegboye AR , et al. The role of exclusive enteral nutrition in the pre‐operative optimisation of adult patients with Crohn's disease. A systematic review. Clin Nutr ESPEN. 2021;46:99–105. 34857254 10.1016/j.clnesp.2021.09.723

[222] Heerasing N , Thompson B , Hendy P , Heap GA , Walker G , Bethune R , et al. Exclusive enteral nutrition provides an effective bridge to safer interval elective surgery for adults with Crohn's disease. Aliment Pharmacol Ther. 2017;45(5):660–9. 28105752 10.1111/apt.13934

[223] Wang H , Zuo L , Zhao J , Dong J , Li Y , Gu L , et al. Impact of preoperative exclusive enteral nutrition on postoperative complications and recurrence after bowel resection in patients with active Crohn's disease. World J Surg. 2016;40(8):1993–2000. 26940580 10.1007/s00268-016-3488-z

[224] Guo Z , Guo D , Gong J , Zhu W , Zuo L , Sun J , et al. Preoperative nutritional therapy reduces the risk of anastomotic leakage in patients with Crohn's disease requiring resections. Gastroenterol Res Pract. 2016;2016:5017856. 26858749 10.1155/2016/5017856 PMC4706910

[225] Li G , Ren J , Wang G , Hu D , Gu G , Liu S , et al. Preoperative exclusive enteral nutrition reduces the postoperative septic complications of fistulizing Crohn's disease. Eur J Clin Nutr. 2014;68(4):441–6. 24549026 10.1038/ejcn.2014.16

[226] Li Y , Zuo L , Zhu W , Gong J , Zhang W , Gu L , et al. Role of exclusive enteral nutrition in the preoperative optimization of patients with Crohn's disease following immunosuppressive therapy. Medicine. 2015;94(5):e478. 25654387 10.1097/MD.0000000000000478 PMC4602718

[227] Patel K , Sandall A , O'Hanlon D , Darakhshan A , Williams A , Westcott E , et al. Nutritional optimisation of pre‐surgical Crohn's disease patients with enteral nutrition significantly decreases length of stay and need for a stoma. Gut. 2016;65(Suppl 1):A47–A8.

[228] Zhu Y , Xu L , Liu W , Qi W , Cao Q , Zhou W . Safety and efficacy of exclusive enteral nutrition for percutaneously undrainable abdominal abscesses in Crohn's disease. Gastroenterol Res Pract. 2017;2017:6360319. 28947899 10.1155/2017/6360319 PMC5602481

[229] Zhang T , Cao L , Cao T , Yang J , Gong J , Zhu W , et al. Prevalence of sarcopenia and its impact on postoperative outcome in patients with Crohn's disease undergoing bowel resection. JPEN J Parenter Enteral Nutr. 2017;41(4):592–600. 26471990 10.1177/0148607115612054

[230] Yamamoto T , Nakahigashi M , Shimoyama T , Umegae S . Does preoperative enteral nutrition reduce the incidence of surgical complications in patients with Crohn's disease? A case‐matched study. Colorectal Dis. 2020;22(5):554–61. 31782874 10.1111/codi.14922

[231] Beaupel N , Brouquet A , Abdalla S , Carbonnel F , Penna C , Benoist S . Preoperative oral polymeric diet enriched with transforming growth factor‐beta 2 (Modulen) could decrease postoperative morbidity after surgery for complicated ileocolonic Crohn's disease. Scand J Gastroenterol. 2017;52(1):5–10. 27553420 10.1080/00365521.2016.1221994

[232] Meade S , Patel KV , Luber RP , O'Hanlon D , Caracostea A , Pavlidis P , et al. Pre‐operative oral enteral nutritional optimisation for Crohn's disease: a retrospective UK tertiary IBD centre cohort study. J Crohns Colitis 2022;16(Suppl 1):i545–i6. 10.1111/apt.17055 PMC9544188 35723622

[233] Hu D , Ren J , Wang G , Li G , Liu S , Yan D , et al. Exclusive enteral nutritional therapy can relieve inflammatory bowel stricture in Crohn's disease. J Clin Gastroenterol. 2014;48(9):790–5. 24440935 10.1097/MCG.0000000000000041

[234] Teahon K , Bjarnason I , Pearson M , Levi AJ . Ten years' experience with an elemental diet in the management of Crohn's disease. Gut. 1990;31(10):1133–7. 2083858 10.1136/gut.31.10.1133 PMC1378738

[235] Xie Y , Zhu WM , Li N , Li JS . Enteral nutritional therapy in Crohn disease complicated with incomplete intestinal obstruction. Zhonghua Wei Chang Wai Ke Za Zhi. 2010;13(12):891–4. 21186405

[236] Yang Q , Gao X , Chen H , Li M , Wu X , Zhi M , et al. Efficacy of exclusive enteral nutrition in complicated Crohn's disease. Scand J Gastroenterol. 2017;52(9):995–1001. 28598298 10.1080/00365521.2017.1335770

[237] Bengtsson J , Adlerberth I , Ostblom A , Saksena P , Oresland T , Borjesson L . Effect of probiotics (*Lactobacillus plantarum* 299 plus Bifidobacterium Cure21) in patients with poor ileal pouch function: a randomised controlled trial. Scand J Gastroenterol. 2016;51(9):1087–92. 27150635 10.3109/00365521.2016.1161067

[238] Kuisma J , Mentula S , Jarvinen H , Kahri A , Saxelin M , Farkkila M . Effect of *Lactobacillus rhamnosus* GG on ileal pouch inflammation and microbial flora. Aliment Pharmacol Ther. 2003;17(4):509–15. 12622759 10.1046/j.1365-2036.2003.01465.x

[239] de Oliveira AL , Boroni Moreira AP , Pereira Netto M , Goncalves , Leite IC . A cross‐sectional study of nutritional status, diet, and dietary restrictions among persons with an ileostomy or colostomy. Ostomy Wound Manage. 2018;64(5):18–29. 29847308

[240] Bingham S , Cummings JH , McNeil NI . Diet and health of people with an ileostomy. 1. Dietary assessment. Br J Nutr. 1982;47(3):399–406. 6282301 10.1079/bjn19820051

[241] Thomson TJ , Runcie J , Khan A . The effect of diet on ileostomy function. Gut. 1970;11(6):482–5. 5430373 10.1136/gut.11.6.482 PMC1553037

[242] Floruta CV . Dietary choices of people with ostomies. J Wound Ostomy Continence Nurs. 2001;28(1):28–31. 11174459 10.1067/mjw.2001.112079

[243] Akbulut G . Nutrition in stoma patients: a practical view of dietary therapy. Uhod‐Uluslar Hematol. 2011;21(1):61–6.

[244] Giunchi F , Cacciaguerra G , Borlotti ML , Pasini A , Giulianini G . Bowel movement and diet in patients with stomas. Br J Surg. 1988;75(7):722. 3416133 10.1002/bjs.1800750736

[245] Gazzard BG , Saunders B , Dawson AM . Diets and stoma function. Br J Surg. 1978;65(9):642–4. 698538 10.1002/bjs.1800650916

[246] Kramer P . Effect of specific foods, beverages, and spices on amount of ileostomy output in human subjects. Am J Gastroenterol. 1987;82(4):327–32. 3565337

[247] Barrett JS , Gearry RB , Muir JG , Irving PM , Rose R , Rosella O , et al. Dietary poorly absorbed, short‐chain carbohydrates increase delivery of water and fermentable substrates to the proximal colon. Aliment Pharmacol Ther. 2010;31(8):874–82. 20102355 10.1111/j.1365-2036.2010.04237.x

[248] Berghouse L , Hori S , Hill M , Hudson M , Lennard‐Jones JE , Rogers E . Comparison between the bacterial and oligosaccharide content of ileostomy effluent in subjects taking diets rich in refined or unrefined carbohydrate. Gut. 1984;25(10):1071–7. 6090279 10.1136/gut.25.10.1071 PMC1432535

[249] Crocetti D , Velluti F , La Torre V , Orsi E , De Anna L , La Torre F . Psyllium fiber food supplement in the management of stoma patients: results of a comparative prospective study. Tech Coloproctol. 2014;18(6):595–6. 23430350 10.1007/s10151-013-0983-1

[250] Yan D , Ren J , Wang G , Liu S , Li J . Predictors of response to enteral nutrition in abdominal enterocutaneous fistula patients with Crohn's disease. Eur J Clin Nutr. 2014;68(8):959–63. 24619104 10.1038/ejcn.2014.31

[251] Pironi L , Arends J , Bozzetti F , Cuerda C , Gillanders L , Jeppesen PB , et al. ESPEN guidelines on chronic intestinal failure in adults. Clin Nutr. 2016;35(2):247–307. 26944585 10.1016/j.clnu.2016.01.020

[252] Nightingale JM , Lennard‐Jones JE , Gertner DJ , Wood SR , Bartram CI . Colonic preservation reduces need for parenteral therapy, increases incidence of renal stones, but does not change high prevalence of gall stones in patients with a short bowel. Gut. 1992;33(11):1493–7. 1452074 10.1136/gut.33.11.1493 PMC1379534

[253] Crenn P , Morin MC , Joly F , Penven S , Thuillier F , Messing B . Net digestive absorption and adaptive hyperphagia in adult short bowel patients. Gut. 2004;53(9):1279–86. 15306586 10.1136/gut.2003.030601 PMC1774175

[254] Messing B , Pigot F , Rongier M , Morin MC , Ndeindoum U , Rambaud JC . Intestinal absorption of free oral hyperalimentation in the very short bowel syndrome. Gastroenterology. 1991;100(6):1502–8. 1850371 10.1016/0016-5085(91)90645-2

[255] Woolf GM , Miller C , Kurian R , Jeejeebhoy KN . Nutritional absorption in short bowel syndrome. Evaluation of fluid, calorie, and divalent cation requirements. Dig Dis Sci. 1987;32(1):8–15. 3792183 10.1007/BF01296681

[256] Bines JE . Intestinal failure: A new era in clinical management. J Gastroenterol Hepatol. 2009;24(Suppl 3):86–92S. 10.1111/j.1440-1746.2009.06077.x 19799705

[257] Nordgaard I , Hansen BS , Mortensen PB . Colon as a digestive organ in patients with short bowel. Lancet. 1994;343(8894):373–6. 7905549 10.1016/s0140-6736(94)91220-3

[258] Nordgaard I , Hansen BS , Mortensen PB . Importance of colonic support for energy absorption as small‐bowel failure proceeds. Am J Clin Nutr. 1996;64(2):222–31. 8694024 10.1093/ajcn/64.2.222

[259] Jeppesen PB , Mortensen PB . The influence of a preserved colon on the absorption of medium chain fat in patients with small bowel resection. Gut. 1998;43(4):478–83. 9824573 10.1136/gut.43.4.478 PMC1727269

[260] Kasidas GP , Rose GA . Oxalate content of some common foods: determination by an enzymatic method. J Hum Nutr. 1980;34(4):255–66. 7410821 10.3109/09637488009143446

[261] Marteau P , Messing B , Arrigoni E , Briet F , Flourié B , Morin MC , et al. Do patients with short‐bowel syndrome need a lactose‐free diet? Nutrition. 1997;13(1):13–6. 9058441 10.1016/s0899-9007(97)90872-8

[262] Arrigoni E , Marteau P , Briet F , Pochart P , Rambaud JC , Messing B . Tolerance and absorption of lactose from milk and yogurt during short‐bowel syndrome in humans. Am J Clin Nutr. 1994;60(6):926–9. 7985635 10.1093/ajcn/60.6.926

[263] Atia A , Girard‐Pipau F , Hébuterne X , Spies WG , Guardiola A , Ahn CW , et al. Macronutrient absorption characteristics in humans with short bowel syndrome and jejunocolonic anastomosis: starch is the most important carbohydrate substrate, although pectin supplementation may modestly enhance short chain fatty acid production and fluid absorption. JPEN J Parenter Enteral Nutr. 2011;35(2):229–40. 21378253 10.1177/0148607110378410

[264] Byrne TA , Morrissey TB , Nattakom TV , Ziegler TR , Wilmore DW . Growth hormone, glutamine, and a modified diet enhance nutrient absorption in patients with severe short bowel syndrome. JPEN J Parenter Enteral Nutr. 1995;19(4):296–302. 8523629 10.1177/0148607195019004296

[265] Grischkan D , Steiger E , Fazio V . Maintenance of home hyperalimentation in patients with high‐output jejunostomies. Arch Surg. 1979;114(7):838–41. 110288 10.1001/archsurg.1979.01370310080014

[266] Newton CR , Gonvers JJ , McIntyre PB , Preston DM , Lennard‐Jones JE . Effect of different drinks on fluid and electrolyte losses from a jejunostomy. J R Soc Med. 1985;78(1):27–34. 10.1177/014107688507800106 PMC1289541 3968667

[267] Nightingale JM , Lennard‐Jones JE . The short bowel syndrome: what's new and old? Dig Dis. 1993;11(1):12–31. 8443953 10.1159/000171397

[268] Vasant DH , Paine PA , Black CJ , Houghton LA , Everitt HA , Corsetti M , et al. British Society of Gastroenterology guidelines on the management of irritable bowel syndrome. Gut. 2021;70(7):1214–40. 33903147 10.1136/gutjnl-2021-324598

[269] McKenzie YA , Bowyer RK , Leach H , Gulia P , Horobin J , O'Sullivan NA , et al. British Dietetic Association systematic review and evidence‐based practice guidelines for the dietary management of irritable bowel syndrome in adults (2016 update). J Hum Nutr Diet. 2016;29(5):549–75. 27272325 10.1111/jhn.12385

[270] McKenzie YA , Thompson J , Gulia P , Lomer MC . British Dietetic Association systematic review of systematic reviews and evidence‐based practice guidelines for the use of probiotics in the management of irritable bowel syndrome in adults (2016 update). J Hum Nutr Diet. 2016;29(5):576–92. 27265510 10.1111/jhn.12386

[271] National Institute for Health and Care Excellence . Irritable bowel syndrome in adults: diagnosis and management. 2017. Available from https://www.nice.org.uk/guidance/cg61 [accessed 11 May 2022]. 32073807

[272] Halmos EP , Christophersen CT , Bird AR , Shepherd SJ , Muir JG , Gibson PR . Consistent prebiotic effect on gut microbiota with altered FODMAP intake in patients with Crohn's disease: a randomised, controlled cross‐over trial of well‐defined diets. Clin Transl Gastroenterol. 2016;7:e164. 27077959 10.1038/ctg.2016.22 PMC4855163

[273] Melgaard D , Sørensen J , Riis J , Ovesen TS , Leutscher P , Sørensen S , et al. Efficacy of FODMAP elimination and subsequent blinded placebo‐controlled provocations in a randomised controlled study in patients with ulcerative colitis in remission and symptoms of irritable bowel syndrome: a feasibility study. Nutrients. 2022;14(6):144– 164. 10.3390/nu14061296 PMC8955641 35334953

[274] Gionchetti P , Dignass A , Danese S , Magro Dias FJ , Rogler G , Lakatos PL , et al. European evidence‐based consensus on the diagnosis and management of Crohn's disease 2016: Part 2: surgical management and special situations. J Crohns Colitis. 2017;11(2):135–49. 27660342 10.1093/ecco-jcc/jjw169

[275] Harbord M , Annese V , Vavricka SR , Allez M. Barreiro‐de Acosta M , Boberg KM , et al. The first european evidence‐based consensus on extra‐intestinal manifestations in inflammatory bowel disease. J Crohns Colitis. 2016;10(3):239–54. 26614685 10.1093/ecco-jcc/jjv213 PMC4957476

[276] White A , Nunes C , Escudier M , Lomer MC , Barnard K , Shirlaw P , et al. Improvement in orofacial granulomatosis on a cinnamon‐ and benzoate‐free diet. Inflamm Bowel Dis. 2006;12(6):508–14. 16775496 10.1097/00054725-200606000-00011

[277] Torres J , Bonovas S , Doherty G , Kucharzik T , Gisbert JP , Raine T , et al. ECCO guidelines on therapeutics in Crohn's disease: medical treatment. J Crohns Colitis. 2020;14(1):4–22. 31711158 10.1093/ecco-jcc/jjz180

