# Supplementary material for: British Dietetic Association consensus guidelines on the nutritional assessment and dietary management of patients with inflammatory bowel disease
Source: J Hum Nutr Diet. 2022 Jul 21;36(1):336–77. doi: 10.1111/jhn.13054 (PMC10084145; doi:10.1111/jhn.13054)
Supplement: Supplementary file 1 — Supporting information. [file JHN-36-336-s001.docx]

**Supplementary material**

**Table S1**

| **Research questions** |
| --- |
| How do patients with IBD differ in anthropometric, micronutrient and dietary measurements compared to healthy controls and how should these nutritional assessment components be measured in clinical practice?  How can malnutrition, or risk of malnutrition, be assessed in people with IBD? |
| Can dietary interventions treat symptoms of malnutrition in people with IBD? |
| Are dietary interventions associated with improvements in markers of active IBD? |
| Are dietary interventions used as an adjuvant therapy associated with improvements in treatment response or treatment withdrawal |
| Are dietary interventions associated with maintenance of IBD remission? |
| Do dietary interventions affect surgical outcomes in patients with IBD? |
| Do dietary interventions affect symptoms of IBD strictures? |
| Are dietary interventions associated with reduced frequency of ileo–anal pouch inflammation? |
| Do dietary interventions affect stoma (e.g. jejunostomy, ileostomy or colostomy) function? |
| Do dietary interventions affect fistula healing and/or discomfort associated with fistulae in people with IBD? |
| Are dietary interventions associated with symptoms of short bowel syndrome? |
| When IBD is in remission, can functional gut symptoms, including diarrhoea, bloating, constipation and abdominal pain, be managed through dietary interventions? |
| Are dietary interventions associated with improvements in extra–intestinal manifestations (joint pain, mouth ulcers, skin lumps and blisters, eye inflammation) of IBD? |
| Are dietary interventions associated with improved orofacial granulomatosis (oral) symptoms? |
| Are dietary interventions associated with improved upper gastrointestinal Crohn’s disease? |
| Are dietary interventions associated with improved symptoms of rectal IBD? |
|  |

IBD inflammatory bowel disease

**Table S2 PICOT criteria**

| Table of inclusion and exclusion criteria following the PICOT approach | | | | |
| --- | --- | --- | --- | --- |
| **PICOT** |  | **Inclusion and exclusion criteria** |  | **Data extraction** |
| Participants |  | Adults ≥16 years with IBD^2^ diagnosed using standard procedures including endoscopy, histology. |  | Age, gender, IBD subtype, setting, location, concurrent medication or therapies, number of patients of each IBD–subtype included in intervention and comparator groups, inclusion and exclusion criteria. |
| Intervention |  | Any oral and/or enteral dietary intervention used in IBD administered for ≥1 week. Intervention must address the specific research question of interest for the systematic review |  | Dietary intervention, dose, frequency, duration of intervention |
| Comparators |  | Dietary intervention will be compared to placebo, standard care or no intervention, or if no comparator group end–point data will be compared to baseline. |  | Comparator population characteristics and dose, frequency, duration of comparator intervention. |
| Outcomes |  | Clinical, biochemical or quality of life data must be reported for a study to be included. At least one outcome must address the specific research question of interest for the systematic review |  | Outcomes measured, method of assessment (e.g. change in score), endpoint values. Acceptability and compliance measures will be recorded if available. |
|  | Nutrition assessment | Anthropometry; weight, BMI, muscle and/or fat mass, functional strength; handgrip dynamometry  Micronutrients  Dietary intake |  |  |
|  | Nutrition screening | Reported rates of nutrition/malnutrition risk in screening and self–screening tools |  |  |
|  | Oral nutritional support | Symptoms of malnutrition – anthropometry, biochemical markers (e.g. micronutrient status), functional markers (e.g. handgrip strength), dietary intake (e.g. energy, nutrient intake) |  |  |
|  | Induction of disease remission | Markers of active disease – disease activity score, biochemical markers (e.g. CRP, calprotectin, mucosal healing (e.g. MRI, histology, endoscopic score) |  |  |
|  |  | Adjuvant therapy: Improvements in treatment response or treatment withdrawal – disease activity score, biochemical markers (e.g. CRP, calprotectin, mucosal healing (e.g. MRI, histology, endoscopic score) or change in medication use |  |  |
|  | Remission maintenance | Maintenance of remission – disease activity score, biochemical markers (e.g. CRP, calprotectin) or mucosal healing (e.g. MRI, histology, endoscopic score), duration of time without increase in previously mentioned markers of active disease |  |  |
|  | Surgery | Surgical outcomes – post–surgical complication rate, formation of stoma, length of admission |  |  |
|  | Strictures | Symptoms of IBD strictures – abdominal pain, episodes of intestinal obstruction |  |  |
|  | Pouchitis | Ileo–anal pouch inflammation – markers of pouchitis (disease activity score, biochemical markers (e.g. CRP, calprotectin, mucosal healing (e.g. MRI, histology, endoscopic score) |  |  |
|  | Stoma | Stoma function e.g. output, gas, odour, hydration, body weight |  |  |
|  | Fistula | Fistula healing – fistula closure, mucosal healing, quality of life |  |  |
|  | Short bowel syndrome | Symptoms of short bowel syndrome – diarrhoea, bloating, weight loss, abdominal pain, malabsorption |  |  |
|  | Functional bowel symptoms | Functional bowel symptoms – e.g. diarrhoea, abdominal pain, abdominal bloating, constipation |  |  |
|  | Special situations | Extra–intestinal manifestations – presence of each extra–intestinal manifestation (e.g. aphthous ulcers)  Orofacial granulomatosis activity score and symptoms  Upper gastrointestinal Crohn’s disease activity  Symptoms of perianal IBD |  |  |
| Type of study | | Any study design will be eligible for inclusion.  No restriction on study size  PEN Global and the BDA guidance, more weighting was given to systematic reviews over randomised controlled trials (RCTs) over case–control studies and finally cross–sectional studies. |  | Type of study design, intention to treat and per protocol analysis, number of excluded patients, potential biases. |

**Table S3 Search strategy**

Embase search strategy.

["Crohn* disease", OR "Ulcerative colitis", OR IBD, OR "Inflammatory bowel disease", OR "Indeterminate colitis", OR Ileitis, OR *colitis, OR proctitis, OR Crohn]

AND

[Diet*, OR Nutrition, OR Probiotic, OR Lactobacill*, OR Bifidobacteri*, OR VSL pre/2 3, OR Prebiotic, OR "Inulin type fructan*", OR Galactooligosaccharide, OR Synbiotic, OR "Partially hydrolysed guar gum", OR Fructooligosaccharide, OR Fibre, OR Fiber, OR Oat, OR Psyllium, OR Plantago, OR Peppermint, OR CAM, OR "complimentary medicine", OR "curcumin" OR "alternative medicine", OR Cinnamon, OR Benzoate, OR "Enteral nutrition", OR "Formulated food", OR "Elemental diet", OR "Semi elemental diet", OR "Oral pre/2 supplement", OR Oxalate, OR LOFFLEX, OR "Low residue", OR FODMAP, OR "Fermentable carbohydrate", OR Fermented, OR Oligosaccharide, OR Lactose, OR Fructose, OR Dairy, OR Monosaccharide, OR SCD, OR "Specific carbohydrate diet", OR Carbohydrate, OR Paleolithic, OR Paleo, OR "Plant based", OR "Bone broth", OR IBD–AID, OR "Exclusion diet", "elimination diet", OR Vegetarian, OR Vegan, OR Macrobiotic, OR Mediterranean, OR Anti?inflammatory diet, OR "High fat* diet", OR "Gluten free", OR "Food intolerance", OR Fruit*, OR Vegetable*, OR "Omega–3", OR "Omega–6", OR "Fish oil", OR "Coconut oil", OR Micronutrient, OR Vitamin, OR Mineral, OR Protein, OR Sugar*, OR Microparticle, OR "Western diet", OR "Red meat"]

AND the relevant search terms for each research question

["nutritional assessment" OR "body composition" OR "body fat" OR "fat mass" OR "anthropometry" OR "lean mass" OR malnutrition OR "protein energy malnutrition" OR "muscle strength" OR "hand strength" OR "grip strength" OR "nutritional status" OR micronutrient OR "vitamin deficiency" OR "mineral deficiency" OR "dietary intake" OR diet OR "energy intake"]

[Malnutrition, OR undernutrition, OR "Nutrition* status", OR Sarcopenia]

[active OR symptomatic OR acute OR concomitant OR adjuvant OR adjunct OR maintenance OR maintain* OR relapse OR recurrence]

[preoperative OR resection OR presurgical OR anastomosis]

[strictur*]

[pouchitis OR pouch]

[colostomy OR ileostomy OR jejunostomy OR stoma]

[fistul*]

[“short bowel” OR “short bowel syndrome”]

[functional OR IBS OR “irritable bowel syndrome” OR quiescent]

[“extraintestinal manifestation*” OR “joint pain” OR “aphthous ulcer” OR “erythema nodosum” OR uveitis OR “ankylosing spondylitis” OR “pyoderma gangrenosum” OR “primary sclerosing cholangitis”]

[“orofacial granulomatosis”]

**Table S4 Excluded studies**

| **Topic and studies** | | **Reason for exclusion** |
| --- | --- | --- |
| **Nutrition assessment** | |  |
|  | Costello, et al. 2017(1) | Faecal transplant |
|  | Ghomraoui, et al. 2017(2) | Outcomes not relevant |
|  | Ghomraoui, et al. 2017(3) | Outcomes not relevant |
|  | Głąbska, et al. 2016(4) | No control group |
|  | Mentella, et al. 2018(5) | Limited detail |
|  | Principi, et al. 2016(6) | Assessing risk |
|  | Rashvand, et al. 2015a(7) | Assessing risk |
|  | Rashvand, et al. 2015b(8) | Assessing risk |
|  | Shivappa, et al. 2016(9) | Risk for UC |
|  | Sturniolo, et al. 1998(10) | IBS controls |
|  | Tromm, et al. 1994(11) | No control group |
|  | Uchiyama, et al. 2013(12) | Not a relevant outcome |
|  | Wang, et al. 2013(13) | Risk for UC |
|  | Zhang, et al. 2012(14) | Outcomes not relevant leptin & ghrelin |
| **Nutrition screening** | |  |
|  | Dunning, et al 2019(15) | Does not compare with nutrition markers |
|  | Einav, et al. 2021(16) | Does not compare with nutrition markers |
|  | Fonalleras-Marcos, et al. 2021(17) | Does not compare with nutrition markers |
|  | Korwel, et al. 2022(18) | No screening tool used |
|  | Lomer et al 2019(19) | Nutrition screening not compared with any other tool |
|  | Lui, et al. 2022(20) | Limited detail |
|  | Sumi et al 2016(21) | No data to compare with other nutrition screening tool |
| **Oral nutritional support** | |  |
|  | Abad–Lacruz et al. 1988(22) | Not oral nutritional support |
|  | Capristo et al. 2000(23) | Not oral nutritional support |
|  | Royall et al. 1995(24) | Not oral nutritional support |
|  | Santarpia et al. 2009(25) | Insufficient data |
| **Induction of remission** | |  |
|  | **Enteral nutrition** |  |
|  | Kamata et al. 2015(26) | Insufficient data |
|  | Ohara et al. 2017(27) | Insufficient data |
|  | Sakurai et al. 2015(28) | Data duplication |
|  | Schulman et al. 2015(29) | Paediatric study |
|  | Shinozaki et al. 2017(30) | Insufficient data |
|  | Tang et al. 2018(31) | Not nutritional intervention study |
|  | **Elimination diets** |  |
|  | Anderson et al. 2015(32) | Not a nutritional intervention |
|  | Bamba et al. 2018(33) | Low level evidence |
|  | Bataga et al. 2015(34) | Insufficient data |
|  | Burgis et al. 2016(35) | Paediatric study |
|  | Candy et al. 1995(36) | Not a nutritional intervention |
|  | Ou et al. 2021(37) | Did not measure disease activity |
|  | Sigall-Boneh et al. 2014 (38) | Paediatric study |
|  | **Probiotics** |  |
|  | Rajendran et al. 2011(39) | Low level evidence |
|  | Sood et al. 2020(40) | Cohort study |
| **Remission maintenance** | |  |
|  | **Partial enteral nutrition** | |
|  | Hirai et al. 2013(41) | Case series observational. Did not reach consensus |
|  | Hirai et al. 2018(42) | Case series observational. Did not reach consensus |
|  | Sazuka et al. 2012(43) | Case series observational. Did not reach consensus |
|  | Sugita et al. 2018(44) | Case control. Did not reach consensus |
|  | Yamamoto et al. 2010(45) | Case series observational. Did not reach consensus |
|  | Yamamoto et al. 2015(46) | Case series observational. Did not reach consensus |
|  | Yoshimura et al. 2014(47) | Case control. Did not reach consensus |
|  | **Fibre** |  |
|  | Wilson et al 2021(48) | Not RCT |
|  | Yao et al. 2017(49) | Short duration |
|  | **Elimination diets** |  |
|  | Barnes et al. 2017(50) | Not nutrition intervention |
|  | Levenstein et al. 1985(51) | Outcomes not defined |
|  | **Probiotics** |  |

|  | Borruel et al.2018(52) | Not probiotic |
| --- | --- | --- |

|  | Graziani et al. 2017(53) | Insufficient data |
| --- | --- | --- |

|  | Hassan et al.2016(54) | Not probiotic | |
| --- | --- | --- | --- |
|  | Irving et al.2018(55) | Not probiotic | |
|  | Karakan et al.2013(56) | Low level evidence | |
|  | Kaur et al.2009(57) | Probiotic arm not isolated | |
|  | Lin et al.2017(58) | Low level evidence | |
|  | Mangiola et al. 2016(59) | Insufficient data | |
|  | Plehutsa et al.2015(60) | Low level evidence | |
|  | Rohani et al.2015(61) | Not IBD | |
|  | Sisson et al.2015(62) | Low level evidence | |
|  | Soloveyeva et al. 2014(63) | Insufficient data | |
|  | Suzuki et al. 2013(64) | Duplication of data | |
|  | Sydorchuk et al. 2016(65) | Low level evidence | |
|  | Valcheva et al. 2012(66) | Not probiotic | |
| **Surgery** | | |  |
|  | Ceccarelli et al. 2017(67) | Insufficient data | |
|  | Charvin et al. 2013(68) | Insufficient data | |
|  | Feng et al. 2014(69) | Insufficient data | |
|  | Gong et al. 2016(70) | Not nutrition intervention | |
|  | Guo et al. 2017(71) | Insufficient data | |
|  | Nguyen et al. 2016(72) | Not nutrition intervention | |
|  | Patel et al. 2016(73) | Insufficient data | |
|  | Smedh et al. 2002(74) | Not nutrition intervention | |
|  | Abdalla et al. 2021(75) | Cohort | |
|  | Fiorindi et al. 2021(76) | Prospective observational | |
|  | Gordon–Dixon et al. 2021(77) | Cohort | |
|  | Wall et al. 2022(78) | RCT - no outcomes of interest | |
|  | Weber et al. 2021(79) | Cohort | |
|  | Voitk et al. 1973(80) | Insufficient data | |
|  | Zerbib et al. 2010(81) | Insufficient data | |
|  | Peters et al. 2018(82) | Duration less than 7 days | |
|  | Marafini et al 2020(83) | EEN for 24 hours only sporadically | |
|  | Costa Santos et al 2020(84) | Cohort | |
|  | Ge et al 2019(85) | Cohort | |
|  | Saito et al 2020(86) | Insufficient data | |

| **Strictures** | | | |
| --- | --- | --- | --- |
|  | Bergeron et al. 2018(87) | Not a dietary intervention | |
|  | Silk et al. 1989(88) | Not a dietary intervention | |
|  | Silk et al. 1992(89) | Not a dietary intervention | |
|  | Spencer et al. 1994(90) | Not a dietary intervention | |
|  | Woolner et al. 1998(91) | No comparator outcomes reported | |
|  | Yamamoto et al. 2006(92) | Not a defined dietary intervention | |
| **Pouchitis** | | |  |
|  | Bengtsson et al 2016(93) | Did not reach consensus | |
|  | Kuisma et al 2003(94) | Did not reach consensus | |
|  | Alles et al. 1997(95) | Study does not address research question | |
|  | Friedman et al. 2010(96) | No comparator outcomes reported | |
|  | Godny et al. 2016(97) | Not a dietary intervention | |
|  | Ianco et al. 2013(98) | Not a dietary intervention | |
|  | McKinley et al. 2009(99) | No comparator outcomes reported | |
|  | Sagar et al. 1995(100) | No comparator outcomes reported | |

| **Stoma** | |  |
| --- | --- | --- |
|  | Ellegard et al. 1997(101) | Short duration |
|  | Ellegard et al. 2000(102) | Short duration |
|  | Ellegard et al. 2007(103) | Short duration |
|  | Hylander et al. 1990(104) | Short duration |
|  | Jeppeson et al. 1998(105) | Short duration |
|  | Kennedy et al. 1982(106) | Short duration |
|  | M’Koma et al. 1994(107) | Short duration |
|  | Miettinen et al. 1971(108) | Short duration |
|  | Culkin et al. 2014(109) | Short duration |
|  | Barrett et al. 2010(110) | Short duration |
| **Fistula** | |  |
|  | Bury et al. 1974(111) | Case report |
|  | Calam et al. 1980(112) | Case report |
|  | Dardai et al. 1991 (113) | Not IBD |
|  | Hill et al. 1975(114) | Intervention duration less than 1 week |
|  | Ortiz et al. 2017(115) | IBD excluded |
|  | Wu et al. 2016(116) | Not a dietary intervention study |
| **Short bowel syndrome** | |  |
|  | Araujo et al. 2008(117) | Outcomes not relevant |
|  | Atia et al. 2011(118) | Intervention not relevant |
|  | Byrne et al. 1996(119) | Intervention not relevant |
|  | Canada et al. 2012(120) | Intervention not relevant |
|  | Hylander et al. 1978(121) | Outcomes not relevant |
|  | Jeppesen et al. 2012(122) | Intervention not relevant |
|  | Kunkel et al. 2011(123) | Intervention not relevant |
|  | Lange et al. 2003(124) | Intervention not relevant |
|  | Messing et al. 1991(125) | Outcomes not relevant |
|  | Ovesen et al. 1983(126) | Outcomes not relevant |
|  | Pagoldh et al. 2008(127) | Outcomes not relevant |
|  | Scolapio et al. 2001(128) | Intervention not relevant |
|  | Scolapio et al. 2001 (129) | Intervention not relevant |
|  | Small et al. 2015(130) | Outcomes not relevant |
|  | Stoidis et al. 2011(131) | Outcomes not relevant |
|  | Weiming et al. 2004(132) | Intervention not relevant |
|  | Wu et al. 2003(133) | Intervention not relevant |
| **Functional symptoms** | |  |
|  | **Elimination diets** |  |
|  | Cox et al. 2017(134) | Short duration |
|  | Curro et al. 2017(135) | Not a nutrition intervention |
|  | Gearry et al. 2009(136) | Low level evidence |
|  | Hallert et al. 2003(137) | Did not reach consensus |
|  | Ibanez et al. 2016(138) | Insufficient data |
|  | Ibanez et al. 2018(139) | Low level evidence |
|  | Komperod et al. 2017(140) | Low level evidence |
|  | Maagaard et al. 2016(141) | Low level evidence |
|  | Nawawi et al. 2017(142) | Not IBD |
|  | Pederson et al. 2017(143) | Low level evidence |
|  | Prince et al. 2016(144) | Low level evidence |
|  | Sheasgreen et al. 2017(145) | Not a nutrition intervention |
|  | Swanson et al. 2010(146) | Not a nutrition intervention |
|  | Tapete et al. 2018(147) | Low level evidence |
|  | Testa et al. 2018(148) | Low level evidence |
|  | **Complementary and alternative medicine** | |
|  | Albrecht et al. 2014(149) | Insufficient data |
|  | **Nutrients** |  |
|  | Marotta et al. 2003(150) | Not a nutritional intervention |
|  | McNelly et al. 2016(151) | Insufficient data |
|  | Raftery et al. 2013(152) | Insufficient data |
|  | Scholten et al. 2018(153) | Insufficient data |
|  | **Whole diets** |  |
|  | Azpiroz et al. 2014(154) | Not IBD |
|  | Chiba et al. 2017(155) | Insufficient data |
|  | Sabino et al. 2017(156) | Not IBD |
|  | **Fibre** |  |
|  | Cockerell et al. 2012(157) | Not IBD |
|  | Gilmore et al. 2016(158) | Not fibre |
|  | Hallert et al. 2003(137) | Insufficient data |
|  | Mutlu et al. 2016(159) | Insufficient data |
|  | Riley et al. 1991(160) | Insufficient data |
|  | Yao et al. 2017(49) | Short duration |
|  | **Probiotics** |  |
|  | Abbas et al. 2014(161) | Not IBD |
|  | Fujimori et al. 2009(162) | Insufficient data |
|  | Krammer et al. 2006(163) | Insufficient data |
|  | Kruis et al. 2012(164) | Not IBD |
|  | Marotta et al. 2003(150) | Not functional outcomes |
|  | Sisson et al. 2015(62) | Not functional outcomes |
| **OFG** | |  |
|  | Campbell et al 2013(165) | Did not reach consensus |
|  | Campbell et al 2013(166) | Did not reach consensus |
|  | Fitzpatrick et al. 2010(167) | Not nutritional intervention |
|  | Goel et al 2015(168) | Did not reach consensus |
|  | McCartan et al. 2010(169) | Not nutritional intervention |
|  | Patel et al. 2013(170) | Not nutritional intervention |

**Table S5 Searches**

|  | Nutritional assessment | Nutritional screening | Oral nutritional support | Induction of remission and maintenance of remission | Surgery | Strictures | Pouchitis | Stoma | Fistula | Short bowel syndrome | Functional bowel symptoms | Extraintestinal manifestations, upper GI CD, perianal | Orofacial granuloma |
| --- | --- | --- | --- | --- | --- | --- | --- | --- | --- | --- | --- | --- | --- |
| Studies identified (not duplicate) | 2555 | 1166 | 7681 | 6513 | 3230 | 1665 | 1499 | 1895 | 2645 | 1273 | 2283 | 3028 | 94 |
| Studies not appropriate from title/abstract | 2480 | 1150 | 7673 | 6409 | 3200 | 1655 | 1485 | 1885 | 2637 | 1255 | 2240 | 3028 | 87 |
| Studies retrieved for evaluation | 75 | 16 | 8 | 104 | 30 | 10 | 14 | 10 | 8 | 18 | 43 | 0 | 7 |
| Studies excluded (did not meet inclusion criteria) | 14 | 7 | 4 | 41 | 17 | 6 | 9 | 10 | 6 | 18 | 39 | 0 | 6 |
| Included studies | 61 | 9 | 4 | 63 | 13 | 4 | 5 | 0 | 2 | 0 | 4 | 0 | 1 |

**Table S6 Expert IBD Panel Affiliations**

| **First Name** | **Last Name** | **Profession/Representation** | **Affiliation** |  |
| --- | --- | --- | --- | --- |
| Paul | Blaker | Gastroenterologist | Maidstone and Tunbridge Wells NHS Trust | |
| M | CW | Patient | N/A | |
| Omar | Faiz | Colorectal Surgeon | Sheffield Teaching Hospital NHS Trust |  |
| Aileen | Fraser | IBD Nurse | University Hospitals Bristol NHS Foundation Trust | |
| Kostas | Gerasimidis | Dietitian | Guy's and St Thomas' NHS Foundation Trust, London |  |
| Chris | Lamb | Gastroenterologist | Newcastle University | |
| Tim | Raine | Gastroenterologist | Addenbrookes Hospital, Cambridge |  |
| G | S | Patient | N/A |  |
| Helen | Terry | Patient representation | Crohn’s and Colitis UK | |

**Table S7 Diet and IBD eDelphi Consensus Group Affiliations**

| **First Name** | **Last Name** | **Profession/Representation** | **First Affiliation** | **Second Affiliation** |
| --- | --- | --- | --- | --- |
| Katie | Adams | Colorectal Surgeon | Guy's and St Thomas' NHS Foundation Trust, London |  |
| Marialice | Albertini | Dietitian | St George's Healthcare, London |  |
| Aaron | Bancil | Gastroenterologist | King's College London, London |  |
| Kevin | Barrett | General Practitioner | New Road Surgery, Hertfordshire | |
| Matt | Brookes | Gastroenterologist | The Royal Wolverhampton NHS Trust | |
| Steven | Brown | Colorectal Surgeon | Sheffield Teaching Hospital NHS Trust |  |
| Ann | Chui | Dietitian | Princess Alexandra Hospital NHS Trust, Harlow |  |
| Sean | Coleman | Dietitian | Brighton & Sussex University Hospitals |  |
| Benjamin | Crooks | Gastroenterologist | University of Manchester |  |
| Ben | Disney | Gastroenterologist | University Hospital Coventry & Warwickshire | |
| Megan | Drake | Dietitian | King's College Hospital |  |
| Aileen | Fraser | IBD Nurse | University Hospitals Bristol NHS Foundation Trust |  |
| Kostas | Gerasimidis | Dietitian | University of Glasgow |  |
| Lisa | Gervais | Paediatric IBD Nurse | Royal Hospital for Children, Glasgow |  |
| Lynn | Gray | IBD Nurse | Wirral University Teaching Hospital NHS Trust | |
| Barney | Hawthorne | Gastroenterologist | University Hospital of Wales, Cardiff |  |
| Sami | Hoque | Gastroenterologist | Whipps Cross University Hospital |  |
| A | J | Patient | Crohn's and Colitis UK |  |
| Yvonne | Jeanes | Dietitian | Roehampton University |  |
| Katie | Keetarut | Dietitian | University College Hospital NHS Foundation Trust | |
| Alice | Kershaw | Dietitian | Guy's and St Thomas' NHS Foundation Trust, London |  |
| Angela | Kidd | Dietitian | NHS Lothian |  |
| Chris | Lamb | Gastroenterologist | Newcastle University |  |
| James | Lindsay | Gastroenterologist | Barts Health Trust, London |  |
| Miranda | Lomer | Dietitian | Guy's and St Thomas' NHS Foundation Trust, London | King's College London |
| Sophie | Martin | Dietitian | Bradford Teaching Hospitals NHS Foundation Trust | |
| Joel | Mawdsley | Gastroenterologist | Guy's and St Thomas' NHS Foundation Trust, London |  |
| Adam | McCulloch | Gastroenterologist | University Hospital Birmingham |  |
| Helen | MCGURRIN | Dietitian | Kent and Canterbury Hospital |  |
| Kerryn | Moolenschot | Dietitian | St George's Healthcare NHS Trust |  |
| Allison | Nightingale | IBD Nurse | Addenbrookes Hospital, Cambridge |  |
| Aisling | O'Connor | Dietitian | Manchester University Foundation Trust | |
| Dearbháile | O'Hanlon | Dietitian | Guy's and St Thomas' NHS Foundation Trust, London |  |
| Francesca | Onori | Dietitian | Central Middlesex Hospital |  |
| Philip | Oppong | Gastroenterologist | University Hospitals of Derby and Burton NHS Foundation Trust | |
| Nina | Powell | Dietitian | Addenbrookes Hospital, Cambridge |  |
| Nick | Probyn | Dietitian | Buckinghamshire Healthcare NHS Trust | |
| Nabil | Quraishi | Gastroenterologist | University Hospitals Birmingham |  |
| Alicia | Sandal | Dietitian | Guy's and St Thomas' NHS Foundation Trust, London |  |
| Leah | Seamark | Dietitian | Somerset Foundation Trust, Bridgwater |  |
| Rozanna | Slade | Dietitian | West Middlesex University Hospital | |
| A | S | Patient | Crohn's and Colitis UK |  |
| Jim | Stewart | Gastroenterologist | Leicester Royal Infirmary |  |
| Anni | Summers | Dietitian | Mid Essex Hospital Services NHS Trust | |
| Graeme | Syme | Dietitian | Barts Health Trust |  |
| Carole | Tan | Dietitian | Bromley Hospitals NHS Trust |  |
| Lisa | Vokes | Dietitian | John Radcliffe Hospital, Oxford |  |
| Alice | Walker | Dietitian | Brighton & Sussex University Hospitals |  |
| Catherine | Wall | Dietitian | University of Otago, New Zealand | King's College London |
| P | W | Patient | Crohn's and Colitis UK |  |
| Kevin | Whelan | Dietitian | King's College London |  |
| Andy | Williams | Colorectal Surgeon | Guy's and St Thomas' NHS Foundation Trust, London |  |
| Bridgette | Wilson | Dietitian | Guy's and St Thomas' NHS Foundation Trust, London | King's College London |
| Lisa | Younge | IBD Nurse | Crohn's and Colitis UK |  |
| Kristina | Zaremba | Dietitian | Epsom and St Helier NHS Trust, Surrey |  |

**Supplementary references**

1. Costello S, Mashei C, Bryant R, Katsikeros R, Waters O, Makanyanga J, et al. Comparison of dietary nutrient and food additive intake between patients with moderately active ulcerative colitis, healthy stool donors and the general Australian population. Journal of Crohn's and Colitis. 2017;11 (Supplement 1):S277.

2. Ghomraoui FA, Alotaibi ST, Alharthi MA, Asiri SS, Almadi MA, Alharbi OR, et al. Plasma ghrelin and leptin in patients with inflammatory bowel disease and its association with nutritional status. Saudi J Gastroenterol. 2017;23(3):199-205.

3. Ghomraoui F, Alotaibi S, Alharthi M, Asiri S, Almadi M, Alharbi O, et al. Serum Ghrelin and Leptin Levels of Patients With Inflammatory Bowel Disease Compared to a Control Group and its Association With Nutritional Status: A Case-Control Study. Clin Gastroenterol Hepatol. 2017;15(1):e43-e4.

4. Głąbska D, Guzek D, Zakrzewska P, Włodarek D, Lech G. Lycopene, lutein and zeaxanthin may reduce faecal blood, mucus and pus but not abdominal pain in individuals with ulcerative colitis. Nutrients. 2016;8(10).

5. Mentella MC, Scaldaferri F, Striano E, Castorina M, Musca T, Rinninella E, et al. Prevalence of malnutrition in IBD patients. Digestive and Liver Disease. 2018;50 (2 Supplement 1):e234-e5.

6. Principi M, Losurdo G, Deflorio V, Ranaldo N, Iannone A, Barone M, et al. UEG Week 2016 Poster Presentations. United Eur Gastroenterol J. 2016;4(5_suppl):A157-A720.

7. Rashvand S, Somi MH, Rashidkhani B, Hekmatdoost A. Dietary fatty acid intakes are related to the risk of ulcerative colitis: a case-control study. Int J Colorectal Dis. 2015;30(9):1255-60.

8. Rashvand S, Somi MH, Rashidkhani B, Hekmatdoost A. Dietary protein intakes and risk of ulcerative colitis. Med J Islam Repub Iran. 2015;29(1):253.

9. Shivappa N, Hebert JR, Rashvand S, Rashidkhani B, Hekmatdoost A. Inflammatory Potential of Diet and Risk of Ulcerative Colitis in a Case-Control Study from Iran. Nutr Cancer. 2016;68(3):404-9.

10. Sturniolo GC, Mestriner C, Lecis PE, D'Odorico A, Venturi C, Irato P, et al. Altered plasma and mucosal concentrations of trace elements and antioxidants in active ulcerative colitis. Scand J Gastroenterol. 1998;33(6):644-9.

11. Tromm A, Rickels K, Huppe D, Wiebe V, May B. [Osteopenia in chronic inflammatory bowel diseases. Results of a cross-sectional study using quantitative computerized tomography]. Leber Magen Darm. 1994;24(1):23-6, 9-30.

12. Uchiyama K, Odahara S, Nakamura M, Koido S, Katahira K, Shiraishi H, et al. The fatty acid profile of the erythrocyte membrane in initial-onset inflammatory bowel disease patients. Dig Dis Sci. 2013;58(5):1235-43.

13. Wang YF, Ou-Yang Q, Xia B, Liu LN, Gu F, Zhou KF, et al. Multicenter case-control study of the risk factors for ulcerative colitis in China. World J Gastroenterol. 2013;19(11):1827-33.

14. Zhang S, Mei Q, Xu JM, Zhang X, Yang QF, Zhang LP, et al. Serum leptin and ghrelin levels in patients with active ulcerative colitis. Chinese J Clin Nutr. 2012;20(1):10-4.

15. Dunning A, Sechi A, Williams A, Connor S, Ng W, Kuzet G, et al. Use of a novel tool to identify nutritional risk in an inflammatory bowel disease outpatient service: Liverpool IBD-nutrition screen. J Gastroenterol Hepatol. 2019;34(Supplement 2):200.

16. Einav L, Hirsch A, Ron Y, Cohen NA, Lahav S, Kornblum J, et al. Risk Factors for Malnutrition among IBD Patients. Nutrients. 2021;13(11).

17. Fonalleras-Marcos D, Fragkos K, Vega R, McCartney S, Parisi I, Seward E, et al. Nutritional status and disease activity in Crohn's disease: Preliminary data. Gut. 2021;70(SUPPL 1):A185.

18. Korwel KM, Hjortswang H, Eberhardson M, Pihl Lesnovska K. Prevalence of malnutrition, risk of malnutrition and quality of life among patients with Inflammatory Bowel Disease. J Crohns Colitis. 2022;16(Supplement 1):i147-i8.

19. Lomer MCE, Cahill O, Baschali A, Partha Sarathy P, Sarantidou M, Mantzaris GJ, et al. A multicentre Study of Nutrition Risk Assessment in Adult Patients with Inflammatory Bowel Disease Attending Outpatient Clinics. Ann Nutr Metab. 2019;74(1):18-23.

20. Liu J, Ge X, Ouyang C, Wang D, Zhang X, Liang J, et al. Prevalence of Malnutrition, Its Risk Factors, and the Use of Nutrition Support in Patients with Inflammatory Bowel Disease. Inflamm bowel dis. 2022;04.

21. Sumi R, Nakajima K, Iijima H, Wasa M, Shinzaki S, Nezu R, et al. Influence of nutritional status on the therapeutic effect of infliximab in patients with Crohn's disease. Surgery Today. 2016;46(8):922-9.

22. Abad-Lacruz A, Fernandez-Banares F, Cabre E, Gil A, Esteve M, Gonzalez-Huix F, et al. The effect of total enteral tube feeding on the vitamin status of malnourished patients with inflammatory bowel disease. Int J Vitam Nutr Res. 1988;58(4):428-35.

23. Su Q, Zhang Y. Glaucocalyxin A attenuates angiotensin II-induced cardiac fibrosis in cardiac fibroblasts. Biochem Biophys Res Commun. 2018;503(3):1949-54.

24. Miyata Y, Shimazaki M, Komatsu Y, Nakamura Y, Oka A. [Nonconvulsive status epilepticus as an initial symptom in a boy with frontal lobe epilepsy]. No To Hattatsu. 2014;46(4):301-6.

25. Santarpia L, Contaldo F. Gut flora in intestinal and extraintestinal diseases: A general overview and the effects of artificial nutrition. Curr Nutr Food Sci. 2009;5(3):180-92.

26. Kamata N, Oshitani N, Watanabe K, Watanabe K, Hosomi S, Noguchi A, et al. Efficacy of concomitant elemental diet therapy in scheduled infliximab therapy in patients with Crohn's disease to prevent loss of response. Dig Dis Sci. 2015;60(5):1382-8.

27. Ohara N, Mizushima T, Iijima H, Takahashi H, Hiyama S, Haraguchi N, et al. Adherence to an elemental diet for preventing postoperative recurrence of Crohn's disease. Surg Today. 2017;47(12):1519-25.

28. Sakurai T, Yoshihama S, Katsuno T, Saito K, Minemura S, Maruoka D, et al. Concomitant use of enteral nutrition therapy increases sustained response to infliximab in patients with crohn's disease. Gastroenterology. 2015;1):S249.

29. Schulman J, Shaoul R. Maintenance of remission with partial enteral nutrition therapy in pediatric Crohn's disease: A retrospective study. J Crohns Colitis. 2015;1):S298.

30. Shinozaki M. The therapies influencing postoperative surgical recurrence in Crohn's disease. J Crohns Colitis. 2017;11 (Supplement 1):S254.

31. Tang W. Enteral nutrition for the maintenance of remission in adults with inactive Crohn's disease: A meta-analysis. J Crohns Colitis. 2018;12 (Supplement 1):S378.

32. Anderson JL, Hedin CR, Benjamin JL, Koutsoumpas A, Ng SC, Hart AL, et al. Dietary intake of inulin-type fructans in active and inactive Crohn's disease and healthy controls: a case-control study. J Crohns Colitis. 2015;9(11):1024-31.

33. Bamba S, Takahashi K, Imaeda H, Nishida A, Kawahara M, Inatomi O, et al. Effect of fermented vegetable beverage containing Pediococcus pentosaceus in patients with mild to moderate ulcerative colitis. Biomed Rep. 2018;9(1):74-80.

34. Bataga SM, Torok I, Macarie M, Negovan A, Botianu A. Rifaximine and probiotics in the treatment of mild relaps of left side ulcerative colitis. Eur J Clin Invest. 2015;2):39-40.

35. Burgis JC, Nguyen K, Park KT, Cox K. Response to strict and liberalized specific carbohydrate diet in pediatric Crohn's disease. World J Gastroenterol. 2016;22(6):2111-7.

36. Yurk Quadlin N. When children affect parents: Children's academic performance and parental investment. Soc Sci Res. 2015;52(11):671-85.

37. Ou Q, Wang L, Wang K, Shao P. Effect of probiotics supplementation combined with WeChat platform health management on nutritional status, inflammatory factors, and quality of life in patients with mild-to-moderate ulcerative colitis: a randomized trial. Ann Palliat Med. 2021;10(6):6606-16.

38. Sigall-Boneh R, Pfeffer-Gik T, Segal I, Zangen T, Boaz M, Levine A. Partial enteral nutrition with a Crohn's disease exclusion diet is effective for induction of remission in children and young adults with Crohn's disease. Inflamm Bowel Dis. 2014;20(8):1353-60.

39. Rajendran N, Kumar D. Food-specific IgG4-guided exclusion diets improve symptoms in Crohn's disease: a pilot study. Colorectal Dis. 2011;13(9):1009-13.

40. Sood A, Singh A, Sudhakar R, Midha V, Mahajan R, Mehta V, et al. Exclusive enteral nutrition for induction of remission in anti-tumor necrosis factor refractory adult Crohn's disease: the Indian experience. Intest Res. 2020;18(2):184-91.

41. Hirai F, Ishihara H, Yada S, Esaki M, Ohwan T, Nozaki R, et al. Effectiveness of concomitant enteral nutrition therapy and infliximab for maintenance treatment of Crohn's disease in adults. Dig Dis Sci. 2013;58(5):1329-34.

42. Hirai F, Ishida T, Takeshima F, Yamamoto S, Yoshikawa I, Ashizuka S, et al. Effect of a concomitant elemental diet with maintenance anti-tumor necrosis factor-alpha antibody therapy in patients with Crohn's disease: A multicenter, prospective cohort study. J Gastroenterol Hepatol. 2019;34(1):132-9.

43. Sazuka S, Katsuno T, Nakagawa T, Saito M, Saito K, Matsumura T, et al. Concomitant use of enteral nutrition therapy is associated with sustained response to infliximab in patients with Crohn's disease. Eur J Clin Nutr. 2012;66(11):1219-23.

44. Sugita N, Watanabe K, Kamata N, Yukawa T, Otani K, Hosomi S, et al. Efficacy of a concomitant elemental diet to reduce the loss of response to adalimumab in patients with intractable Crohn's disease. J Gastroenterol Hepatol. 2018;33(3):631-7.

45. Yamamoto T. Prevention of recurrence after surgery for Crohn's disease: efficacy of infliximab. World J Gastroenterol. 2010;16(43):5405-10.

46. Yamamoto T. [Current trends in the management of Crohn's disease]. Nihon Geka Gakkai Zasshi. 2015;116(2):82-6.

47. Yoshimura N, Kawaguchi T, Sako M, Saniabadi A, Takazoe M. In patients with crohn's disease, concomitant enteral nutrition reduces the loss of response to adalimumab maintenance therapy. Gastroenterology. 2014;1):S-382.

48. Wilson B, Eyice O, Koumoutsos I, Lomer MC, Irving PM, Lindsay JO, et al. Prebiotic Galactooligosaccharide Supplementation in Adults with Ulcerative Colitis: Exploring the Impact on Peripheral Blood Gene Expression, Gut Microbiota, and Clinical Symptoms. Nutrients. 2021;13(10).

49. Yao CK, Burgell RE, Taylor KM, Ward MG, Barrett JS, Muir JG, et al. Effects of acute changes in fermentable fiber intake on regional colonic fermentation and transit in patients with quiescent ulcerative colitis. J Gastroenterol Hepatol. 2017;32 (Supplement 3):140-1.

50. Barnes EL, Nestor M, Onyewadume L, de Silva PS, Korzenik JR, Investigators D. High Dietary Intake of Specific Fatty Acids Increases Risk of Flares in Patients With Ulcerative Colitis in Remission During Treatment With Aminosalicylates. Clin Gastroenterol Hepatol. 2017;15(9):1390-6 e1.

51. Levenstein S, Prantera C, Luzi C, D'Ubaldi A. Low residue or normal diet in Crohn's disease: a prospective controlled study in Italian patients. Gut. 1985;26(10):989-93.

52. Borruel N, Herrera-DeGuise C, Varela E, Barreiro M, Beltran B, Gisbert JP, et al. Combination of prebiotic FOS and adalimumab for prevention of dysbiosis in active Crohn's disease: A pilot study. J Crohns Colitis. 2018;12 (Supplement 1):S551-S2.

53. Graziani C, Petito V, Del Chierico F, Mangiola F, Pecere S, Schiavoni E, et al. Escherichia coli nissle 1917 modulate gut microbiota composition in ulcerative colitis patients. Dig Liver Dis. 2017;49 (Supplement 2):e118-e9.

54. Hassan SI, Hassan SMA, Shafia, Nausheen. Role of probiotics in treatment of inflammatory bowel disease. Indian J Gastroenterol. 2016;35 (1 Supplement):A38.

55. Irving PM, Iqbal T, Nwokolo C, Subramanian S, Bloom S, Prasad N, et al. A Randomized, Double-blind, Placebo-controlled, Parallel-group, Pilot Study of Cannabidiol-rich Botanical Extract in the Symptomatic Treatment of Ulcerative Colitis. Inflamm Bowel Dis. 2018;24(4):714-24.

56. Karakan T, Tahtaci M, Unal S. The effect of adjuvant synbiotic combination on induction of remission in patients with mildmoderate ulcerative colitis. United Eur Gastroenterol J. 2013;1):A436.

57. Kaur IP, Kuhad A, Garg A, Chopra K. Probiotics: delineation of prophylactic and therapeutic benefits. J Med Food. 2009;12(2):219-35.

58. Lin H, Lim WC. Histological remission with probiotics alone in ulcerative colitis. J Gastroenterol Hepatol. 2017;32 (Supplement 3):131.

59. Mangiola F, Del Chierico F, Graziani C, Petito V, Pecere S, Schiavoni E, et al. Escherichia coli nissle 1917 modulate gut microbiota composition in ulcerative colitis patients. Helicobacter. 2016;21 (Supplement 1):174.

60. Plehutsa O, Sydorchuk R, Sydorchuk L, Sydorchuk I. Changes of colonic microbiota and efficacy of oral probiotic therapy in IBD. United Eur Gastroenterol J. 2015;1):A437.

61. Rohani M, Noohi N, Talebi M, Katouli M, Pourshafie MR. Highly Heterogeneous Probiotic Lactobacillus Species in Healthy Iranians with Low Functional Activities. PLoS One. 2015;10(12):e0144467.

62. Sisson G, Hayee B, Bjarnason I. Assessment of a multi strain probiotic (symprove) in IBD. Gastroenterology. 2015;1):S531.

63. Solovyeva O. Probiotics can extend remission of ulcerative colitis. J Crohns Colitis. 2014;1):S221.

64. Suzuki Y, Yamada A, Yoshimatsu Y, Takeuchi K. A randomized double blind placebo controlled trial to assess the efficacy of a probiotic preparation as maintenance therapy in patients with ulcerative colitis. J Crohns Colitis. 2013;7 (SUPPL.1):S246.

65. Sydorchuk A, Sydorchuk R, Sydorchuk L, Sydorchuk I, Plehutsa O. Probiotics are less effective than mesalazine in maintaining remissions in IBD. United Eur Gastroenterol J. 2016;4 (5 Supplement 1):A453.

66. Valcheva R, Koleva P, Meijer BJ, Walter J, Ganzle M, Dieleman LA. Beta-fructans reduce inflammation in mild to moderate ulcerative colitis through specific microbiota changes associated with improved butyrate formation and MUC2 expression. Gastroenterology. 2012;1):S196.

67. Ceccarelli L, Franceschi M, Bertani L, Nieri C, De Bortoli N, Mumolo G, et al. Can enteral polymeric diet change the post-surgical outcome in Crohn's disease patients? A pilot study. J Crohns Colitis. 2017;11 (Supplement 1):S353-S4.

68. Charvin M, Flamant M, Meurette G, Trang C, Duchalais E, Lehur PA, et al. Post-operative complications are not decreased by a prolonged nutritional support in patients with IBD operated for a perforating disease. United Eur Gastroenterol J. 2013;1):A226-A7.

69. Feng Y, Li Y, Mei S, Zhang L, Gong J, Gu L, et al. Exclusive enteral nutrition ameliorates mesenteric adipose tissue alterations in patients with active Crohn's disease. Clin Nutr. 2014;33(5):850-8.

70. Gong J, Wei Y, Gu L, Li Y, Guo Z, Sun J, et al. Outcome of Surgery for Coloduodenal Fistula in Crohn's Disease. J Gastrointest Surg. 2016;20(5):976-84.

71. Guo K, Ren J, Li G, Hu Q, Wu X, Wang Z, et al. Risk factors of surgical site infections in patients with Crohn's disease complicated with gastrointestinal fistula. Int J Colorectal Dis. 2017;32(5):635-43.

72. Nguyen DL, Ha I, Hogan C, Bechtold ML, Jamal MM. Pre-operative optimization of nutritional status using parenteral and enteral nutrition among Crohn's patients improves post-operative outcomes: A meta-analysis. Gastroenterology. 2016;1):S425.

73. Patel KV, Sandall AM, O'Hanlon DV, Darakhshan AA, Williams AB, Anderson SH, et al. Nutritional optimisation of presurgical Crohn's disease patients with enteral nutrition significantly decreases length of stay and need for a stoma. J Crohns Colitis. 2016;10 (Supplement 1):S33.

74. Smedh K, Andersson M, Johansson H, Hagberg T. Preoperative management is more important than choice of sutured or stapled anastomosis in Crohn's disease. Eur J Surg. 2002;168(3):154-7.

75. Abdalla S, Benoist S, Maggiori L, Zerbib P, Lefevre JH, Denost Q, et al. Impact of preoperative enteral nutritional support on postoperative outcome in patients with Crohn's disease complicated by malnutrition: Results of a subgroup analysis of the nationwide cohort registry from the GETAID Chirurgie group. Colorectal Dis. 2021;23(6):1451-62.

76. Fiorindi C, Cuffaro F, Piemonte G, Cricchio M, Addasi R, Dragoni G, et al. Effect of long-lasting nutritional prehabilitation on postoperative outcome in elective surgery for IBD. Clin Nutr. 2021;40(3):928-35.

77. Gordon-Dixon A, Hampal R, Miah A, Webb-Butler S, Lewis W, Ross R, et al. Does exclusive enteral nutrition reduce the rate of stoma formation in patients requiring ileocolic resection for Crohn's disease? A single center experience. Clin Nutr ESPEN. 2021;44:282-6.

78. Wall C, Glyn T, Bissett I, Rowbotham D, Haines M, Gearry R, et al. Preoperative nutrition optimisation in nourished Crohn's disease patients: A feasibility randomised controlled trial. Colorectal Dis. 2022;24(SUPPL 1):144.

79. Weber AT, Nguyen MT, Sauk JS, Limketkai BN, Kwaan M. Impact of Perioperative Nutritional Support in Patients Undergoing Major Inflammatory Bowel Disease Surgery: A Nationwide Analysis. Gastroenterology. 2021;160(6 Supplement):S-871.

80. Voitk AJ, Echave V, Feller JH, Brown RA, Gurd FN. Experience with elemental diet in the treatment of inflammatory bowel disease. Is this primary therapy? Arch Surg. 1973;107(2):329-33.

81. Zerbib P, Koriche D, Truant S, Bouras AF, Vernier-Massouille G, Seguy D, et al. Pre-operative management is associated with low rate of post-operative morbidity in penetrating Crohn's disease. Aliment Pharmacol Ther. 2010;32(3):459-65.

82. Peters EG, Smeets BJJ, Nors J, Back CM, Funder JA, Sommer T, et al. Perioperative lipid-enriched enteral nutrition versus standard care in patients undergoing elective colorectal surgery (SANICS II): a multicentre, double-blind, randomised controlled trial. Lancet Gastroenterol Hepatol. 2018;3(4):242-51.

83. Marafini I, Salvatori S, Troncone E, Scarozza P, Fantini E, Monteleone G. No effect of a liquid diet in the management of patients with stricturing Crohn's disease. Int J Colorectal Dis. 2020;35(10):1881-5.

84. Costa-Santos MP, Palmela C, Torres J, Ferreira A, Velho S, Ouro S, et al. Preoperative enteral nutrition in adults with complicated Crohn's disease: Effect on disease outcomes and gut microbiota. Nutrition. 2020;70S:100009.

85. Ge X, Tang S, Yang X, Liu W, Ye L, Yu W, et al. The role of exclusive enteral nutrition in the preoperative optimization of laparoscopic surgery for patients with Crohn's disease: A cohort study. Int J Surg. 2019;65:39-44.

86. Saito K, Ota K, Takazoe M, Mineki M. Effect of synbiotics during the perioperative period in patients with Crohn's disease: A pilot study. Clin Nutr ESPEN. 2020;40:596.

87. Bergeron F, Bouin M, D'Aoust L, Lemoyne M, Presse N. Food avoidance in patients with inflammatory bowel disease: What, when and who? Clin Nutr. 2018;37(3):884-9.

88. Silk DB, Payne-James J. Inflammatory bowel disease: nutritional implications and treatment. Proc Nutr Soc. 1989;48(3):355-61.

89. Silk DB. Medical management of severe inflammatory disease of the rectum: nutritional aspects. Baillieres Clin Gastroenterol. 1992;6(1):27-41.

90. Spencer MP, Nelson H, Wolff BG, Dozois RR. Strictureplasty for obstructive Crohn's disease: the Mayo experience. Mayo Clin Proc. 1994;69(1):33-6.

91. Lim GB. Risk factors: Nuts reduce risk of cardiovascular disease. Nat Rev Cardiol. 2018;15(1):4.

92. Yamamoto T, Nakahigashi M, Umegae S, Kitagawa T, Matsumoto K. Acute duodenal Crohn's disease successfully managed with low-speed elemental diet infusion via nasogastric tube: a case report. World J Gastroenterol. 2006;12(4):649-51.

93. Bengtsson J, Adlerberth I, Ostblom A, Saksena P, Oresland T, Borjesson L. Effect of probiotics (Lactobacillus plantarum 299 plus Bifidobacterium Cure21) in patients with poor ileal pouch function: a randomised controlled trial. Scand J Gastroenterol. 2016;51(9):1087-92.

94. Kuisma J, Mentula S, Jarvinen H, Kahri A, Saxelin M, Farkkila M. Effect of Lactobacillus rhamnosus GG on ileal pouch inflammation and microbial flora. Aliment Pharmacol Ther. 2003;17(4):509-15.

95. Alles MS, Katan MB, Salemans JM, Van Laere KM, Gerichhausen MJ, Rozendaal MJ, et al. Bacterial fermentation of fructooligosaccharides and resistant starch in patients with an ileal pouch-anal anastomosis. Am J Clin Nutr. 1997;66(5):1286-92.

96. Friedman G, George J. Treatment of refractory pouchitis with prebiotics and probiotic therapy. Gastroenterology. 2010;118(4):A778.

97. Godny L, Maharshak N, Yahav L, Fliss-Isakov N, Gophna U, Tulchinsky H, et al. Fruit consumption may protect against the development of intestinal inflammation via modification of microbial composition. J Crohns Colitis. 2016;10 (Supplement 1):S441.

98. Ianco O, Tulchinsky H, Lusthaus M, Ofer A, Santo E, Vaisman N, et al. Diet of patients after pouch surgery may affect pouch inflammation. World J Gastroenterol. 2013;19(38):6458-64.

99. McKinley JM, Ingram S, Vasey F, Sharma NR, Thareja S, Carter J, et al. Are inflammatory bowel disease (IBD) and pouchitis a reactive enteropathy to group d streptococci (Enterococci)? Gastroenterology. 2009;1):A663-A4.

100. Busacca CA, Milligan JA, Rattanangkool E, Ramavarapu C, Chen A, Saha AK, et al. Synthesis of phosphaguanidines by hydrophosphination of carbodiimides with phosphine boranes. J Org Chem. 2014;79(20):9878-87.

101. Roche RC, Harvey CV, Harvey JJ, Kavanagh AP, McDonald M, Stein-Rostaing VR, et al. Recreational Diving Impacts on Coral Reefs and the Adoption of Environmentally Responsible Practices within the SCUBA Diving Industry. Environ Manage. 2016;58(1):107-16.

102. Ellegard L, Bosaeus I, Andersson H. Will recommended changes in fat and fibre intake affect cholesterol absorption and sterol excretion? An ileostomy study. Eur J Clin Nutr. 2000;54(4):306-13.

103. Ellegard L, Andersson H. Oat bran rapidly increases bile acid excretion and bile acid synthesis: an ileostomy study. Eur J Clin Nutr. 2007;61(8):938-45.

104. Orsini F, Sello G, Sisti M. Aminophosphonic acids and derivatives. Synthesis and biological applications. Curr Med Chem. 2010;17(3):264-89.

105. Jeppesen PB, Mortensen PB. The influence of a preserved colon on the absorption of medium chain fat in patients with small bowel resection. Gut. 1998;43(4):478-83.

106. Kennedy HJ, Callender ST, Truelove SC, Warner GT. Haematological aspects of life with an ileostomy. Br J Haematol. 1982;52(3):445-54.

107. Guglielmi G, Vinuela F. Est modus in rebus. AJNR Am J Neuroradiol. 2014;35(1):E1.

108. Miettinen TA, Peltokallio P. Bile salt, fat, water, and vitamin B 12 excretion after ileostomy. Scand J Gastroenterol. 1971;6(6):543-52.

109. Culkin A, Gabe SM, Nightingale JM. A randomised controlled crossover trial to investigate the efficacy of a new oral rehydration solution for patients with a high output stoma. Clin Nutr. 2014;1):S45.

110. Barrett JS, Gearry RB, Muir JG, Irving PM, Rose R, Rosella O, et al. Dietary poorly absorbed, short-chain carbohydrates increase delivery of water and fermentable substrates to the proximal colon. Aliment Pharmacol Ther. 2010;31(8):874-82.

111. Bury KD, Stephens RV, Cha CJ, Randall HT. Chemically defined diets. Can J Surg. 1974;17(3):124-34.

112. Glossop NR, Lyons LC, Hardin PE. Interlocked feedback loops within the Drosophila circadian oscillator. Science. 1999;286(5440):766-8.

113. Dardai E, Pirityi S, Nagy L. Parenteral and enteral nutrition and the enterocutaneous fistula treatment. II. Factors influencing the outcome of treatment. Acta Chir Hung. 1991;32(4):305-18.

114. Hill GL, Goligher JC, Smith AH, Mair WS. Long term changes in total body water, total exchangable sodium and total body potassium before and after ileostomy. Br J Surg. 1975;62(7):524-7.

115. Ortiz LA, Zhang B, McCarthy MW, Kaafarani HMA, Fagenholz P, King DR, et al. Treatment of Enterocutaneous Fistulas, Then and Now. Nutr Clin Pract. 2017;32(4):508-15.

116. Wu XL, Chen RP, Tao LP, Wu JS, Chen XR, Chen WC. Infliximab Combined with Enteral Nutrition for Managing Crohn's Disease Complicated with Intestinal Fistulas. Gastroenterol Res Pract. 2016;2016:5947926.

117. Araujo EC, Suen VM, Marchini JS, Vannucchi H. Muscle mass gain observed in patients with short bowel syndrome subjected to resistance training. Nutr Res. 2008;28(2):78-82.

118. Atia A, Girard-Pipau F, Hebuterne X, Spies WG, Guardiola A, Ahn CW, et al. Macronutrient absorption characteristics in humans with short bowel syndrome and jejunocolonic anastomosis: starch is the most important carbohydrate substrate, although pectin supplementation may modestly enhance short chain fatty acid production and fluid absorption. J Parenter Enteral Nutr. 2011;35(2):229-40.

119. Byrne TA, Morrissey TB, Nattakom TV, Ziegler TR, Wilmore DW. Growth hormone, glutamine, and a modified diet enhance nutrient absorption in patients with severe short bowel syndrome. J Parenter Enteral Nutr. 1995;19(4):296-302.

120. Canada T. Teduglutide: A novel recombinant analog of human glucagon-like peptide-2 for short bowel syndrome. Formulary. 2012;47(9):314-5.

121. Hylander E, Jarnum S, Keldsbo IL, Thale M. Standardized ("trifixed") diet in the study of chronic malabsorption syndromes. Scand J Gastroenterol. 1978;13(4):423-31.

122. Jeppesen PB. Teduglutide, a novel glucagon-like peptide 2 analog, in the treatment of patients with short bowel syndrome. Therap Adv Gastroenterol. 2012;5(3):159-71.

123. Kunkel D, Basseri B, Low K, Lezcano S, Soffer EE, Conklin JL, et al. Efficacy of the glucagon-like peptide-1 agonist exenatide in the treatment of short bowel syndrome. Neurogastroenterol Motil. 2011;23(8):739-e328.

124. Lange S, Bosaeus I, Jennische E, Johansson E, Lundgren BK, Lonnroth I. Food-induced antisecretory factor activity is correlated with small bowel length in patients with intestinal resections. APMIS. 2003;111(10):985-8.

125. Messing B, Pigot F, Rongier M, Morin MC, Ndeindoum U, Rambaud JC. Intestinal absorption of free oral hyperalimentation in the very short bowel syndrome. Gastroenterology. 1991;100(6):1502-8.

126. Ovesen L, Chu R, Howard L. The influence of dietary fat on jejunostomy output in patients with severe short bowel syndrome. Am J Clin Nutr. 1983;38(2):270-7.

127. Pagoldh M, Eriksson A, Heimtun E, Kvifors E, Sternby B, Blomquist L, et al. Effects of a supplementary diet with specially processed cereals in patients with short bowel syndrome. Eur J Gastroenterol Hepatol. 2008;20(11):1085-93.

128. Scolapio JS. Treatment of short-bowel syndrome. Curr Opin Clin Nutr Metab Care. 2001;4(6):557-60.

129. Scolapio JS, McGreevy K, Tennyson GS, Burnett OL. Effect of glutamine in short-bowel syndrome. Clin Nutr. 2001;20(4):319-23.

130. Small M, Brundrett D, Nightingale JM. Long term subcutaneous saline and magnesium administration in patients with a short bowel. 10 year outcomes. Gut. 2015;64(Suppl 1):A315.

131. Stoidis CN, Misiakos EP, Patapis P, Fotiadis CI, Spyropoulos BG. Potential benefits of pro- and prebiotics on intestinal mucosal immunity and intestinal barrier in short bowel syndrome. Nutr Res Rev. 2011;24(1):21-30.

132. Weiming Z, Ning L, Jieshou L. Effect of recombinant human growth hormone and enteral nutrition on short bowel syndrome. J Parenter Enteral Nutr. 2004;28(6):377-81.

133. Wu GH, Wu ZH, Wu ZG. Effects of bowel rehabilitation and combined trophic therapy on intestinal adaptation in short bowel patients. World J Gastroenterol. 2003;9(11):2601-4.

134. Cox SR, Prince AC, Myers CE, Irving PM, Lindsay JO, Lomer MC, et al. Fermentable Carbohydrates [FODMAPs] Exacerbate Functional Gastrointestinal Symptoms in Patients With Inflammatory Bowel Disease: A Randomised, Double-blind, Placebo-controlled, Cross-over, Re-challenge Trial. J Crohns Colitis. 2017;11(12):1420-9.

135. Curro D, Ianiro G, Pecere S, Bibbo S, Cammarota G. Probiotics, fibre and herbal medicinal products for functional and inflammatory bowel disorders. Br J Pharmacol. 2017;174(11):1426-49.

136. Gearry RB, Irving PM, Barrett JS, Nathan DM, Shepherd SJ, Gibson PR. Reduction of dietary poorly absorbed short-chain carbohydrates (FODMAPs) improves abdominal symptoms in patients with inflammatory bowel disease-a pilot study. J Crohns Colitis. 2009;3(1):8-14.

137. Hallert C, Bjorck I, Nyman M, Pousette A, Granno C, Svensson H. Increasing fecal butyrate in ulcerative colitis patients by diet: controlled pilot study. Inflamm Bowel Dis. 2003;9(2):116-21.

138. Ibanez P, Ortega J, Tobar H, Solari S, Allende FA, Alvarez M. Inflammatory bowel disease activity is inversely correlated with vitamin d level in chilean patients. Gastroenterology. 2015;1):S454.

139. Ibanez P, Vila N, Santesteban V, Angos JR, Betes MT, Carretero C, et al. Patients with functional bowel disorder: Effectiveness of a diet low in FODMAPs for the treatment of gastrointestinal symptoms. Eur J Clin Invest. 2018;48 (Supplement 1):192.

140. Komperod MJ, Sommer C, Mellin-Olsen T, Iversen PO, Roseth AG, Valeur J. Persistent symptoms in patients with Crohn's disease in remission: An exploratory study on the role of diet. Scand J Gastroenterol. 2018;53(5):573-8.

141. Maagaard L, Ankersen DV, Vegh Z, Burisch J, Jensen L, Pedersen N, et al. Follow-up of patients with functional bowel symptoms treated with a low FODMAP diet. World J Gastroenterol. 2016;22(15):4009-19.

142. Nawawi KN, Belov M, Goulding C. Low fodmaps diet significantly improves ibs symptoms: An irish retrospective cohort study. Gut. 2017;66 (Supplement 2):A112.

143. Pedersen N, Ankersen DV, Felding M, Wachmann H, Vegh Z, Molzen L, et al. Low-FODMAP diet reduces irritable bowel symptoms in patients with inflammatory bowel disease. World J Gastroenterol. 2017;23(18):3356-66.

144. Prince AC, Myers CE, Joyce T, Irving P, Lomer M, Whelan K. Fermentable Carbohydrate Restriction (Low FODMAP Diet) in Clinical Practice Improves Functional Gastrointestinal Symptoms in Patients with Inflammatory Bowel Disease. Inflamm Bowel Dis. 2016;22(5):1129-36.

145. Sheasgreen C, Mohammadi A, Boland K, Atwal S, Borowski K, Stempak JM, et al. Increased dietary carbohydrate is associated with clinical activity and with endoscopic remission in inflammatory bowel disease. Gastroenterology. 2017;152 (5 Supplement 1):S421.

146. Swanson GR, Tieu V, Shaikh M, Forsyth C, Keshavarzian A. Is moderate red wine consumption safe in inactive inflammatory bowel disease? Digestion. 2011;84(3):238-44.

147. Tapete G, De Bortoli N, Ceccarelli L, Mumolo MG, Vinci E, Albano E, et al. Low-FODMAPs diet improves intestinal symptoms in IBD patients with disease remission: Randomized case-control study. Dig Liver Dis. 2018;50 (2 Supplement 1):e195.

148. Testa A, Imperatore N, Rispo A, Rea M, Tortora R, Nardone OM, et al. Beyond Irritable Bowel Syndrome: The Efficacy of the Low Fodmap Diet for Improving Symptoms in Inflammatory Bowel Diseases and Celiac Disease. Dig Dis. 2018;36(4):271-80.

149. Albrecht U, Muller V, Schneider B, Stange R. Efficacy and safety of a herbal medicinal product containing myrrh, chamomile and coffee charcoal for the treatment of gastrointestinal disorders: a non-interventional study. BMJ Open Gastroenterol. 2014;1(1):e000015.

150. Marotta F, Naito Y, Tajiri H, Lighthouse J, Yoshioka M, Ogliari C, et al. Disrupted mucosal barrier in quiescent ulcerative colitis: Effect of metronidazole and of a symbiotic preparation in a pilot cross-over study. Chinese J Dig Dis. 2003;4(4):180-5.

151. McNelly A, Nathan I, Monti M, Grimble G, Norton C, Bredin F, et al. Inflammatory bowel disease and fatigue: The effect of physical activity and/or omega-3 supplementation. J Crohns Colitis. 2016;10 (Supplement 1):S370-S1.

152. Raftery T, Martineau A, Greiller C, Lee CS, McNamara D, Ghosh S, et al. Does vitamin D supplementation impact plasma cathelicidin, human beta defensin 2 and intestinal permeability in stable Crohn's disease?-Results from a randomised, double blind placebo controlled study. Proc Nutr Soc. 2013;72 (OCE3):E176.

153. Scholten AM, Vermeulen E, Dhonukshe-Rutten RAM, Verhagen T, Visscher A, Olivier A, et al. Surplus vitamin B12 use does not reduce fatigue in patients with Irritable Bowel Syndrome or inflammatory bowel disease: A randomized double-blind placebo-controlled trial. Clinical Nutrition ESPEN. 2017.

154. Azpiroz F, Hernandez C, Guyonnet D, Accarino A, Santos J, Malagelada JR, et al. Effect of a low-flatulogenic diet in patients with flatulence and functional digestive symptoms. Neurogastroenterol Motil. 2014;26(6):779-85.

155. Chiba M, Nakane K, Tsuji T, Tsuda S, Ishii H, Ohno H, et al. Relapse prevention in ulcerative colitis through educational hospitalization providing plant-based diet experience. J Gastroenterol Hepatol. 2017;32 (Supplement 3):107.

156. Sabino J, Vieira-Silva S, MacHiels K, Joossens M, Falony G, Ferrante M, et al. The FIT trial: Anti-inflammatory dietary intervention effects on the intestinal microbiota. J Crohns Colitis. 2017;11 (Supplement 1):S473.

157. Cockerell KM, Watkins AS, Reeves LB, Goddard L, Lomer MC. Effects of linseeds on the symptoms of irritable bowel syndrome: a pilot randomised controlled trial. Journal of human nutrition and dietetics : the official journal of the British Dietetic Association. 2012;25(5):435-43.

158. Gilmore K, Burnett BP. Management of fecal incontinence in patients administered a medical food: Serum-derived bovine immunoglobulin/protein isolate. Gastroenterology. 2016;1):S893-S4.

159. Mutlu E, Mikolaitis S, Sedghi S, Chakradeo PS, Engen P, Chlipala G, et al. Dietary treatment of Crohn's disease: A randomized, placebo-controlled, double-blinded clinical trial. Gastroenterology. 2016;1):S778.

160. Afsar O, Tirnakli U. Probability densities for the sums of iterates of the sine-circle map in the vicinity of the quasiperiodic edge of chaos. Phys Rev E Stat Nonlin Soft Matter Phys. 2010;82(4 Pt 2):046210.

161. Abbas Z, Yakoob J, Jafri W, Ahmad Z, Azam Z, Usman MW, et al. Cytokine and clinical response to Saccharomyces boulardii therapy in diarrhea-dominant irritable bowel syndrome: a randomized trial. Eur J Gastroenterol Hepatol. 2014;26(6):630-9.

162. Fujimori S, Gudis K, Mitsui K, Seo T, Yonezawa M, Tanaka S, et al. A randomized controlled trial on the efficacy of synbiotic versus probiotic or prebiotic treatment to improve the quality of life in patients with ulcerative colitis. Nutrition. 2009;25(5):520-5.

163. Krammer HJ, Kamper H, von Bunau R, Zieseniss E, Stange C, Schlieger F, et al. [Probiotic drug therapy with E. coli strain Nissle 1917 (EcN): results of a prospective study of the records of 3,807 patients]. Z Gastroenterol. 2006;44(8):651-6.

164. Kruis W. Specific probiotics or 'fecal transplantation'. Dig Dis. 2012;30(SUPPL. 3):81-4.

165. Campbell H, Escudier MP, Brostoff J, Patel P, Milligan P, Challacombe SJ, et al. Dietary intervention for oral allergy syndrome as a treatment in orofacial granulomatosis: a new approach? J Oral Pathol Med. 2013;42(7):517-22.

166. Campbell HE, Escudier MP, Milligan P, Challacombe SJ, Sanderson JD, Lomer MC. Development of a low phenolic acid diet for the management of orofacial granulomatosis. J Hum Nutr Diet. 2013;26(6):527-37.

167. Fitzpatrick LR, Karpa KD. Probiotic Treatment of Colitis in Animal Models and People. Bioactive Foods Promot Health2010. p. 571-87.

168. Goel R, Ormond M, Hullah E, Nayee S, Escudier M, Sanderson J. An evaluation study of lactobacillus brevis CD2 in orofacial granuolomatosis. J Crohns Colitis. 2015;1):S355-S6.

169. McCartan BE, Healy CM, Fitzpatrick L, McCreary CE, Flint SR, Rogers SC, et al. Orofacial granulomatosis-a case series study of 120 patients. Oral Dis. 2010;16 (6):550.

170. Patel P, Brostoff J, Campbell H, Goel RM, Taylor K, Ray S, et al. Clinical evidence for allergy in orofacial granulomatosis and inflammatory bowel disease. Clin Transl Allergy. 2013;3(1):26.
